# Combining self-regulation and poverty reduction to prevent depression and anxiety among adolescents experiencing multi-dimensional poverty in Colombia, Nepal and South Africa: study protocol of a pilot 4-arm cluster randomised controlled trial

**DOI:** 10.1186/s40814-026-01858-y

**Published:** 2026-06-08

**Authors:** Mark J.D. Jordans, Crick Lund, Emily C Garman, Manuel Seifert Bonifaz, Mauricio Avendano, Annette Bauer, María Cecilia Dedios Sanguineti, Georgia Eleftheriou, Sara Evans-Lacko, Sandra García Jaramillo, Juan Felipe García, Kamal Gautam, Martin Gevonden, Roxanne Jacobs, Brandon A. Kohrt, Lydia Krabbendam, Nagendra P. Luitel, Nicci van der Merwe, Juan Jose Castellanos Piedrahita, Rakesh Singh, Mohammadamin Sinichi, Daniela Trujillo, Katherine Sorsdahl, Sanchari Roy, Wietse A. Tol, Daniela Rojas, Paula Yarrow

**Affiliations:** 1 Center for Global Mental Health, Health Service and Population Research Department, Institute of Psychiatry, Psychology and Neuroscience, Kings College London, 16 De Crespigny Park, London, SE5 8AB UK; 2 Alan J Flisher Centre for Public Mental Health, University of Cape Town, Department of Psychiatry and Mental Health, Cape Town, South Africa; 3 Center for Primary Care and Public Health (Unisanté), University of Lausanne, Lausanne, Switzerland; 4 Department of Social and Behavioral Sciences, Harvard School of Public Health, Boston, USA; 5 Care Policy and Evaluation Centre, London, School of Economics and Political Science, London, UK; 6 School of Government, Universidad de los Andes, Bogotá, Colombia; 7 Department of Mental Health, Johns Hopkins Bloomberg School of Public Health, Baltimore, USA; 8 Center for Global Mental Health Equity, George Washington University, Washington, DC USA; 9 Innovations for Poverty Action (IPA) Colombia, Bogotá, Colombia; 10 Transcultural Psychosocial Organization (TPO) Nepal, Baluwatar Kathmandu, Nepal; 11 Department of Biological Psychology, Faculty of Behavioural and Movement Sciences, Vrije Universiteit Amsterdam, Amsterdam, The Netherlands; 12 Department Clinical Neuro- and Developmental Psychology, Faculty of Behavioural and Movement Sciences, Vrije Universiteit Amsterdam, Amsterdam, The Netherlands; 13 Waves for Change, Muizenberg, South Africa; 14 War Child Alliance, Bogotá, Colombia; 15 Center for Global Mental Health, Institute of Psychiatry Psychology & Neuroscience, King’s College London, London, UK; 16 Department of Research, Transcultural Psychosocial Organization (TPO) Nepal, Kathmandu, Nepal; 17 Institute of Psychiatry, Psychology & Neuroscience, King’s College London, London, UK; 18 Department of Economics, University of Exeter, Exeter, UK; 19 Department of International Development, King’s College London, London, UK; 20 Section of Global Health, Department of Public Health, University of Copenhagen, Copenhagen, Denmark; 21 Athena Institute, Vrije Universiteit Amsterdam, Amsterdam, the Netherlands; 22 ARQ International, ARQ Psychotrauma Centre, Diemen, the Netherlands

**Keywords:** Adolescence, Anxiety, Depression, Prevention, Poverty, Self-regulation, Cash transfer

## Abstract

**Background:**

Poverty is associated with depression and anxiety among adolescents, but evidence of interventions that prevent adolescent depression and anxiety among adolescents living in poverty is weak. Interventions either focus on reducing poverty or addressing depression and anxiety, but an approach that combines both may offer larger benefit. This multi-site parallel pilot cluster randomised controlled trial (cRCT) evaluates the feasibility and acceptability of a selective preventive intervention for depression and anxiety that simultaneously intervenes on both poverty and self-regulation among adolescents living in urban poverty.

**Methods:**

The study takes place in Bogotá (Colombia), Kathmandu (Nepal) and Cape Town (South Africa). The pilot cRCT has four arms: (i) self-regulation intervention, (ii) economic intervention, (iii) combined (self-regulation + economic) intervention, and (iv) control group (care as usual). Interventions consist of 20 weekly group sessions with adolescents, and 6 monthly group sessions with their caregivers. The self-regulation intervention for adolescents includes psychological activities (mindfulness breathing, problem solving, biofeedback and goal setting) and a physical activity. The economic intervention includes three dimensions (financial training, negotiation training, education information) and a monthly cash transfer. In each site, the aim was to recruit 240 adolescents and their caregivers across eight schools (clusters), with two schools randomised to each arm. Adolescents residing in areas at risk of multidimensional poverty and who had symptoms of depression or anxiety, but who did not meet thresholds indicating depression and anxiety disorders, were considered for enrolment. Recruitment into the study is complete: a total of 628 adolescents and 536 caregivers were enrolled. Participants will be assessed four times: at enrolment, post-intervention and at 12 and 18 months post-enrolment. Primary outcomes include feasibility and acceptability criteria for study and intervention delivery procedures (i.e. randomisation, masking, recruitment, retention, missingness, fidelity, attendance, adverse events) defined as traffic light criteria to assess progression to an adequately powered trial. Secondary outcomes include self-report instruments, physiological measures and neuropsychological tasks.

**Discussion:**

The effectiveness of combining self-regulation and anti-poverty interventions remains untested. This study will establish the feasibility and acceptability of delivering such an intervention, as well as test the trial procedures to inform a future adequately powered trial.

**Trial registration:**

ISRCTN 14601588. Retrospectively registered. Date: 19 May 2024.

**Supplementary Information:**

The online version contains supplementary material available at 10.1186/s40814-026-01858-y.

## Background

Globally, 30.5% of the world’s adolescents are multidimensionally poor [1]. There is emerging evidence of the bidirectional causal mechanisms linking poverty with mental ill-health, particularly on the causal effect of poverty on adolescent and later life depression and anxiety [2]. Furthermore, there is evidence suggesting that neuropsychological processes of self-regulation mediate the influence of poverty on mental health [3]. Depression and anxiety are the leading contributors to the global burden of disease among young people, and contribute significantly to suicide [4]. Investing in the health and human capital of young people, 90% of whom live in low- and middle-income countries (LMICs) [5], is critical for reducing poverty and improving mental health for future generations. Yet, the evidence base for interventions that effectively prevent adolescent depression and anxiety is weak, particularly in LMICs [6, 7].

One major shortcoming is that interventions often fail to address socioeconomic determinants of adolescent depression and anxiety [8]. Indeed, there is growing evidence of the effect of poverty on adolescent and later life depression and anxiety [2]. Some antipoverty interventions, such as cash transfers and economic empowerment, have shown modest benefits for reducing depression among young people living in LMICs [9, 10]. A potential, yet untested hypothesis is that poverty reduction or economic empowerment interventions may have larger effects to reduce depression and anxiety if combined with psychological interventions; and in turn, psychosocial interventions may have larger effects if combined with poverty reduction and economic empowerment interventions [3].

Self-regulation is a particularly promising intervention target given the association between poverty and self-regulation, as well as self-regulation and depression. Here, we refer to self-regulation as the ability to set and maintain goal-directed behaviour in the face of distractors, particularly in emotionally salient contexts, and to flexibly adapt behaviour based on new information. According to Palacios-Barrios and Hanson [11], there is emerging evidence that poverty influences higher-level cognitive control, or “top-down” self-regulation processes (executive attention, decision-making and emotion regulation) and stimulus-driven reactive, or “bottom-up” self-regulation processes (stimulus generalisation, contextual processing and salience evaluation and interpretation). Disturbed self-regulation increases risk for adolescent depression and anxiety. In turn, disrupted self-regulation (and associated adolescent depression and anxiety) could influence the ability of young people to succeed in their education or life goals and escape poverty, forming a negative feedback loop.

A psychosocial intervention that strengthens self-regulation may therefore reduce risk for depression and anxiety, enable adolescents to succeed in their education or life goals and increase the likelihood of escaping poverty in the longer term. Likewise, an intervention that improves economic circumstances may address depression and anxiety by reducing family financial stress, increasing a sense of control and security, enhancing educational opportunities, increasing hope and future orientation, improving peer and family relationships, developing life skills, and increasing resilience to adverse environments. Thus, combining an economic intervention with a psychosocial intervention to strengthen self-regulation could jointly prevent adolescent depression and anxiety, and the combination of these strategies may have a larger impact on reducing the incidence of depression and anxiety among adolescents than either of these interventions alone.

### Objectives

The primary aim of this study is to evaluate the feasibility and acceptability of an intervention that combines an economic intervention with a self-regulation intervention among adolescents living in urban poverty in Bogotá (Colombia), Kathmandu (Nepal) and Cape Town (South Africa). This will be done by assessing the following criteria (which have been defined as progression to full trial criteria): (1) fidelity across intervention arms; (2) participants attending sufficient intervention sessions; (3) reported adverse events across intervention arms compared to control arm; (4) reported negative experiences related to cash transfers, as well as qualitative perspectives on acceptability of interventions; (5) baseline differences in demographic variables across arms for adequacy of randomisation; (6) adequacy of masking; (7) missing values on outcome instruments; (8) loss to follow-up across timepoints. Furthermore, we aim to evaluate the sensitivity to change on study participants’ clinical and non-clinical outcomes. The study is part of the ‘Improving Adolescent mentaL health by reducing the Impact of poVErty (ALIVE)’ project, which has been introduced elsewhere [12].

## Methods/design

### Design

We will conduct a pilot parallel 4-arm cluster randomised controlled trial (cRCT). The pilot cRCT will include the following four arms: (i) self-regulation intervention, (ii) economic intervention, (iii) combined (self-regulation + economic) intervention and (iv) control group. A cluster design was selected because of the potential contamination of implementation strategies among adolescents within the same school. The assessment of the feasibility criteria is the primary outcome of the study. The assessment of the sensitivity to change on self-report and neuropsychological instruments is the secondary outcome. Semi-structured qualitative interviews will also be conducted with key informants to assess perspectives on the study procedures, as well as intervention content and delivery.

### Study settings

The study will take place in three settings: Bogotá (Colombia), Kathmandu (Nepal) and Cape Town (South Africa). All three urban settings have large populations of adolescents living in diverse circumstances of multi-dimensional poverty. Colombia ranks among the world’s most unequal countries, with a Gini coefficient of 0.52. More than one third (34%) of children and adolescents live in poverty [13] and 19% of 15–19-year-old adolescents are not in employment, education or training [14]. There remain high rates of violence and displacement linked to violence and armed conflict, with 22.6% of adolescents suffering from common mental health problems [15]. Nepal is one of the poorest countries in south Asia: based on gross national income per capita, Nepal ranks 168 out of 226 countries globally [16]. Adolescents in Nepal are at high risk of depression and anxiety, due to a complex dynamic of recent and historical trauma (two major earthquakes in 2015 and a 10-year civil war between 1996 and 2006) and socio-economic deprivation. Only 54% enrol in secondary school and, among girls, early marriage is a common reason for leaving school [17]. South Africa has one of the highest youth unemployment rates in the world, with 35.5% of 15–24-year-old youth not engaged in employment, education or training [18]. It is also one of the most unequal countries in the world with a Gini coefficient of 0.63, and persistent child and adolescent poverty; 46% of youth are considered multidimensionally poor [19]. Recent school-based studies in the Western Cape have shown that more than half of 15–18-year-olds screen positive for depression or anxiety [20], while 33% and 21% of adolescents aged 10–14 years screened positive for depression and anxiety, respectively [21].

### Interventions

All three interventions consist of 20 weekly sessions for groups of adolescents and 6 monthly group sessions with primary caregivers (one per adolescent participant), all delivered over a 4–6-month period. Sessions last approximately 1.5 h in the economic and self-regulation interventions, while they last approximately 2 h in the combined intervention (see Table 1 for more details about the three active interventions), although small deviations in duration are acceptable depending on local circumstances. We included primary caregivers in all three intervention arms, following the findings of our qualitative formative research in each ALIVE site, which informed us that changes in the self-regulation or economic circumstances of the adolescent are likely to be greatly enhanced by supporting a change in their home environment, through either improved economic circumstances in the household, improved co-regulation by caregivers, or a combination of the two.

**Table 1:** Overview of the intervention

Intervention	Population	Participants per group *	Sessions	Session duration	Location of delivery	Content of intervention
Economic intervention	Adolescents	15	20	1.5 h	Community, or school hall	• Monthly cash transfer • Phase 1 (12 sessions): Financial education (developing skills to improve short- and long-term handling of finances) • Phase 2 (3 sessions): Negotiation (exploring opportunities through bargaining/negotiation and findings solutions) • Phase 3 (5 sessions): Information on education (creating awareness around benefits, challenges and solutions for accessing education)
	Caregivers	15	6	1.5 h	Community, or school hall	• Monthly cash transfer • Phase 1 (4 sessions) • Phase 2 (1 sessions) • Phase 3 (1 sessions)
Self-regulation intervention	Adolescents	15	20	1.5 h	Sports or school hall^a^	• Phase 1 (4 sessions): Facilitator behaviour to create conditions to support youth wellbeing • Phase 2 (5 sessions): Building strong connections • Phase 3 (7 sessions): Building strong minds • Phase 4 (4 sessions): Building strong sense of future
	Caregivers	15	6	1.5 h	Community, or school hall	Based on Caregiver Support Intervention; includes: • Psychoeducation • Parenting and communication techniques • Stress reduction
Combined intervention	Adolescents	15	20	2 h	Sports or school hall^a^	Condensed or abbreviated content of economic and self-regulation interventions, including section to explore connections between the two interventions
	Caregivers	15	6	2 h	Community, or school hall	

^*^Approximate number

^a^beach in South Africa

### Economic intervention

For both adolescents and caregivers, the economic intervention is divided into three phases. The first phase (financial education) focuses on providing education specific to financial needs and wants and developing skills to improve short- and long-term understanding and handling of finances, for both parents and adolescents. The second phase (negotiation training) aims to help adolescents develop negotiation and interpersonal skills to increase strategic cooperation and adolescent’s bargaining power in negotiations [22, 23]. This builds on recent research that emphasises the interdependence of decision making within the household, and the importance of adolescent negotiation skills to elicit investments from their parents in their education and well-being. The third phase (information on education) focuses on creating awareness and practical knowledge on the benefits, challenges and solutions for accessing education. Alongside the delivery of the intervention sessions, both adolescents and caregivers receive 6 unconditional cash transfers over a 4–6-month period (see Table 2), which altogether equate to each country’s 1 month’s average household income. One third of the cash transfer is allocated to the adolescent and two thirds to the caregiver.

**Table 2:** Monthly amount and method of cash transfer sent to participants

	Amount to caregivers	Amount to adolescent	Transfer method
Colombia	38 GBP	19 GBP	Bank transfer*
Nepal	27 GBP	12 GBP	Cash in hand
South Africa	88 GBP	44 GBP	Instant money voucher

^*^After creating accounts for those who do not have one

### Self-regulation intervention

The self-regulation intervention combines group physical activity with core active components that strengthen self-regulation. These were selected based on an extensive review of the literature assessing the effect of key intervention ingredients in preventing depression or anxiety among youth globally [24, 25]. The purpose of the self-regulation intervention is to strengthen self-regulation among adolescents living in circumstances of poverty, through (i) the creation of safe spaces for youth to build strong social connections, (ii) experience respite from adversity and stressors and (iii) learn and master new skills (skateboarding (Colombia), martial arts-based activity (Nepal) and surfing (South Africa)). The self-regulation intervention includes four phases: phase 1 focuses on facilitator behaviours to create the conditions that support and boost youth wellbeing. The following phases focus on behaviours adolescents can practice and master in a safe and supportive environment to build on strong connections (phase 2), strong minds (phase 3) and strong sense of future (phase 4). These include building trust, mindfulness breathing, social skills, problem solving, biofeedback and goal setting for the adolescents. The caregiver part of the intervention covers elements of psychoeducation, parenting and communication techniques, and stress reduction, based on the Caregiver Support Intervention [26, 27]. This is in keeping with Murray, Rosanbalm [28]’s suggestion for a supporting environment, including caregiver support, for adolescents to learn and apply self-regulation skills.

### Combined intervention

The combined intervention, which integrates economic and self-regulation strategies, draws from the two previously described interventions, including the cash transfer component. While content was maintained, to fit within the intended 2-h duration of each session, it was condensed through a process that included (1) identifying the most relevant activities from each curriculum; (2) identifying common themes across pairs of sessions in the self-regulation and economic intervention curricula that could be taught in a single session. Each session also includes a section where adolescents are encouraged to discuss the connections between the economic and self-regulation contents of the intervention. Together these economic and self-regulation strategies are designed to be mutually reinforcing, with the economic support facilitating application of self-regulation skills.

### Control condition

The control group follows a “care as usual” approach, which means it is a no active comparator condition. This is in line with a decision framework for the selection of control condition in randomised controlled trials [29], considering the ALIVE interventions can be categorised as an early phase trial and as low-risk. We also want to compare our intervention arms with the reality of what adolescents currently receive in these communities. Finally, there is genuine equipoise about the effects of self-regulation and economic interventions (including cash transfers) on the prevention of depression and anxiety. This also justifies not giving cash to the self-regulation and control arms at the end of the intervention.

### Training and supervision of intervention facilitators

A cascading model to capacity building was followed to train the facilitators in the three active interventions. The intervention developers served as master-trainers and conducted a 5-day Training-of-Trainers to two trainers per intervention per country in Cape Town in February 2024. For each country, the criteria for the trainers were established to match the content of the interventions, bearing in mind the different cadre of staff and resources available in each country. The two trainers per intervention arm per country subsequently trained 6 facilitators per intervention during a 5–6-day training in each country (See Supplementary Tables 1 and 2 for an overview of characteristics of trainers and facilitators). Dyads of trained facilitators were then paired to a school for the implementation of the intervention.

During the implementation of the three active interventions, in-person supervision sessions with facilitators are organised by the in-country trainers. A weekly supervision session is used to discuss the previous session, any difficulties experienced in the delivery of the intervention, and prepare the content of the next session, including practising key activities using role plays. For facilitators specifically recruited to provide the caregiver sessions of the self-regulation and combined intervention in South Africa, supervision sessions occur twice monthly, with one session focusing on discussing the previous session, and the second session reviewing content of the next session. In addition, cross-country online supervision meetings with the trainer/supervisors from each country are conducted monthly, separately by arm, to review the progress of the intervention.

### Implementation

The implementation of the interventions, as well as the training and supervision of facilitators, is the responsibility of not-for-profit organisations in each country site, who are well established within the community, have experience in delivering psychosocial interventions within communities affected by poverty, and have strong ties with local leaders and organisations. These include the following: (1) War Child (Colombia), which is an international organisation that aims to improve resilience and wellbeing among conflict-affected children; (2) Transcultural Psychosocial Organisation (TPO) Nepal (Nepal), which is both a mental health research and service delivery organisation; (3) Waves for Change (W4C, South Africa), which provides child-friendly mental health services to children and young people in under-resourced communities through surfing; and Interfer (South Africa), which delivers community-based empowerment programmes for young people.

### Participant selection for cRCT

#### School eligibility and class selection

Schools are the unit of randomisation in this pilot cRCT. School eligibility and selection took place in a previous phase of the ALIVE project. A list of all eligible schools was drawn from each geographical study area, based on the criteria listed in Table 3. In South Africa, a list of all schools in the study area was established (*n* = 370), from which those falling within quintiles 1,2,3 were selected (*n* = 128) (categorised by the government as most impoverished [31]), from which those within 30 km from the implementing organisation (W4C) were selected for potential inclusion (*n* = 45). In Nepal, a list of all schools in the study area (Budhanilkantha Municipality and its adjacent wards from Kathmandu Metropolitan City) (*n* = 21) was established, from which schools were excluded following the in- and exclusion criteria (*n* = 13). In addition, two schools were excluded as this is where initial testing of instruments took place; the final sample of eligible schools being 11. In Colombia, a list of schools with the highest Multidimensional Poverty Indices in the districts of Usme and Ciudad Bolivar in Bogotá was established. The two districts consist of 6 and 5 geographical clusters, respectively, four of which were randomly selected in each district; this represented a total of 59 eligible schools (*n* = 19 in Usme; *n* = 40 in Ciudad Bolívar).

**Table 3:** Cluster eligibility criteria

School eligibility criteria	Colombia	Nepal	South Africa
Poverty	Schools located in the two districts of Usme and Ciudad Bolívar; categorised as high poverty areas [30]	Public schools selected from areas in Kathmandu that are characterised by higher risk of poverty	Quintile 1–3 schools; categorised by the government as most impoverished [31]
Distance	At least a 2-km distance between schools	At least 1 km distance between two public schools	Distance to Waves for Change (within 30 km from site) and less than 45 min to travel from school to intervention site
Grades covered	Schools that have all the grades from 6 to 11th on the same site Some schools in Colombia have different sites for separate levels (lower secondary vs upper secondary). For logistical purposes of the intervention, added eligibility criteria are therefore to have all secondary-school grades in the same school	Secondary-level public schools	Secondary-level public schools
Students per class	At least 30 students per grade	Number of students from grade 6, 7 and 8 for a school total equalling 100 or more	Size of school (at least 100 adolescents in each grade)
Space	n/a	Schools with at least one hall and a playground, and availability of separate toilets for girls and boys	n/a
Willingness to participate	Yes	Yes	Yes
Involvement in previous ALIVE study	Not the same children in schools that were involved in previous study	Not same site as where ALIVE instruments were validated	Not same site as where ALIVE instruments were validated

Once the pool of eligible schools was identified in each country site, eight schools in each site were randomly selected using a computer-based pseudo-random number generator. In South Africa, the random selection was stratified by the language most spoken in the school (isiXhosa or Afrikaans). In Nepal, the selection of schools was stratified by total class size for relevant grades (below vs above 200 learners). In Colombia, the selection was stratified by district (Usme vs. Ciudad Bolívar) and one school was randomly selected in each of the four clusters per district, thus ensuring the selected schools were at least 2 km apart.

### Randomisation into study arms

A simple random sampling approach was employed by a statistician (EG) to randomly allocate the eight schools to the four arms in each site, on a 1:1:1:1 ratio. The randomisation sequence was computer-generated using a pseudo-random number generator on Stata version 14 and completed before baseline assessments.

### Sample size

We aimed to recruit a sample of 240 adolescents (13 − 15 years), living in poor urban households in each country, with an equal sample size in each of the four trial arms (*n* = 60). This study is not powered to evaluate effectiveness [32] but instead aims to test trial procedures, based on which parameters can be defined to run sample size calculations for the adequately powered RCT. We followed recommendations for sample size requirements to estimate key design parameters for pilot trials, which suggest at least 240 measured participants (60 per group) when estimating the Experimental Event Rate [33]. Thus, we aimed to recruit 30 adolescents and their caregivers per school, for a total of 60 adolescents and their primary caregiver per arm.

### Participant recruitment

The recruitment of adolescents within clusters (schools) took place in two phases. In the first phase, approximately *n* = 500 adolescents and their caregivers per country site were enrolled from the eight schools, after selecting classes (per grade) representing the study age range of 13 − 15 years; this was performed as part of a cross-sectional study that aimed to identify associations among mental health, self-regulation and economic indicators and to establish the psychometric properties of instruments. In line with the definition of a selective prevention trial, the second phase involved randomly selecting 30 participants who were not at risk of depression or anxiety among those who were enrolled in the first phase cross-sectional survey. Again, the randomised selection of participants was completed by the statistician using a computer-based pseudo-random number generator.

### Inclusion and exclusion criteria

The inclusion criteria for adolescents, assessed during Phase 1 were:

1. High levels of multi-dimensional poverty; in Colombia and South Africa this was defined by the geographical catchment area of the selected schools. In Nepal, children attending public schools come from diverse backgrounds; thus, a short screening instrument was used during Phase 1 to identify children living in high levels of poverty in the selected schools before they were enrolled in the cross-sectional survey. This screening instrument is a short tool developed using items from the Global Multidimensional Poverty Index, poverty assessment-related literature, expert consultations and consultations with an Adolescent Advisory Group in Nepal.
2. Aged between 13 and 15 years at the time of Phase 1 recruitment.
3. Fluent in the predominant local language (Spanish in Colombia, Nepali in Nepal and English in South Africa).
4. Screening below validated, country-specific, cut-offs for depression and anxiety on the Patient Health Questionnaire – Adolescent version (PHQ-A) (for depression) [34] and Generalised Anxiety Disorder (GAD-7) (for anxiety) [35] screening instruments.
5. Whose primary caregiver is enrolled in the pilot study (in Colombia and South Africa).

During phase 2, if multiple children in one household fell into the 13–15 years age group, one index child was randomly selected by a member of the research team in each site. In Phase 2, the selected adolescents were screened again using the PHQ-A and GAD-7. Participants who screened *negative* for depression and anxiety were then enrolled in the study; those who screened positive were excluded. When it was not possible to conduct repeat assessments (in South Africa only), adolescents who screened negative on the PHQ-A and GAD-7 during Phase 1 were considered eligible, without a repeat screening assessment before enrolment.

The inclusion criteria for caregivers, defined as the legal guardian living with the adolescent, (assessed during Phase 1) include:

1. Being fluent in the local language (Spanish in Colombia, Nepali in Nepal, and English, Afrikaans or isiXhosa in South Africa).
2. Aged 18 years or more; with their consent to participate in the study.
3. Their child (biological or foster) is enrolled in the study.

Three exclusion criteria were employed during Phase 2 of recruitment:

1. Adolescents at high suicide risk, defined as reporting current suicidal ideation with intent or plans over the past month (all sites), or ever reporting suicidal attempt (Colombia) or reporting suicidal attempt in the past 3 months (Nepal and South Africa).
2. Adolescents or caregivers with a significant disability that impacts participation in intervention or assessments (that cannot be overcome with reasonable adjustments).
3. Unaccompanied minors and adolescents who are married, because of difficulties in obtaining legal consent of guardians.

### Consent and assent procedures for cRCT

Permission to conduct the pilot cRCT was first sought from the municipalities’ education unit’s authorities (Nepal) and the Department of Education (South Africa). In the case of Colombia, a memorandum of understanding was signed with the City’s Department of Education. Permission from the schools’ head teachers was then sought. Verbal consent to screen the adolescent was sought first by the research assistants, and written assent from adolescents who were eligible to be enrolled was then obtained, together with their caregiver’s consent. In Colombia, written assent from adolescents and written consent from caregivers was obtained before the screening. In cases of low literacy, the research assistants read the information letter out loud. Adolescents and caregivers were given up to 1 week to decide whether to participate in the study and were reminded that their decision to participate in the study would not affect their grades or treatment at school.

In Nepal, where the enrolment of the caregiver was not an inclusion criterion for adolescent participation, caregivers of adolescents enrolled in an active treatment arm received a short leaflet describing the intervention that their child was allocated to. Caregivers were given the opportunity to join the study within 1 week of receiving the information leaflet.

Potential participants will not be informed of the content of the other arms. This is important for preventing contamination (spillover effects), but also to avoid any undue repercussions on willingness to participate, attrition or outcomes should participants prefer the intervention provided in other arms. Participants will be debriefed about what the other arms consisted of by the research assistants immediately after the 18-month post baseline assessment.

### Status of recruitment

Recruitment into the trial started on 28 February 2024 (South Africa, as part of Phase 1 of recruitment) and lasted until 26 June 2024 (Colombia). At the date of submission of this paper, participants are no longer being enrolled into the study. See Table 4 for recruitment dates and the final sample of adolescents and caregivers recruited in each site.

**Table 4:** Final number of participants enrolled in the cRCT across sites

	**Colombia**	**Nepal**	**South Africa**	**Total**
Recruitment dates	6 March– 26 June 2024	17 March– 22 May 2024	28 February–19 June 2024	
Adolescents enrolled	160	229	239	628
Caregivers enrolled	160	141	235	536

### Participant selection for qualitative interviews

#### Sample

From among the pilot cRCT participants and implementers, a purposive sample will be drawn to conduct semi-structured, key informant interviews. We will interview the following: intervention facilitators (*n* = 6 per country; 2 from each of the three active trial arms); supervisors (*n* = 3 per country, 1 from each active trial arm); adolescents participating in the ALIVE interventions (*n* = 18 per country [9 completers, i.e. those that attended > 70% of sessions; and 9 non-completers, i.e. those that attended = < 70% of the sessions]; 6 from each of the active trial arms); caregivers participating in the ALIVE interventions (*n* = 18 per country, 6 from each of the active trial arms, not dependent on attendance); and adolescents allocated to the control arm (*n* = 3 per country). The aim is to get information of how ALIVE staff experienced the intervention implementation.

#### Consent and assent procedures

Intervention facilitators and supervisors will be approached to participate in interviews via an email or phone call by a researcher from the local research institution: Universidad de los Andes (Colombia), TPO Nepal (Nepal) and the University of Cape Town (South Africa). The email will contain the information sheet and consent form for staff members to review. They will be given the opportunity to ask questions via email or in person, and to send or give a signed copy of the consent if they agree to participate. For adolescents, the same recruitment procedures will be followed as detailed above. Caregivers purposefully selected for the interviews will be contacted telephonically and the study discussed over the phone. Again, we will give adolescents and caregivers 1 week to decide whether to participate in the study.

### Instruments

Four types of data are being collected: (1) intervention implementation outcomes (to assess feasibility); (2) adolescents’ outcomes (to assess sensitivity to change), including self-report instruments, physiological measures and neuropsychological tasks; (3) caregiver outcomes (all self-reported instruments, to assess sensitivity to change); and (4) costs (to assess feasibility). All self-reported instruments and neuropsychological tasks included in the assessment have been adapted in each site as part of the ALIVE project. These are described in more detail below and reported in Tables 5 and 6. Unless otherwise stated, all adolescent and caregiver outcomes are assessed at all timepoints. Full adolescent and caregiver assessments are also available in supplementary files 3 and 4.

**Table 5:** Overview of self-report adolescent instruments

Name	Domain	Construct	# Items	Responses/Scoring	Country/Regional Psychometrics	Time (minutes)
Demographics	Demographics	Age, gender, ethnicity, etc	17	Multiple response options; used as individual item covariates in analyses	Not applicable	2
Education (Risk score items: [36]) - Schooling	Education	Educational status, disruptions	16	Multiple options; used as individual item covariates in analyses; items contribute to calculation of IDEA Risk Score	Not applicable for individual items; IDEA-RS c-statistic described below	2
Measurement of Mental Health among Adolescents and Young People at the Population Level (MMAPP) [37]	Mental health	Anxiety, depression	28	Frequency of symptoms (4 options, 0 never to 3 always); Total PHQ-A and GAD-7 score; depression caseness (score > = 16 (South Africa), > = 15 (Nepal), > = 14 (Colombia)); anxiety caseness (score > = 12 (Colombia and South Africa), > = 10 (Nepal))^A^	Depression: Colombia (≥ 14): 0.72 (sens); 0.74 (spec) Nepal (≥ 15): 0.88 (sens); 0.83 (spec) SA (≥ 16): 0.75 (sens); 0.79 (spec) Anxiety: Colombia (≥ 12): 0.71 (sens); 0.76 (spec) Nepal (≥ 10): 0.58 (sens); 0.64 (spec) SA (≥ 12): 0.69 (sens); 0.73 (spec)	6
Disruptive Behaviour International Scale (DBIS) [38]	Mental Health	Oppositional defiant disorder, conduct disorder	8	Frequency of symptoms (4 options, 0 never to 3 always); Total disruptive behaviour symptom score (sum of all items)	Nepal only: Cronbach’s *α*: 0.84; test–retest reliability (intraclass correlation 0.93, *r* =.93) [38].	1
IDEA Risk Score (IDEA-RS) [36]^B^	Risk factors for development of depression	Quality of relationships, substance use, maltreatment	21	Yes/no responses for most questions; To calculate IDEA-RS, 11 items are summed (drawn from this section in addition to demographics and education)	AUC = 0.83 (Nepal; [36]), 0.69 [39], 0.66 (Nigeria; [40])	3
Difficulties in Emotion Regulation Scale (DERS) [41]	Emotion regulation	Awareness of and behavioural response to distress	15	Frequency (0 Never to 4 Always); sum score with reverse coding for select items	Cronbach’s α (Nepal 36-item version with adolescents) = 0.77 [42]	3
Children’s Hope Scale (CHS) [43]	Hope	Agency and pathways hope	6	Frequency (0 Never to 4 Always); Sum score for hope (agency subscore, pathways subscore)	Nepal: Cronbach’s *α* = 71 for agency and.78 for pathways, and test–retest *r* =.70; Metric and scalar invariance are not supported in Nepal (including notable gender differences) [44]; South Africa: English and Afrikaans versions—Cronbach’s α 0.73–0.82, with demonstrated scalar invariance [45]	1
Economic - Expectation and aspirations [46] - Knowledge about education - Financial literacy [47] - Negotiation skills [22]	Economic	Economic decision making, spending, and earning expectations	43	Various response options (e.g. categories for earning expectations; yes/no questions; and level of difficulty questions);		6
Client Service Receipt Inventory, adapted version [48]^C^	Cost consequences	Resource use	8	Response options (vary by question): Yes/No; number of/duration of visits; how much paid)		10
Child and Youth Resilience Measure (CYRM) (Nepal only) [49]	Resilience	Importance of education, food availability, feeling safe, social support	17	Amount (e.g. amount of food) or degree (e.g. how much people like spending time with the adolescent); scoring 1 = Not at all; 5 = A lot	Cronbach’s *α*: 0.84 (based on data from ALIVE survey)	2
Rugged Resilience Measure (RRM) [50] (Nepal only)	Resilience	Controlling emotions, responding to challenges, coping	10	Amount (1 = Not at all; 5 = A lot)	Cronbach’s α: 0.82 (based on data from ALIVE survey)	1

^A^Exact cut-offs for South Africa and Colombia were established after clinical validation study as part of ALIVE

^B^Not assessed at T0

^C^Only assessed at T2

**Table 6:** Overview of caregiver self-report instruments

Name	Domain	Construct	# Items	Responses/Scoring	Country/Regional Psychometrics	Time (minutes)
Alabama Parenting Questionnaire [51]	Parenting Behaviours	Parenting styles	16	Response options: 1 = never, 2 = almost never, 3 = sometimes, 4 = often, 5 = always	APQ in which moderate internal consistency of the Poor Monitoring/Supervision (alpha = 0.59) and Corporal Punishment (alpha = 0.55) was found. The Cronbach alpha for the other three subscales were much higher: Parental involvement = 0.75, Positive parenting = 0.77, and Inconsistent discipline = 0.73 [51]	3
Patient Health Questionnaire [52]	Mental health	Depressive symptoms	9	Response options: 0 = never, 1 = sometimes, 2 = often, 3 = always	PHQ-9 scores > 10 had a sensitivity of 88% and a specificity of 88% for Major Depressive Disorder. Reliability and validity of the tool have indicated it has sound psychometric properties. Internal consistency of the PHQ-9 has been shown to be high	2
Demographics	Demo-graphics	Age, ethnicity, residence, etc	5	Multiple response options; used as individual item covariates in analyses	Not applicable	1
Social and economic measures: - Migration - Welfare benefits; Income and dept; - Household expenditure; - Social security; - Aspirations and beliefs for child and for self [46] - Risk preferences - Social preferences	Socio-economic	Migration, welfare benefits, household income, dept and expenditure, social security etc	50	Various response options (e.g. categories for earning expectations; yes/no questions; and level of difficulty questions)	/	12
Multidimensional Poverty Index [30, 53, 54]	Poverty	household type, assets, access to water, sanitation etc	6–7 per country	Multiple options; items contribute to calculation of Multidimensional Poverty Index in each site		2
Household roster	Household member demographics, education and employment	Members’ age, gender, schooling, employment status, literacy	17 per member	Multiple options; certain items contribute to calculation of Multidimensional Poverty Index	/	6
Client Service Receipt Inventory, adapted version [48]	Cost consequences	Resource use	15	Response options (vary by question): Yes/No; number, duration, cost of visits	/	15

#### Assessment of intervention procedures

The study will employ three measures for the assessment of protocol adherence and quality control: (1) competency of facilitators, (2) fidelity to the intervention protocol and (3) attendance by adolescents and caregivers. First, the competency of facilitators will be assessed by trainers/supervisors using the WHO-UNICEF Ensuring Quality in Psychosocial and Mental Health Care Platform (EQUIP) [55]. On the EQUIP platform, competencies for working with children and adolescents will be assessed with the Working with Children Assessment of Competencies Tool (WeACT) [56]. Competencies for working with adult caregivers of the adolescents will be assessed with the 15-item Enhancing Assessment of Common Therapeutic factors (ENACT) tool [37]. The WeACT and ENACT instruments aim to assess common factors in psychological treatments, including task-sharing initiatives with non-specialists across diverse cultural settings. Competencies are assessed while observing culturally adapted, standardised role-plays pre- and post-training, as well as post-intervention, (for all three ALIVE intervention arms) with scoring done by a trained rater or the trainers/supervisors.

Second, the fidelity to intervention protocol will be assessed using self-developed instruments designed to assess the implementation of the ALIVE intervention (all three arms) to intervention protocols. These will be completed by one facilitator at each session, and by trainer/supervisors through observations of approximately 25% of sessions. The assessment of fidelity, as well as other procedures to implement the intervention, was streamlined across the sites (i.e. a joint training of trainers was held, same duration and content of the interventions).

Third, attendance of ALIVE intervention sessions by adolescents and caregivers will be monitored using TeamPact. TeamPact is a mobile app that can be used offline, which helps facilitators collect information on attendance and length of sessions, and allows facilitators to provide feedback on the sessions.

#### Outcome instruments for adolescents

The Patient Health Questionnaire (PHQ-9) Adolescent version (PHQ-A) [34] and Generalised Anxiety Disorder Scale (GAD-7) are the primary outcomes for a future adequately powered trial [35]. They will be assessed using the Measurement of Mental Health among Adolescents and Young People at the Population level Tool (MMAPP) [38], which has the ability to generate equivalency scores for the PHQ-A and the GAD-7. Cut-offs for the PHQ-A and the GAD-7 were previously validated in Nepal, and validations were conducted for Spanish in Colombia and English in South Africa as part of the ALIVE formative work. Based on these validations, cut-off thresholds were selected that minimised false positives. This is because the target group of the intervention is adolescents who do not have depression or anxiety. Sensitivity (and associated false negatives) was less pertinent to selection. (See Table 5 for country-specific cut-offs). The future primary outcome for the full RCT will be defined as the cumulative incidence of depression or anxiety, that is, scoring above depression and/or anxiety cut-offs at one or more of the follow-up assessments.

The self-report instruments for secondary mental health adolescent outcomes include the Disruptive Behaviour International Scale [57]; the Identifying Depression Early in Adolescence Risk Score [41]; the Difficulties in Emotion Regulation Scale – Short form [43]; the Children’s Hope Scale [49]; the Child and Youth Resilience Measure [50] (in Nepal only); and the Rugged Resilience Measure [46] (in Nepal only); and additional questions covering: demographics, school enrolment/education, aspirations and beliefs [47], child labour and financial education [58], negotiation skills [23] and choice regarding cash transfer. Additional secondary economic outcomes are also assessed, with a particular emphasis on outcomes related to education, financial literacy and negotiation skills, the three areas of focus of the intervention. These include:

- Schooling outcomes: This is assessed through continued school enrolment, school dropout, academic engagement and effort (measured by the number of hours spent on average on schoolwork outside of school, and skipping school) and school performance assessed through school grades and negative school outcomes (year fail or repetition, school expulsion). Data on school grades will be obtained from schools, provided permission from education authorities is given. This will be obtained at the end of each school year for the duration of the study, or on a quarterly basis, depending on how schools’ examinations operate in each site.
- Expectations and aspirations on education, migration and family formation [47]: Expectations on education are assessed by asking children about their expected educational outcomes (i.e. plans to continue studying, expected schooling level), and expectations of work and education after finishing high school. At follow-up, we also ask adolescents whether they searched information about future education opportunities. Migration aspirations are measured by the extent to which they wish to move outside of their current area; and marriage and family formation (marriage and children) aspirations are measured by the extent to which they wish to marry and have children before the age of 25.
- Knowledge about education: This is measured based on their level of information about employment and earning differences between educational levels.
- Negotiation skills and outcomes: This is measured with a scale that assesses the extent to which they consider themselves about to understand others’ point of view, a key part of negotiation. We also assess household task allocation as a measure of the outcome of negotiation, using the number of hours spent in household chores as an indirect measure of children’s negotiation skills that captures the extent to which they can negotiate their time distribution for household chores [23].
- Financial literacy: This is captured through questions about financial knowledge (e.g. participants’ understanding of financial concepts such as budgeting and saving); financial attitudes (attitudes and believes about money management, attitudes towards use of budgets); and financial behaviour (such as saving, borrowing and consumption behaviour, use of budget plans) [58].
- Risk preferences: This is assessed through standard questions about how much of a guaranteed amount children are willing to invest in a risky option with a clear probabilistic outcome.
- Social preferences (altruism): the extent they value the welfare of others in their decision-making, measured through a question about willingness to share a hypothetical cash transfer.

The adolescent assessment will also include heart rate variability (HRV), which is the variation between beat-to-beat intervals. HRV has consistently been shown in meta-analyses to correlate with stress [59], anxiety [60], depression [61] and self-regulation [62]. We will measure 5-min resting state HRV using either a wearable ECG device, the Polar H10 chest strap, which is a well-validated method for measuring HRV [46, 47], or when not feasible, a photoplethysmography (PPG) Bluetooth earlobe device from HeartMath (www.heartmath.com), which has also demonstrated acceptable accuracy at rest [63]. Data will be recorded using the HRV logger application, using a similar procedure as reported by [64]. Since body mass index can explain some of the variance in HRV, weight and height will also be recorded and controlled for [65]. HRV assessments were “resting state” measures, so physical activity was standardised. However, the HRV measures were taken at different times of day, depending on scheduling in schools, so this was not standardised [66].

Finally, three neuropsychological tasks, which have been culturally adapted for the three ALIVE contexts, will also be administered: the Balloon Analogue Risk Task – Youth version (BART-Y) [67], the Emotional Go No-Go Task [68] (in Colombia and South Africa) and the Delay Discounting Task [69]. The BART–Y assesses decision-making and is a computerised measure of risk-taking behaviour balancing the potential for reward versus loss. The original BART includes cash as a reward, which are converted to prizes in the BART-Y. However, several studies have demonstrated that tasks using hypothetical rewards provide valid measures of risk taking [70]. The task will therefore use a points accumulation displayed in the BART-Y, rather than cash or prizes.

The Emotional Go No-Go (EGNG) Task measures the ability to inhibit responses to emotional stimuli and will be included as a test of emotion regulation [68]. Rather than passively viewing emotions or attending to non-emotional features of the stimuli, this task directly measures the ability to approach or avoid the relevant emotional information. This task has been validated in neuroimaging studies to dissociate top-down prefrontal cognitive systems from subcortical limbic regions for both negative and positive emotions [71]. The EGNG was adapted in each country to include photographic stimuli of local adolescents displaying different emotions. The adaptation included happy, sad, fear, anger, surprise (for practice trials only) and neutral (the reference non-emotional stimulus). In each country, the emotional expressions were validated by testing recognition accuracy in approximately 60 local adolescents, before the photos were included in the task. To limit task duration, this intervention study used only the contrast between neutral, happy and sad faces because these two emotional expressions have shown the strongest association with depression and anxiety [51]. Due to poor recognition performance for the localised emotional stimuli generated in Nepal, the EGNG was not used in Nepal.

The Delay Discounting Task assesses salience evaluation and more specifically measures the decrease in the subjective value of a reward as the delay to its receipt increases. Delay discounting allows the individual to compare values between the immediate and delayed consumption of a determined commodity [52]. Such a concept plays an important role in studies related to self-control and impulsiveness in decision-making. For ALIVE, the DDT was adapted to present visual images of local currency notes. Participants do not receive a real reward for completing this task, for reasons of feasibility.

#### Outcomes instruments for caregivers

The caregiver assessment includes the positive parenting, positive involvement and corporal punishment subscales of the Alabama Parenting Questionnaire [72], the PHQ-9 [53] and additional questions covering household members, migration background, contextual stressors, adverse life events, welfare benefits, income, debt, household expenditure, aspirations and beliefs for index child/self [47], Multi-Dimensional Poverty Index, adapted for each country [30, 48, 73], and social security.

#### Assessment of costs and cost consequences of the intervention

As part of the objective to test trial procedures, we will also conduct an exploratory economic evaluation of all three newly developed interventions, in which we will examine costs and possible offsets of delivering the intervention in the three country settings. In each country, we will collect information from key ALIVE team members about resource inputs that go into the delivery of the interventions, and into training and supervision of the facilitators. Information sources that we will draw from include project administration data such as manuals, attendance and budget sheets.

An adapted version of the Client Service Receipt Inventory, developed by Beecham and Knapp [54], will also be included in the adolescent and caregiver assessments to assess use and cost of physical and mental health services and wider support and advice (e.g. with debt, career). Caregivers will report for themselves and the index adolescent participant at all follow-up assessments, while adolescent participants will also report for themselves at the 12-month post-baseline assessment.

### Procedures

#### Data collection for pilot cRCT

**Fig. 1:**
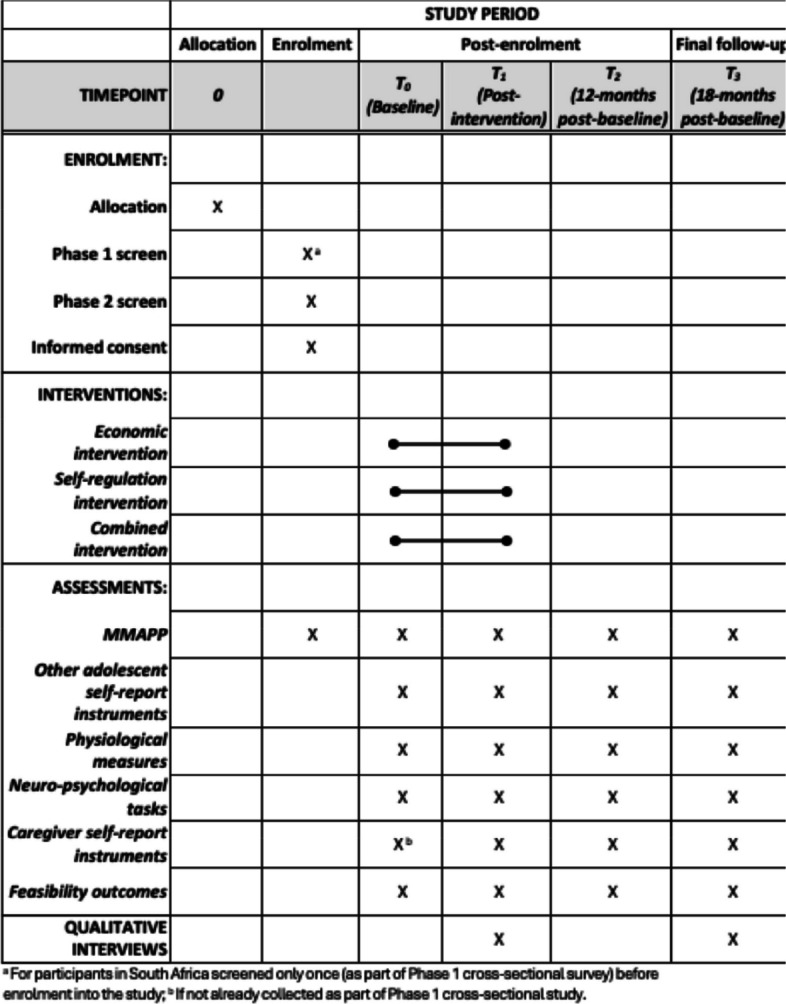
SPIRIT schedule of enrolment, interventions and assessments for participants

**Fig. 2:**
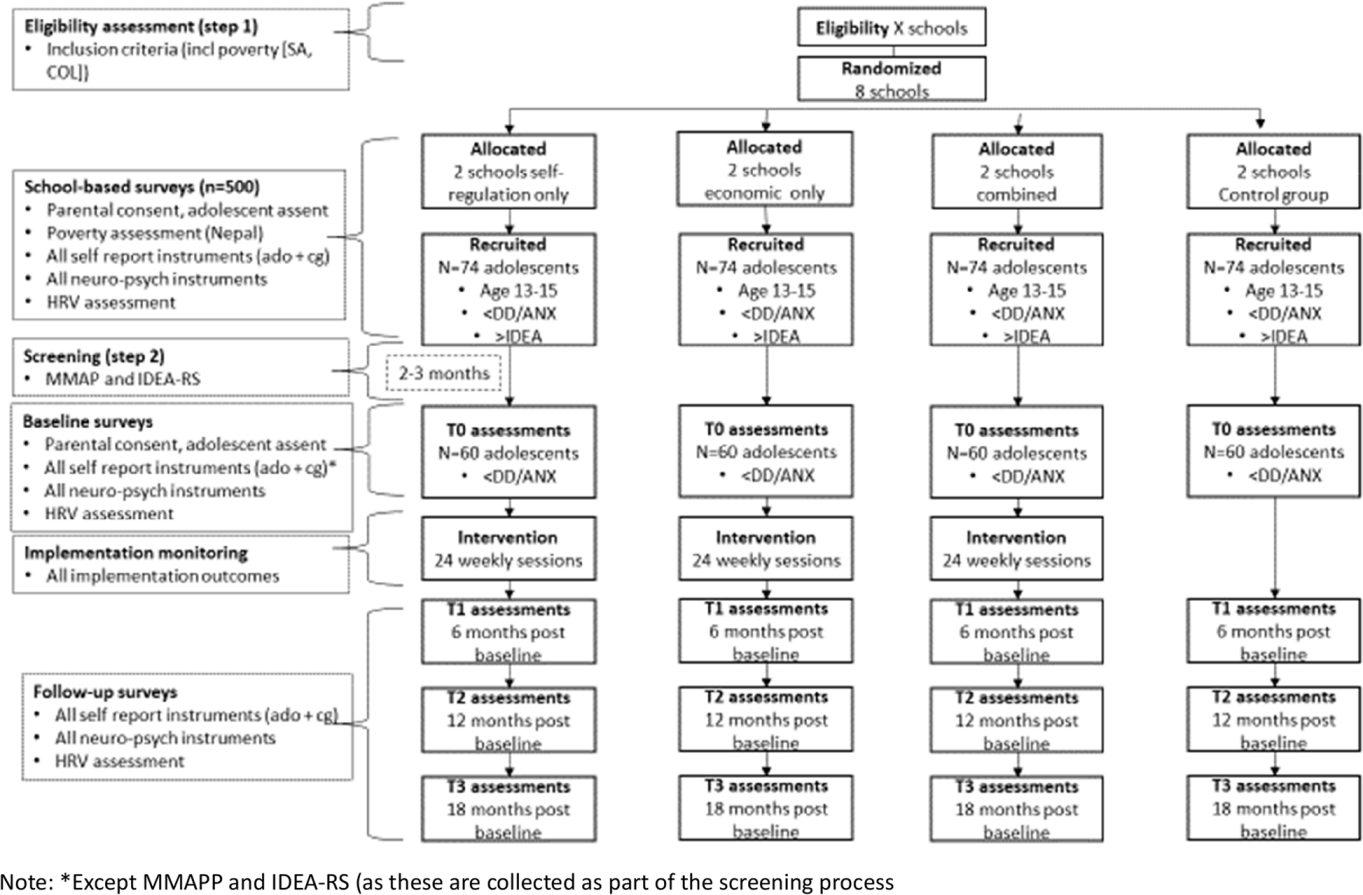
Study timeline for enrolment, intervention and assessments of participants

Refer to Fig. 1 for the SPIRIT schedule of enrolment, interventions and assessments. Adolescents and caregivers will be assessed on four occasions to assess sensitivity to change of measures and to assess a change in incidence of depression and/or anxiety, as well as all other outcomes, from baseline (T0) to post intervention (T1), 12 months post-baseline (T2), and 18 months post-baseline (T3). Baseline data collection for adolescents was ideally completed 1–4 weeks before the intervention started and initiated immediately after the screening questionnaire (MMAPP) and enrolment. If baseline data for caregivers were not already collected as part of the Phase 1 cross-sectional survey, baseline data were also collected 1–4 weeks before the intervention started. Data collection for both adolescents and caregivers at T1, T2 and T3 will be conducted within a 3-week window from the scheduled post-baseline timepoint. Please refer to Fig. 2 for an overview of the timeline for step 1 and step 2 enrolment, interventions and assessments of participants.

Data collection for adolescents takes place in a closed venue (e.g. classroom) or private room either on school premises, or within their homes, depending on participants’ preferences. First, the self-report instruments and neuropsychological tasks are administered and the physiological measures collected by research assistants. In South Africa, the self-report instruments are self-administered in small groups, with the help of the research assistants. In Colombia and Nepal, they are administered individually to each participant, though participants may be asked to self-administer several key sensitive items to avoid social desirability. The administration of the instruments for the primary caregiver takes place either on school premises, or in the participants’ homes, reverting to remote data collection only if needed. The assessments should take approximately 60–90 min (for adolescents) and 60 min (for caregivers) to complete.

Data are collected electronically using Android tablets linked to the ODK platform (https://getodk.org/); regular checks of data values are performed to ensure data quality. All data collection is conducted in Spanish (Colombia), Nepali (Nepal) and English (South Africa). In South Africa, the research is taking place in the Western Cape province, where English, Afrikaans and isiXhosa are the most frequently spoken languages. Nevertheless, previous research has shown that adolescents prefer completing questionnaires or interviews in English, even if their first language is isiXhosa or Afrikaans. For caregivers in South Africa, however, instruments are administered in English, isiXhosa or Afrikaans, depending on the caregivers’ preferences.

#### Masking

All lead investigators and research assistants (except research assistants in South Africa) will remain masked to allocation status of participants enrolled into the study. A Standard Operating Procedure (SOP) has been put in place with details on who can know what, on what scripts can be used when communicating about the study and what strategies are in place to prevent and assess unmasking. To maintain masking, separate research assistants will conduct the qualitative and quantitative assessments, research assistants will have separate offices to intervention facilitators, and the level of (un)masking will be assessed at the end of each of the four assessments.

#### Contamination and drifting

Participants will not be given information about the content of the intervention provided in the other trial arms at any point during the study. However, to assess the extent of contamination across trial arms, participants in all four arms will be asked several structured questions at each of the measurement points (excluding baseline) about the extent to which they shared information and materials about the intervention received with others in the community (intervention arms), and whether they have heard of information or materials about the intervention from others (all four arms). This information will be used descriptively to assess the level of contamination and drifting. As part of the inclusion criteria, we have included sufficient distance between schools (> 1 km) to reduce the risk of contamination.

#### Data Safety Management Board

A Data Safety Management Board (DSMB) was established prior to the start of the pilot trial for the duration of the study, with a Charter that defines its role and terms of reference. It provides oversight on adverse events (AE, including Severe Adverse Events (SAE)) and safety protocols for the trial, to determine if interim analyses should be undertaken, and to make decisions as to the continuation of the trial in case of severe adverse events. The DSMB consists of four members, one expert from each site, and an independent statistician. The DSMB meets every 3 months for the duration of the trial. Each report of an (S)AE is sent to the DSMB, who ascertains whether the SAE is a result of participating in the trial, and whether the adequate response has been taken. The DSMB has the mandate to recommend stopping the study if it is determined that the risks of the study outweigh the benefits.

#### Data collection for qualitative interviews

Trained research assistants will conduct the qualitative interviews (within 4 weeks from terminating the intervention), independent from the team conducting quantitative assessments, given that intervention allocation of families would be revealed in these interviews. Semi-structured interviews (either individual or focus group discussions) will follow semi-structured guides, with topics exploring the perceived acceptability, feasibility and impact of the ALIVE intervention in each site, including facilitators and barriers to implementing the ALIVE interventions and completing the assessments, as well as recommendations for improvements. The interviews will be conducted in Spanish (Colombia), Nepali (Nepal) and English, isiXhosa or Afrikaans (South Africa); they should last approximately 30 min. The interviews will take place in the partner organisations’ offices or participants’ homes or in another private location of their choice or remotely. The brief interviews will be audio-recorded for transcription and translation purposes.

#### Training of research assistants

All research assistants conducting the assessments and qualitative interviews have experience in working among urban poor individuals. They will receive training lasting on average 3–6 days, reviewing general interview techniques, administering the instruments, and ethical research practice emphasising responding to participant distress. Following the training, research staff will have 2 weeks of practising assessments and interview role plays until the start of the trial. Research assistants will also be trained in the Adverse Events Reporting Mechanism, which guides the process of reporting and supporting/referral in case of any adverse events (see below).

#### Compensation of respondents

Each participant will receive supermarket vouchers or tokens of a maximum value of GBP 4 (Colombia), GBP 3 (Nepal) and GBP 8 (South Africa) after each screening/assessment or interview. A small reimbursement for transportation costs will also be provided when assessments, sessions or interviews take place outside of school or place of work, or outside of school-going/working hours (to a maximum value of GBP 7 (Nepal), GBP 2 (Colombia) and GBP 10 (South Africa) per participant per round trip). Refreshments will be provided during all assessment sessions, for adolescents and caregivers.

#### Adolescent Advisory Groups

Throughout the preparation for, and implementation of, the cRCT Adolescent Advisory Groups (AAGs) that have been established in each of the three countries as part of ALIVE, will provide inputs on the content of the quantitative and qualitative research instruments, as well as on the intervention content and delivery methods. Using participatory exercises, recurrent AAG meetings will pilot-test translations of instruments to advice on concerns of acceptability (e.g. wearing HRV devices, acceptable duration of assessments) and will review and advice intervention activities (e.g. type of physical activities, use of terminology). Furthermore, AAGs will be asked advice about measures we should put in place to reduce any negative influences of taking part in the interventions and study.

### Data analysis

#### Analyses of criteria for progression to full trial

The primary analyses for this pilot cRCT concern the assessment of a priori defined criteria that will be used for decision making for progression from pilot trial to an adequately powered trial. These criteria will be scored using a “red” (study cannot progress “as-is” but will require major adaptations to content or procedure), “amber” (progression can happen, but small adaptations should be considered to improve content of procedure) and “green” scoring (progression can happen without any adaptations). The criteria are listed in Table 7 and pertain to the feasibility of randomisation, data collection, masking and retention, as well as fidelity and adherence to ALIVE interventions, (severe) adverse events and safety of the cash transfers.

**Table 7:** Criteria for progression to full trial

Criteria item	Indicator/measure	Alternative traffic light system		
		Red	Amber	Green
**Feasibility of the study procedures**				
Feasibility of randomisation	Difference in participant recruitment rate between arms	1. > 60% participants agree to be (i) re-screened to determine eligibility for the pilot, or (ii) enrolled into the pilot (when second screening for pilot does not take place) 2. > 30% difference in recruitment rate between arms	1. > 60% participants agree to be (i) re-screened to determine eligibility for the pilot, or (ii) enrolled into the pilot (when second screening for pilot does not take place) 2. 20–30% difference in recruitment rate between arms	1. > 60% participants agree to be (i) re-screened to determine eligibility for the pilot, or (ii) enrolled into the pilot (when second screening for pilot does not take place) 2. < 20% difference in recruitment rate between arms
Feasibility of masking research assistants	Self-report questions at the end of each assessment	> 50% guess correct arm	35–50% guess correct arm	< 35% guess correct arm
Feasibility of data collection	Proportion of missing items on the GAD-7 and PHQ-A items at 18 month follow-up	> 20% missing	10–20% missing	< 10% missing
Feasibility of retention	Proportion of recruited adolescents lost to follow-up (LTFU) at 18-month follow-up	> 30% LTFU	20–30% LTFU	< 20% LTFU
**Feasibility of intervention**				
Fidelity to ALIVE interventions	Average fidelity across intervention arms, based on Fidelity checklists completed for 10 − 15% of the sessions by trainer/supervisors	< 60% Fidelity	60–75% Fidelity	> 75% Fidelity
Adherence to intervention	Attendance data at each session, as reported on TeamPact	Retention of < 50% of participants through adequate completion of sessions (i.e. > 70%), in each intervention arm	50–70% retention in each intervention arm	> 70% retention in each intervention arm
Presence of (severe) adverse events	Across all active arms compared to control arm	> 20% increase	10–20% increase	< 10% increase
Safety of the cash transfer	Post session feedback from facilitators; Interviews with adolescents and caregivers; (S)AEs	> 30% participants report a negative event	10–30% participants report a negative event	< 10% participants report a negative event

#### Sensitivity to change analyses on participants outcome data

All outcome analyses will take a sensitivity to change approach. This will include descriptive analyses of the cumulative incidence of depression/anxiety (binary outcome), as well as analyses of change on other outcomes using linear mixed models. All results aim to inform a future definitive trial (event rates and effect sizes, respectively), for outcome selection and sample size calculations.

The primary comparison of interest for the adolescent in a future adequately powered trial is the difference in cumulative incidence rate of depression and anxiety throughout the study until 18 months post-baseline (i.e. proportion of adolescents per arm who score above the depression and/or anxiety cut-off at any one of the follow-up time points). We selected this measurement strategy as it takes into account the episodic nature of depression and anxiety. In addition, a point incidence rate at a single time point, such as 18 months, would miss those who developed depression or anxiety along the way but who are not suffering from either depression and/or anxiety at 18 months post baseline, and would result in lower rates, which will make achieving power for a larger trial more difficult. Exploratory analyses (e.g. based on latent class analysis) can be based on “persistent” depression or anxiety, operationalised as 2 or more time points above cut-off among the T1, T2 and T3 follow-up timepoints. For the pilot trial, we will run descriptive comparisons of cumulative incidence between the arms, while not testing for statistical differences. We hypothesise that the cumulative incidence rate will be higher in the control arm when compared to the intervention arms.

For secondary outcomes of a future adequately powered trial, we will explore the difference in change in each outcome within each trial arm from baseline to each follow-up using linear mixed effects regression models (i.e. assessment of *sensitivity to change*). No formal between group statistical testing will be conducted as this pilot cRCT is not designed to detect effects. Given the small number of clusters (or schools) per arm (*n* = 2), the models will include fixed effects of timepoint and school [74]. For each outcome, we will present the model-predicted means and corresponding 95% confidence intervals for each trial arm and timepoint. 

Following multiple imputation of missing data among participants followed-up, models will each be estimated two times for each outcome in each country. First, following an intent-to-treat principle, we will estimate models including all study participants at all timepoints and according to their randomisation allocation, regardless of attendance. Second, the same models will be estimated but only among the subset of adolescent participants who attended > 70% of the 20 intervention sessions (defined as “completers”). Where possible, models will control for variables included in the random selection of schools (district (Colombia), school size (Nepal) and language (South Africa)). The Benjamini–Hochberg correction will be applied to correct for multiple comparisons.

After analyses are conducted on each of the country-specific data sets, cross-country analyses will be conducted; this may include pooled cross-sectional analyses on the baseline data, or pooled outcome analyses assessing change over time. Models will account for clustering at school level, and random intercepts will be specified for schools and participants to reflect the hierarchical structure of the data. To account for the small number of clusters and likely non-normal distribution of outcomes, we will use a Bayesian multilevel negative binomial or Poisson regression model estimated via Markov Chain Monte Carlo. We will conduct heterogeneity analyses to assess differences in the impact of the intervention across sites, employing interaction effects in regression models on a pooled dataset of all three sites. Analyses will be conducted using Stata, version 16.

The abovementioned sensitivity-to-change analyses will provide input for the expected effect sizes that will be needed for sample size calculations for an adequately powered cRCT, if all criteria for progression to full trial are met (see above). For the same purpose, we will use the baseline data to assess the Intracluster Correlation Coefficient (ICC) due to clustering by school. Additionally, because of interest in targeting adolescents most likely to develop depression or anxiety, we will also descriptively review sensitivity to change with stratification by the IDEA-RS. A detailed statistical analyses plan for all outcomes and potential mediation will be developed, and made openly available, before the end of data collection.

#### Cost and cost consequences analyses

Cost analyses will consist of first calculating the costs of delivering the interventions in each country, both under conditions of the ALIVE study and under predicted real-world scenarios. For this, we will estimate unit costs for staff time and apply unit costs to time inputs of staff to calculate workforce costs. Similarly, we will determine market prices for other cost items such as material, travel and equipment. We will then apply unit costs and market prices to each item with the total costs of delivering the interventions in each country being aggregated. Sensitivity analysis will be conducted to reflect uncertainty of some of the parameters.

#### Qualitative analyses

Qualitative data analysis will be conducted separately in each country following procedures for inductive thematic analysis, using a qualitative analysis software (e.g. NVivo). Initial coding will be done by at least two persons with an overlap of at least 10% of the transcriptions, or until a 70% inter-rater agreement is reached. We will be using a framework analysis approach [75]. We will be combining qualitative and quantitative findings, using a convergent parallel mixed methods approach. The findings will primarily be used for revising the intervention content and delivery for a possible future effectiveness trial. Moreover, the qualitative data was used to gauge the safety of the cash transfers in the combined and economic intervention arms.

### Data management

Study information and consent forms will be kept in locked cabinets in locked offices or encrypted and password-protected computers in the case they are stored digitally. All data obtained via ODK, HRV Logger and the neuropsychological task applications will be anonymised using participant ID numbers during data collection and when exported for analysis and storage. Interviews will be pseudonymised as they are being transcribed and audio-recordings will be destroyed immediately after transcription.

Following project completion, anonymised electronic data and all metadata will be stored on King’s College London Open Research Data System (KORDS), a data repository platform which allows for long-term storage of quantitative and qualitative data; it also allows datasets to be accessible to other researchers. The data will include an embargo of 2 years, after which the quantitative data will be openly available. Qualitative data will also be stored using KORDS, but will not be publicly available, and instead shared upon request. This is due to the sensitive and context-specific nature of qualitative data.

### Dissemination

Results of the pilot cRCT will be communicated to participants and to the adolescent advisory groups in each site, as well as to other researchers through common dissemination channels. There will be no publication restrictions and links to papers published will be made available on the ALIVE website. We will use standard authorship criteria as per ICMJE.

## Discussion

The aim of the study is to test study procedures and assess the feasibility and acceptability of a new intervention combining self-regulation and economic components to prevent depression and anxiety among adolescents living in urban poverty. Given the bidirectional relationship between poverty and poor mental health, there is a need to generate evidence on the effectiveness of interventions which address both aspects simultaneously. With this study, we expect to meet criteria for progression to full trial that have been established for intervention implementation (i.e. fidelity, retention, adverse events, experience with cash transfers) and trial procedures (i.e. randomisation, masking, data collection, retention, contamination), as well as demonstrating sensitivity to change on all self-report and neuropsychological outcome measures, primarily among participants in the combined intervention arm. From this pilot trial, we will learn whether the interventions, and especially the cash transfer, had any negative impact (e.g. perceived inequity, reduced motivation), and how these can be better mitigated in a future trial. Equally we want to understand whether and how contextual factors might influence aspects of feasibility—especially given the three different study sites (e.g. levels of poverty, gender or cultural factors, across and within the Colombia, Nepal and South African samples). For any of the above findings, we will review if and what changes can be made to address any biases or improve procedures or intervention content.

On the one hand, we hypothesise that the self-regulation aspect of the intervention will provide access to a supportive peer group and environment, including caregiver support, as well as a means to build a positive self-concept by independently mastering challenging new tasks, and offer relief from the stress caused by the adversity they experience daily; all of which should promote self-regulation, as well as improve stress management, sleep and communication with caregivers, which in turn may improve hope and increase school attendance and retention, all reducing risk of developing depression or anxiety disorders. We hypothesise that the economic intervention will reduce family financial stress, increase adolescents’ sense of control and security, enhance educational opportunities, increase hope and future orientation, improve peer and family relationships, develop life skills and increase resilience to adverse environments, as well as increase caregivers’ willingness to invest in their children’s human capital and wellbeing, also reducing risk of developing depression or anxiety disorders. It may also increase the set of possible aspirations caregivers have for their children [76], which may in turn lead to larger investments in their human capital. From the adolescent perspective, financial education should empower them to make better informed decisions about the management of their finances, including the cash transfers. Similarly, negotiation training and information on education should enable the adolescents to increase the probability of strategic cooperation that may strengthen their ability to influence their parents’ decision to raise investments. This may improve education outcomes in the short- to medium-term and promote mental wellbeing among adolescents. It is thus essential to conduct a feasibility study first that combines both elements of self-regulation and anti-poverty interventions, as well as assesses the relative change between intervention arms that combine or separate these elements.

## Conclusions

More evidence is needed on the effectiveness of prevention studies for adolescents in LMICs, on the potential benefits of integrating mental health and economic intervention components, and on understanding the causal mechanisms by which complex interventions work. The current study is uniquely placed to work towards answering these questions, especially for adolescents faced with poverty in Colombia, Nepal and South Africa.

## Supplementary Information

Additional file 1: Characteristics of the trainers/supervisors (.docx).

Additional file 2: Characteristics of the facilitators (.docx).

Additional file 3: Self-report assessments for adolescents (.docx).

Additional file 4: Self-report assessments for caregivers (.docx).

## Data Availability

The datasets generated and analysed during this study will be available on the King’s Open Research Data System (KORDS). The qualitative data will be available from the corresponding author on reasonable request.

## Abbreviations

ALIVE: Improving Adolescent mentaL health by reducing the Impact of poVErty
BART-Y: The Balloon Analogue Risk Task – Youth version
cRCT: Cluster randomised controlled trial
DSMB: Data Safety Management Board
ENACT: Enhancing Assessment of Common Therapeutic factors
GAD-7: Generalised Anxiety Disorder Scale
HRV: Heart rate variability
KORDS: King’s College London Open Research Data System
LMICs: Low- and middle-income countries
MMAPP: Measurement of Mental Health among Adolescents and Young People at the Population level Tool
PHQ-A: Patient Health questionnaire – Adolescent version
SAE: Severe adverse events
TPO: Transcultural Psychosocial Organisation
W4C: Waves for Change
WeACT: Working with Children Assessment of Competencies Tool
