# Supplementary material for: Combining self-regulation and poverty reduction to prevent depression and anxiety among adolescents experiencing multi-dimensional poverty in Colombia, Nepal and South Africa: study protocol of a pilot 4-arm cluster randomised controlled trial
Source: Pilot Feasibility Stud. 2026 Jun 8;12:113. doi: 10.1186/s40814-026-01858-y (PMC13465900; doi:10.1186/s40814-026-01858-y)
Supplement: Supplementary file 1 [file 40814_2026_1858_MOESM1_ESM.docx]

Additional file 1: Characteristics of the trainers/supervisors

|  | **Colombia** | **Nepal** | **South Africa** |
| --- | --- | --- | --- |
| **Trainers (2 per country per arm)** | | | |
| Economic intervention | - 25-40 years of age | - 25-40 years of age | - 25-40 years of age |
|  | - English speaking | - English speaking | - English speaking |
|  | - Experience in conducting training on financial, cooperative or microfinance management | - Experience in conducting training on financial, cooperative or microfinance management | - Experience in conducting training on financial, cooperative or microfinance management |
|  | - Education: Technical of bachelor’s degree in accounting, finance, administration, economics or equivalent | - Education: Minimum BBA in Business Administration, BCom or BA in Business Studies, or equivalent | - Education: BComm or equivalent (or undergraduate Commerce student) |
|  | - Understanding of basic financial management skills, like budgeting | - Understanding of basic financial management skills, like budgeting | - Understanding of basic financial management skills, like budgeting |
| Self-regulation intervention | - 25-40 years of age | - 25-40 years of age | - 25-40 years of age |
|  | - English speaking | - English speaking | - English speaking |
|  | - Experience with teaching adolescents | - Experience in (similar) psychosocial trainings | - Experience in (similar) psychosocial trainings |
|  | - Technical or bachelor’s degree in psychology or equivalent | - Education: Post Graduate Diploma or bachelor’s in psychology, or equivalent | - Education: Post Graduate Diploma or bachelor’s in psychology, or equivalent |
| Combined intervention | - One of each of the above | - One of each of the above | - One of each of the above |
