# Supplementary material for: Combining self-regulation and poverty reduction to prevent depression and anxiety among adolescents experiencing multi-dimensional poverty in Colombia, Nepal and South Africa: study protocol of a pilot 4-arm cluster randomised controlled trial
Source: Pilot Feasibility Stud. 2026 Jun 8;12:113. doi: 10.1186/s40814-026-01858-y (PMC13465900; doi:10.1186/s40814-026-01858-y)
Supplement: Supplementary file 2 [file 40814_2026_1858_MOESM2_ESM.docx]

Additional file 2: Characteristics of the facilitators

|  | **Colombia** | **Nepal** | **South Africa** |
| --- | --- | --- | --- |
| Economic intervention | - 20-30 years of age | - 20-30 years of age | - 20-30 years of age - Needs to be fully bilingual (either English/ Afrikaans or English/ isiXhosa) |
|  | - Experience working with children or adolescents | - Experience working with children or adolescents | - Experience working with children or adolescents |
|  | - Good adolescents and adult-learning skills - Understanding of basic financial management skills, like budgeting | - Good adolescents and adult-learning skills - Understanding of basic financial management skills, like budgeting | - Good adolescent and adult-learning skills - Understanding of basic financial management skills, like budgeting |
|  | - Recruited from similar communities to the adolescent participants | - Recruited from similar communities to the adolescent participants | - Recruited from similar communities to the adolescent participants |
|  | - Education: intermediate degree in any discipline | - Education: intermediate degree in any discipline or bachelor’s degree not limited to psychology/ psychiatry | - Education: High school and some post-school training |
| Self-regulation intervention | - 20-30 years of age | - 20-30 years of age | - 20-30 years of age |
|  | - 2-3 years of experience with psychosocial interventions with adolescents - Experience working with children or adolescents | - Intermediate-level nurse - Experience working with children or adolescents | - 2-3 years of experience with psychosocial interventions with adolescents (Alumni W4C Coaches) - Experience working with children or adolescents |
|  | - Good adolescents and adult-learning skills | - Good adolescents and adult-learning skills | - Good adolescents and adult-learning skills |
|  | - Recruited from similar communities to the adolescent participants | - Recruited from similar communities to the adolescent participants | - Recruited from similar communities to the adolescent participants |
|  | - Competency in physical activity | - One of the two facilitators will have the competency to run martial arts-based activities | - Competency in physical activity (surfing), for S-R and Combined interventions only |
|  | - Education: intermediate degree in any discipline | - Education: intermediate degree in nursing or bachelor’s degree not limited to psychology/ psychiatry | - Education: High school and some post-school training |
| Combined intervention | - 20-30 years of age | - 20-30 years of age | - 20-30 years of age |
|  | - Experience working with children or adolescents | - Intermediate-level nurse for psychosocial part of the intervention or bachelor’s degree not limited to psychology/ psychiatry - No experience with psychosocial/economic interventions - Experience working with children/adolescents | - 2-3 years of experience with psychosocial interventions with adolescents (Alumni W4C Coaches)/ - Experience working with children or adolescents |
|  | - Good adolescents and adult-learning skills - Understanding of basic financial management skills, like budgeting | - Good adolescents and adult-learning skills - Understanding of basic financial management skills, like budgeting | - Good adolescents and adult-learning skills - Understanding of basic financial management skills, like budgeting |
|  | - Recruited from similar communities to the adolescent participants | - Recruited from similar communities to the adolescent participants (for nurse) | - Recruited from similar communities to the adolescent participants |
|  | - Competency in physical activity | - One of the two facilitators will have the competency to run martial arts-based activities | - Competency in physical activity (surfing) |
|  | - Education: intermediate degree in any discipline | - Education: intermediate degree in any discipline or bachelor’s degree not limited to psychology/ psychiatry | - Education: High school and some post-school training |
