# Supplementary material for: Combining self-regulation and poverty reduction to prevent depression and anxiety among adolescents experiencing multi-dimensional poverty in Colombia, Nepal and South Africa: study protocol of a pilot 4-arm cluster randomised controlled trial
Source: Pilot Feasibility Stud. 2026 Jun 8;12:113. doi: 10.1186/s40814-026-01858-y (PMC13465900; doi:10.1186/s40814-026-01858-y)
Supplement: Supplementary file 3 [file 40814_2026_1858_MOESM3_ESM.docx]

**Adolescent self-report questionnaire (version 5 – 9 October 2024)**

| A. Introduction | | | | |
| --- | --- | --- | --- | --- |
| Instructions: “*Please answer each question*” | | | | |
| A1 | Date | 🖎 |  | date |
| A2 | Time | 🖎 |  | starttime |
| A3 | Which timepoint is this? | Baseline | 1 | timepoint |
|  |  | Post-intervention | 2 |  |
|  |  | 12-month post baseline | 3 |  |
|  |  | 18-month post baseline | 4 |  |
| A4 | Which fieldworker is completing this questionnaire | Johana Gallego [Col] | 11 | fw |
|  |  | Patricia Muñoz [Col] | 12 |  |
|  |  | Ysandra Tibisay Romero [Col] | 13 |  |
|  |  | Angélica Rojas [Col] | 14 |  |
|  |  | Shirley González [Col] | 15 |  |
|  |  | Claudia Bello [Col] | 16 |  |
|  |  | Mirna Mendez [Col] | 17 |  |
|  |  | Henis Molina [Col] | 18 |  |
|  |  | Aditi Ghimire [Ne] | 20 |  |
|  |  | Anushka Thapa [Ne] | 21 |  |
|  |  | Dipa Giri [Ne] | 22 |  |
|  |  | Neeru Yadav [Ne] | 23 |  |
|  |  | Bibek Prajuli [Ne] | 24 |  |
|  |  | Jushan Shrestha [Ne] | 25 |  |
|  |  | Dikshya Chand [Ne] | 26 |  |
|  |  | Sabina Karki [Ne] | 27 |  |
|  |  | Sabitra Dahal [Ne] | 28 |  |
|  |  | Sushan Ghimire [Ne] | 29 |  |
|  |  | Subina Shrestha [Ne] | 30 |  |
|  |  | Megan Pool [SA] | 31 |  |
|  |  | Xola Jiyana [SA] | 32 |  |
|  |  | Tarshlyn Herandien [SA] | 35 |  |
|  |  | Zoleka Maqina [SA] | 36 |  |
|  |  | Bulelani Velaphi [SA] | 37 |  |
|  |  | Zimasa Ranyana [SA] | 38 |  |
|  |  | Iona Gutuza [SA] | 39 |  |
|  |  | Nontobeko Mdudu [SA] | 40 |  |
|  |  | Thozama H-Kasozi [SA] | 41 |  |
|  |  | Veliswa Cebisa [SA] | 42 |  |
|  |  | Azola Sibonda [SA] | 43 |  |
|  |  | Noma Moshani [SA] | 44 |  |
|  |  | Other [Answer A5] | 77 |  |
| A5 | What other fieldworker? | 🖎 |  | fwo |
| A6 | Please enter the participants PID | 🖎 | | id |
| A7 | Please enter the participant’s PID again | 🖎 | | id2 |
| A8 | Correct 4-digit PID |  | | id_final |
| A9 | Full PID for cross-country analysis |  | | pid |
| A10 | Where is the assessment conducted? | At home | 1 | location |
|  |  | At school | 2 |  |
|  |  | On the phone | 3 |  |
|  |  | Other location [Answer A11] | 77 |  |
| A11 | Please specify other | 🖎 | | locationo |
| A12 | [Baseline only]  What is the name of the school? | C. Santa Barbara [Col] | 1 | school |
|  |  | C. La Arabia [Col] | 2 |  |
|  |  | C. La Estancia [Col] | 3 |  |
|  |  | C. Rogelio Salmona [Col] | 4 |  |
|  |  | C. San Jose de Usme [Col] | 5 |  |
|  |  | C. Chuniza Fámaco [Col] | 6 |  |
|  |  | C. Gabriel Garcia Marquez [Col] | 7 |  |
|  |  | C. Fabio Lozano Simonellli [Col] | 8 |  |
|  |  | Budhanilkantha [Ne] | 4 |  |
|  |  | Dhumra Baraha [Ne] | 5 |  |
|  |  | Gram Shikshya [Ne] | 6 |  |
|  |  | Jana Uddhar [Ne] | 7 |  |
|  |  | Mahankal Janajagriti [Ne] | 8 |  |
|  |  | Naulin [Ne] | 9 |  |
|  |  | Pashupati Mitra [Ne] | 10 |  |
|  |  | Shree Ganesh [Ne] | 11 |  |
|  |  | Budhanilkantha [Ne] | 12 |  |
|  |  | Dhumra Baraha [Ne] | 13 |  |
|  |  | Gram Shikshya [Ne] | 14 |  |
|  |  | Jana Uddhar [Ne] | 15 |  |
|  |  | Mahankal Janajagriti [Ne] | 16 |  |
|  |  | Centre of Science and Technology [SA] | 17 |  |
|  |  | Esangweni Secondary School [SA] | 18 |  |
|  |  | Houtbaai Sekonder [SA] | 19 |  |
|  |  | Joe Slovo Secondary School [SA] | 20 |  |
|  |  | Kwamfundo Secondary School [SA] | 21 |  |
|  |  | Paramount High School [SA] | 22 |  |
|  |  | Silikamva High School [SA] | 23 |  |
|  |  | Usasazo Secondary School [SA] | 24 |  |

| **B. SCREENING (Baseline only)**  **Measurement of Mental Health among Adolescents at the Population level (MMAPP)**  (Carvajal-Velez et al., 2023) | | | | | | |
| --- | --- | --- | --- | --- | --- | --- |
| Instructions:  “*Every question is important, please try your best to respond each of them. If you do not want to answer a question, please let us know. Remember that all your answers are confidential.*  *During the past two weeks, how often..*” | | | | | | |
| B1 | Have you been feeling very sad or depressed? | Never | 0 | screenmmapp_1 | |  |
|  |  | Sometimes | 1 |  |  |  |
|  |  | Often | 2 |  |  |  |
|  |  | Always | 3 |  |  |  |
|  |  | Prefer not to respond | 99 |  |  |  |
| B2 | Have you felt easily annoyed or irritable/irritated at small things? | Never | 0 | screenmmapp_2 | |  |
|  |  | Sometimes | 1 |  |  |  |
|  |  | Often | 2 |  |  |  |
|  |  | Always | 3 |  |  |  |
|  |  | Prefer not to respond | 99 |  |  |  |
| B3 | Have you not enjoyed doing things you used to enjoy (like playing sports, singing, and dancing, spending time with friends, watching videos)? | Never | 0 | screenmmapp_3 | |  |
|  |  | Sometimes | 1 |  |  |  |
|  |  | Often | 2 |  |  |  |
|  |  | Always | 3 |  |  |  |
|  |  | Prefer not to respond | 99 |  |  |  |
| B4 | Have you felt hopeless about the future? | Never | 0 | screenmmapp_4 | |  |
|  |  | Sometimes | 1 |  |  |  |
|  |  | Often | 2 |  |  |  |
|  |  | Always | 3 |  |  |  |
|  |  | Prefer not to respond | 99 |  |  |  |
| B5 | Have you felt nervous or anxious? | Never | 0 | screenmmapp_5 | |  |
|  |  | Sometimes | 1 |  |  |  |
|  |  | Often | 2 |  |  |  |
|  |  | Always | 3 |  |  |  |
|  |  | Prefer not to respond | 99 |  |  |  |
| B6 | Have you worried you can’t do anything right or are doing things poorly? | Never | 0 | screenmmapp_6 | |  |
|  |  | Sometimes | 1 |  |  |  |
|  |  | Often | 2 |  |  |  |
|  |  | Always | 3 |  |  |  |
|  |  | Prefer not to respond | 99 |  |  |  |
| B7 | Have you worried about what others think about you? | Never | 0 | screenmmapp_7 | |  |
|  |  | Sometimes | 1 |  |  |  |
|  |  | Often | 2 |  |  |  |
|  |  | Always | 3 |  |  |  |
|  |  | Prefer not to respond | 99 |  |  |  |
| B8 | Have you worried something bad will happen to you or your family? | Never | 0 | screenmmapp_8 | |  |
|  |  | Sometimes | 1 |  |  |  |
|  |  | Often | 2 |  |  |  |
|  |  | Always | 3 |  |  |  |
|  |  | Prefer not to respond | 99 |  |  |  |
| B9 | Have you worried too much about many different things at the same time (worried about school work, exams, household issues, your community and your future)? | Never | 0 | screenmmapp_9 | |  |
|  |  | Sometimes | 1 |  |  |  |
|  |  | Often | 2 |  |  |  |
|  |  | Always | 3 |  |  |  |
|  |  | Prefer not to respond | 99 |  |  |  |
| B10 | Have you felt unable to stop or control your worries? | Never | 0 | screenmmapp_10 | |  |
|  |  | Sometimes | 1 |  |  |  |
|  |  | Often | 2 |  |  |  |
|  |  | Always | 3 |  |  |  |
|  |  | Prefer not to respond | 99 |  |  |  |
| B11 | Have you not wanted to eat even when food is available or eaten too much? | Never | 0 | screenmmapp_11 | |  |
|  |  | Sometimes | 1 |  |  |  |
|  |  | Often | 2 |  |  |  |
|  |  | Always | 3 |  |  |  |
|  |  | Prefer not to respond | 99 |  |  |  |
| B12 | Have you had difficulty falling asleep? | Never | 0 | screenmmapp_12 | |  |
|  |  | Sometimes | 1 |  |  |  |
|  |  | Often | 2 |  |  |  |
|  |  | Always | 3 |  |  |  |
|  |  | Prefer not to respond | 99 |  |  |  |
| B13 | Have you been waking up during the night? | Never | 0 | screenmmapp_13 | |  |
|  |  | Sometimes | 1 |  |  |  |
|  |  | Often | 2 |  |  |  |
|  |  | Always | 3 |  |  |  |
|  |  | Prefer not to respond | 99 |  |  |  |
| B14 | Have you been sleeping too much? | Never | 0 | screenmmapp_14 | |  |
|  |  | Sometimes | 1 |  |  |  |
|  |  | Often | 2 |  |  |  |
|  |  | Always | 3 |  |  |  |
|  |  | Prefer not to respond | 99 |  |  |  |
| B15 | Have you felt you got tired easily or did not have energy to do daily activities? | Never | 0 | screenmmapp_15 | |  |
|  |  | Sometimes | 1 |  |  |  |
|  |  | Often | 2 |  |  |  |
|  |  | Always | 3 |  |  |  |
|  |  | Prefer not to respond | 99 |  |  |  |
| B16 | Have you had trouble concentrating on things, such as doing homework, household chores, or other activities (such as watching videos or using social media: WhatsApp/Instagram)? | Never | 0 | screenmmapp_16 | |  |
|  |  | Sometimes | 1 |  |  |  |
|  |  | Often | 2 |  |  |  |
|  |  | Always | 3 |  |  |  |
|  |  | Prefer not to respond | 99 |  |  |  |
| B17 | Have you felt lonely? | Never | 0 | screenmmapp_17 | |  |
|  |  | Sometimes | 1 |  |  |  |
|  |  | Often | 2 |  |  |  |
|  |  | Always | 3 |  |  |  |
|  |  | Prefer not to respond | 99 |  |  |  |
| B18 | Have you felt like a failure/disappointment or like you have let yourself or your family down? | Never | 0 | screenmmapp_18 | |  |
|  |  | Sometimes | 1 |  |  |  |
|  |  | Often | 2 |  |  |  |
|  |  | Always | 3 |  |  |  |
|  |  | Prefer not to respond | 99 |  |  |  |
| B19 | Have you had thoughts that you would rather be dead or thoughts of hurting yourself? | Never [Skip B2 section] | 0 | screenmmapp_19 | |  |
|  |  | Sometimes | 1 |  |  |  |
|  |  | Often | 2 |  |  |  |
|  |  | Always | 3 |  |  |  |
|  |  | Prefer not to respond | 99 |  |  |  |
| B20 | Have other people said that you have been moving your body more slowly than usual (for example, walking slower than before)? | Never | 0 | screenmmapp_20 | |  |
|  |  | Sometimes | 1 |  |  |  |
|  |  | Often | 2 |  |  |  |
|  |  | Always | 3 |  |  |  |
|  |  | Prefer not to respond | 99 |  |  |  |
| B21 | Have others said that you are restless or that you can’t sit still (e.g., fidgeting with objects like pens, pacing up and down)? | Never | 0 | screenmmapp_21 | |  |
|  |  | Sometimes | 1 |  |  |  |
|  |  | Often | 2 |  |  |  |
|  |  | Always | 3 |  |  |  |
|  |  | Prefer not to respond | 99 |  |  |  |
| B22 | Have you felt that it was difficult to breathe? | Never | 0 | screenmmapp_22 | |  |
|  |  | Sometimes | 1 |  |  |  |
|  |  | Often | 2 |  |  |  |
|  |  | Always | 3 |  |  |  |
|  |  | Prefer not to respond | 99 |  |  |  |
| B23 | Have you felt dizziness or faint? | Never | 0 | screenmmapp_23 | |  |
|  |  | Sometimes | 1 |  |  |  |
|  |  | Often | 2 |  |  |  |
|  |  | Always | 3 |  |  |  |
|  |  | Prefer not to respond | 99 |  |  |  |
| B24 | Have you gotten suddenly scared for no reason or without knowing what made you scared? | Never | 0 | screenmmapp_24 | |  |
|  |  | Sometimes | 1 |  |  |  |
|  |  | Often | 2 |  |  |  |
|  |  | Always | 3 |  |  |  |
|  |  | Prefer not to respond | 99 |  |  |  |
| B25 | Have you had difficulty relaxing or difficulty feeling calm? | Never | 0 | screenmmapp_25 | |  |
|  |  | Sometimes | 1 |  |  |  |
|  |  | Often | 2 |  |  |  |
|  |  | Always | 3 |  |  |  |
|  |  | Prefer not to respond | 99 |  |  |  |
| B26 | Have you felt like your heart was pounding or beating too fast? (Even when not exercising) | Never | 0 | screenmmapp_26 | |  |
|  |  | Sometimes | 1 |  |  |  |
|  |  | Often | 2 |  |  |  |
|  |  | Always | 3 |  |  |  |
|  |  | Prefer not to respond | 99 |  |  |  |
| B27 | Have you had headaches or muscle tension/pain? | Never | 0 | screenmmapp_27 | |  |
|  |  | Sometimes | 1 |  |  |  |
|  |  | Often | 2 |  |  |  |
|  |  | Always | 3 |  |  |  |
|  |  | Prefer not to respond | 99 |  |  |  |
| B28 | Have you had bad dreams or nightmares? | Never | 0 | screenmmapp_28 | |  |
|  |  | Sometimes | 1 |  |  |  |
|  |  | Often | 2 |  |  |  |
|  |  | Always | 3 |  |  |  |
|  |  | Prefer not to respond | 99 |  |  |  |
| B29 | Total depression score on MMAPP | 🖎 | | | screenmmapp_totdep | |
| B30 | Total anxiety score on MMAPP | 🖎 | | | screenmmapp_totanx | |
| B31 | Total depression score on PHQ-A | 🖎 | | | screenmmapp_totalphqa | |
| B32 | Total anxiety score on GAD-7 | 🖎 | | | screenmmapp_totalgad | |
| B33 | Screens positive for depression on PHQ-A (not eligible) | No | 0 | screen_depressed | |  |
|  |  | Yes | 1 |  |  |  |
| B34 | Screens positive for anxiety on GAD-7 (not eligible) | No | 0 | screen_anxious | |  |
|  |  | Yes | 1 |  |  |  |

| **B2. SCREENING: Suicide risk [Colombia only]** | | | | |
| --- | --- | --- | --- | --- |
| B2.1 | Have you ever thought of killing yourself in the past few weeks? | No | 0 | screenmmapp_19a |
|  |  | Yes [Answer B2.3] | 1 |  |
|  |  | Prefer not to respond | 99 |  |
| B2.3 | Have you ever attempted to kill yourself before? | No | 0 | screenmmapp_19c |
|  |  | Yes | 1 |  |
|  |  | Prefer not to respond | 99 |  |
| B2.3 | Have you thought about how you would do this? | No | 0 | screenmmapp_19d |
|  |  | Yes | 1 |  |
|  |  | Prefer not to respond | 99 |  |

| **B2. SCREENING: Suicide risk [Nepal only]** | | | | |
| --- | --- | --- | --- | --- |
| B2.1 | Have you felt that life is not worth living? | No | 0 | screenmmapp_19e |
|  |  | Yes | 1 |  |
|  |  | Prefer not to respond | 99 |  |
| B2.2 | Have you ever thought of harming yourself? | No [Skip to section end] | 0 | screenmmapp_19a |
|  |  | Yes [Answer B2.3] | 1 |  |
|  |  | Prefer not to respond | 99 |  |
| B2.3 | Have you ever hurt/harmed yourself before? | No [Go to B2.6] | 0 | screenmmapp_19c |
|  |  | Yes [Answer B2.4] | 1 |  |
|  |  | Prefer not to respond | 99 |  |
| B2.4 | Have you attempted this in the past three months? | No | 0 | screenmmapp_19c_3m |
|  |  | Yes [Answer B2.5] | 1 |  |
|  |  | Prefer not to respond | 99 |  |
| B2.5 | Have you attempted this in the past month? | No | 0 | screenmmapp_19c_1m |
|  |  | Yes [Skip to section end] | 1 |  |
|  |  | Prefer not to respond | 99 |  |
| B2.6 | Do you feel that you want to harm yourself right now? | No | 0 | screenmmapp_19b |
|  |  | Yes | 1 |  |
|  |  | Prefer not to respond | 99 |  |

| **B2. SCREENING: Suicide risk [South Africa only]** | | | | |
| --- | --- | --- | --- | --- |
| B2.1 | Have you ever thought of killing yourself in the past few weeks? | No | 0 | screenmmapp_19a |
|  |  | Yes [Answer B2.2] | 1 |  |
|  |  | Prefer not to respond | 99 |  |
| B2.2 | Do you feel that you want to kill yourself right now? | No | 0 | screenmmapp_19b |
|  |  | Yes | 1 |  |
|  |  | Prefer not to respond | 99 |  |
| B2.3 | Have you ever attempted to kill yourself before? | No | 0 | screenmmapp_19c |
|  |  | Yes | 1 |  |
|  |  | Prefer not to respond | 99 |  |
| B2.4 | [If respond yes to B2.1 or B2.2]  You have shared that you are thinking of killing yourself. Have you thought about how you would do this? | No | 0 | screenmmapp_19d |
|  |  | Yes | 1 |  |
|  |  | Prefer not to respond | 99 |  |

| C. Demographics | | | | | |
| --- | --- | --- | --- | --- | --- |
| Instructions: “*Every question is important, please try your best to respond each of them. If you do not want to answer a question, please let us know. Remember that all your answers are confidential.*” | | | | | |
| C1 | How old are you? | 🖎 | | age |  |
| C2 | *[Baseline only]* In what month and year were you born? | 🖎 | | dob |  |
| C3 | What is your preferred gender identity? | Male | 0 | gender |  |
|  |  | Female | 1 |  |  |
|  |  | Non-binary | 2 |  |  |
|  |  | Other [Answer C4] | 77 |  |  |
|  |  | Prefer not to respond | 99 |  |  |
| C4 | Please specify what other gender | 🖎 |  | gendero |  |
| C5 | *[Baseline only]* What is your ethnicity/population group (caste etc.)? | None | 0 | ethnicity |  |
|  |  | Gypsy or Roma [Col] | 1 |  |  |
|  |  | Indigenous [Col] | 2 |  |  |
|  |  | Negro, Mulatto, Afro-descendant, Afro-Colombian [Col] | 3 |  |  |
|  |  | Palenquero of San Basilio [Col] | 4 |  |  |
|  |  | Raizal of the Archipelago of San Andres, Providencia and Santa Catalina [Col] | 5 |  |  |
|  |  | Brahmin or Chhetri [Ne] | 6 |  |  |
|  |  | Dalit [Ne] | 7 |  |  |
|  |  | Janajati [Ne] | 8 |  |  |
|  |  | Madhesi [Ne] | 9 |  |  |
|  |  | Muslim [Ne] | 10 |  |  |
|  |  | Black [SA] | 11 |  |  |
|  |  | Coloured [SA] | 12 |  |  |
|  |  | Indian [SA] | 13 |  |  |
|  |  | White [SA] | 14 |  |  |
|  |  | Other | 77 |  |  |
|  |  | Don't know | 88 |  |  |
|  |  | Prefer not to respond | 99 |  |  |
| C6 | Please specify what other ethnicity | 🖎 |  | ethnicityo |  |
| C7 | *[South Africa and baseline only]* What is your preferred spoken language? | English | 3 | language |  |
|  |  | isiXhosa | 4 |  |  |
|  |  | Afrikaans | 5 |  |  |
|  |  | Other [Answer C8] | 77 |  |  |
|  |  | Prefer not to respond | 99 |  |  |
| C8 | Please specify what other language | 🖎 |  | languageo |  |
| C8.2 | *[Follow-up only]* Have you moved since the last survey? | No | 0 | moved |  |
|  |  | Yes [Respond to C9-C13, C15] | 1 |  |  |
|  |  | Prefer not to respond | 99 |  |  |
| C9 | Where do you live currently? What city? | Bogota [Col] [Answer C11] | 1 | res_city |  |
|  |  | Budhanilkantha Municipality [Ne] | 2 |  |  |
|  |  | Kathmandu Metropolitan City [Ne] | 3 |  |  |
|  |  | Gokarneshwor Municipality [Ne] | 4 |  |  |
|  |  | Western Cape [SA] | 5 |  |  |
|  |  | Other [Answer C10] | 77 |  |  |
|  |  | Prefer not to respond | 99 |  |  |
| C10 | What other city/municipality/province? | 🖎 | | res_cityo |  |
| C11 | *[Colombia only]* What district? | Usme | 1 | res_district |  |
|  |  | Ciudad Bolívar | 2 |  |  |
|  |  | Prefer not to respond | 99 |  |  |
|  |  | Other [Answer C12] | 77 |  |  |
| C12 | What other district | 🖎 |  | res_districto |  |
| C13 | *[Nepal and South Africa only]* What neighbourhood/tol/suburb? | 🖎 | | res_nb |  |
| C14 | *[Baseline only]* How long have you lived there? [Enter 0 if less than a year] | 🖎 years | | res_years |  |
|  |  | Lived there my whole life  [🡪 Go to next section] | 66 |  |  |
| C15 | *[If less than a year]* For how many months have you lived there? | 🖎 months | | res_months |  |
| C16 | *[Baseline only]* Where did you live before that? What city? | 🖎 |  | city_before |  |
| C17 | *[Baseline only]* What neighbourhood? | 🖎 |  | nb_before |  |

| D. Education | | | | |
| --- | --- | --- | --- | --- |
| Instructions:  *"Today, I'm going to ask you some questions about school, your educational aspirations and career. Every question is important, please try your best to respond each of them. If you do not want to answer a question, please let us know. Remember that all your answers are confidential.”* | | | | |
| D1 | [When not school-based assessment] Are you currently enrolled in school? | No [Answer D2, then go to D14] | 1 | enrolled |
|  |  | Yes [Go to D4] | 2 |  |
|  |  | Prefer not to respond [Go to D14] | 99 |  |
| D2 | [When not school-based recruitment]  What are the main reasons you are not enrolled in school?  *Enumerator note: Multiple answers allowed.* | My family could not afford it, didn’t have the money/ high educational costs | 1 | notenrolled_why |
|  |  | I want/need to look for a job | 2 |  |
|  |  | I have a job/have to work | 3 |  |
|  |  | I am pregnant/have baby | 4 |  |
|  |  | I am needed to help at home | 5 |  |
|  |  | I am unwell/sick/disabled | 6 |  |
|  |  | I have had bad grades | 7 |  |
|  |  | I was expelled from school | 8 |  |
|  |  | I believe I have completed enough schooling | 9 |  |
|  |  | I have to take care of housework and/or care for children and other household members (elderly, disabled, siblings etc.) | 10 |  |
|  |  | I got married or formed a couple | 11 |  |
|  |  | Classes were full at school and I didn’t get a place | 12 |  |
|  |  | I need special education | 13 |  |
|  |  | I got bullied by peers so I dropped out | 14 |  |
|  |  | I got bullied by teachers so I dropped out | 15 |  |
|  |  | I experienced challenges that affected my focus | 16 |  |
|  |  | I experienced peer pressure (e.g., gangs pressuring me to drop out) | 17 |  |
|  |  | Drug use stopped me from going to school | 18 |  |
|  |  | I’m older than the schooling age/older in age | 19 |  |
|  |  | Other reason [Answer D3] | 77 |  |
|  |  | Prefer not to respond | 99 |  |
| D3 | What is the other reason? | 🖎 |  | notenrolled_whyo |
| D4 | What grade are you currently enrolled in? | Grade 3 | 3 | grade |
|  |  | Grade 4 | 4 |  |
|  |  | Grade 5 | 5 |  |
|  |  | Grade 6 | 6 |  |
|  |  | Grade 7 | 7 |  |
|  |  | Grade 8 | 8 |  |
|  |  | Grade 9 | 9 |  |
|  |  | Grade 10 | 10 |  |
|  |  | Grade 11 | 11 |  |
|  |  | Grade 12 | 12 |  |
|  |  | Prefer not to respond | 99 |  |
| D5 | *[Post-intervention follow-up only]*  In the last four full weeks of school, how often did you skip a whole day of school? | Never | 0 | skip_day |
|  |  | One or two times | 1 |  |
|  |  | Three of four times | 2 |  |
|  |  | Five or more times | 3 |  |
|  |  | Prefer not to respond | 99 |  |
| D6 | *[Post-intervention follow-up only]*  In the last four full weeks of school, how often did you skip some classes? | Never | 0 | skip_classes |
|  |  | One or two times | 1 |  |
|  |  | Three of four times | 2 |  |
|  |  | Five or more times | 3 |  |
|  |  | Prefer not to respond | 99 |  |
| D7 | *[Post-intervention follow-up only]*  In the last four full weeks of school, how often did you arrive late for school? | Never | 0 | late_school |
|  |  | One or two times | 1 |  |
|  |  | Three of four times | 2 |  |
|  |  | Five or more times | 3 |  |
|  |  | Prefer not to respond | 99 |  |
| D8 | *[If score 1-3 on D5 or D6]*  In the last four full weeks of school, why did you miss school? (Select all that apply) | I was bored | 1 | skip_why |
|  |  | I was suspended for something (e.g. violence, aggression, use of drugs, drug dealing) | 2 |  |
|  |  | I was pregnant | 3 |  |
|  |  | I could not reach school because of transportation problems | 4 |  |
|  |  | I had to take care of a family member | 5 |  |
|  |  | I had to help with work at home, the family business, or on the family land | 6 |  |
|  |  | I had to get work to bring money home | 7 |  |
|  |  | I was sick | 8 |  |
|  |  | I had my menstrual period | 9 |  |
|  |  | I did not feel safe at school or on the way to or from school | 10 |  |
|  |  | I could not pay school fees | 11 |  |
|  |  | I did not want to get punished by the teacher because of incomplete tasks | 12 |  |
|  |  | School was closed because of a natural disaster (e.g. flood, earthquake) | 13 |  |
|  |  | There were strikes or protests by general people/political parties | 14 |  |
|  |  | I had to participate in some other school-based program or extra-curricular activities | 15 |  |
|  |  | Other reason [Answer D9] | 77 |  |
|  |  | Don’t know | 88 |  |
|  |  | Prefer not to respond | 99 |  |
| D9 | Other reason for missing school | 🖎 | | skip_whyo |
| D10 | *[Post-intervention follow-up only]*  In the last four full weeks of school, on how many days did you work for pay? | 0 days | 0 | daysworked |
|  |  | 1 day | 1 |  |
|  |  | 2 days | 2 |  |
|  |  | 3 days | 3 |  |
|  |  | 4 days | 4 |  |
|  |  | 5 or more days | 5 |  |
|  |  | Don't know | 88 |  |
|  |  | Prefer not to respond | 99 |  |
| D11 | *[Post-intervention follow-up only]*  In the last four full weeks of school, on how many days did you exercise or practice a sport (e.g. running, cycling, aerobics, soccer)? | 0 days | 0 | dayssport |
|  |  | 1 day | 1 |  |
|  |  | 2 days | 2 |  |
|  |  | 3 days | 3 |  |
|  |  | 4 days | 4 |  |
|  |  | 5 or more days | 5 |  |
|  |  | Don't know | 88 |  |
|  |  | Prefer not to respond | 99 |  |
| D12 | In the last week of school, how many hours do you spend on average per day on your schoolwork and studying after your classes are done? (Please include individual work at home, staying after school, or any other non-regular academic activities) | 🖎 hours | | time_homework |
|  |  | Don't know | 88 |  |
|  |  | Prefer not to respond | 99 |  |
| D13 | Do you plan to continue being enrolled in your schooling next year (e.g. grade 6 to grade 7, SLC to +2 etc.) | No | 0 | continue_school |
|  |  | Yes | 1 |  |
|  |  | Don’t know | 88 |  |
|  |  | Not applicable | 66 |  |
|  |  | Prefer not to respond | 99 |  |
| D14 | *[Baseline only]*  Have you ever failed a full academic year? | No | 0 | failed_year |
|  |  | Yes | 1 |  |
|  |  | Don't know | 88 |  |
|  |  | Prefer not to respond | 99 |  |
| D15 | *[Baseline only]*  Have you repeated an entire year of schooling (entire grade level)? | No | 0 | repeated_year |
|  |  | Yes | 1 |  |
|  |  | Don't know | 88 |  |
|  |  | Prefer not to respond | 99 |  |
| D16 | *[Baseline only]*  Have you ever dropped out of school for any reason other than failing an academic year? | No | 0 | droppedout |
|  |  | Yes | 1 |  |
|  |  | Don't know | 88 |  |
|  |  | Prefer not to respond | 99 |  |
| D17 | *[Baseline only]*  Have you ever been kicked out of school? | No | 0 | kickedout |
|  |  | Yes | 1 |  |
|  |  | Don't know | 88 |  |
|  |  | Prefer not to respond | 99 |  |
| D18 | *[12-month-post baseline only]*  Have you failed a full academic year in the past 6 months? | No | 0 | failed_year_6m |
|  |  | Yes | 1 |  |
|  |  | Don't know | 88 |  |
|  |  | Prefer not to respond | 99 |  |
| D19 | *[12-month-post baseline only]*  Have you repeated an entire year of schooling (entire grade level) in the past 6 months? | No | 0 | repeated_year_6m |
|  |  | Yes | 1 |  |
|  |  | Don't know | 88 |  |
|  |  | Prefer not to respond | 99 |  |
| D20 | *[Follow-up only]*  Have you dropped out of school for any reason other than failing an academic year in the past 6 months? | No | 0 | droppedout_6m |
|  |  | Yes | 1 |  |
|  |  | Don't know | 88 |  |
|  |  | Prefer not to respond | 99 |  |
| D21 | *[Follow-up only]*  Have you been kicked out of school in the past 6 months? | No | 0 | kickedout_6m |
|  |  | Yes | 1 |  |
|  |  | Don't know | 88 |  |
|  |  | Prefer not to respond | 99 |  |
| D22 | *[Post-intervention follow-up only]*  What do you plan to do when you leave high school? | Going to college/university | 1 | plan_postschool |
|  |  | Go to technical/technological education (e.g. Sena, technical institute) | 2 |  |
|  |  | Work in this municipality/city | 3 |  |
|  |  | Work in a different municipality/city | 4 |  |
|  |  | Staying home to do household chores (caring for a child or older adult, housework) | 5 |  |
|  |  | Staying at home without any activity | 6 |  |
|  |  | Other [Answer D23] | 77 |  |
|  |  | Don’t know | 88 |  |
|  |  | Prefer not to respond | 99 |  |
| D23 | What else do you plan to do? | 🖎 | | plan_postschoolo |
| D24 | *[12-month-post baseline only]*  Imagine that you can study as much as you want. What is the highest level of education you would like to achieve? | None | 0 | schooling_hope |
|  |  | Elementary school (grade 5) [Col] | 1 |  |
|  |  | Secondary school (grade 9) [Col] | 2 |  |
|  |  | High school (grade 11) [Col] | 3 |  |
|  |  | Technician or technologist (e.g. studying at SENA) [Col] | 4 |  |
|  |  | University degree [Col] | 5 |  |
|  |  | Specialization or master's degree [Col] | 6 |  |
|  |  | 10+2 [Ne] | 7 |  |
|  |  | Bachelors [Ne] | 8 |  |
|  |  | Masters or above [Ne] | 9 |  |
|  |  | Primary (till grade 7 or equivalent) [SA] | 10 |  |
|  |  | Secondary (e.g. high school/technical or vocational school) [SA] | 11 |  |
|  |  | University/tertiary (e.g. University/Technikon, College) [SA] | 12 |  |
|  |  | Other [Answer D25] | 77 |  |
|  |  | Don't know | 88 |  |
|  |  | Prefer not to respond | 99 |  |
| D25 | *[Not Colombia]* What other level of education would you like to achieve? | 🖎 |  | schooling_hopeo |
| D26 | *[12-month-post baseline only]*  On a scale of 0 to 10, where 0 is "not at all sure" and 10 is "totally sure", how confident are you that you will achieve this level of education? | 🖎 |  | schooling_certainty |
| D27 | *[12-month-post baseline only]*  What is the main obstacle or difficulty you might encounter in reaching your desired level of education? | None | 0 | schooling_why |
|  |  | Lack of financial resources | 1 |  |
|  |  | Your parents don't support you or disagree with this plan | 2 |  |
|  |  | Your partner doesn't support you or disagrees with this plan | 3 |  |
|  |  | You don't consider yourself a good enough student to make it | 4 |  |
|  |  | You lack determination or decisiveness | 5 |  |
|  |  | No offer nearby | 6 |  |
|  |  | Suffer from an illness | 7 |  |
|  |  | Have or will have children | 8 |  |
|  |  | Caring for a caregiver (other than a child) | 9 |  |
|  |  | You have to work | 10 |  |
|  |  | Other | 77 |  |
|  |  | Don’t know | 88 |  |
|  |  | Prefer not to respond | 99 |  |
| D28 | What other obstacle might you encounter? | 🖎 |  | schooling_whyo |
| D29 | *[Baseline and 12-month-post baseline]*  How much schooling do you expect to complete? | None | 0 | schooling_expect |
|  |  | Elementary school (grade 5) [Col] | 1 |  |
|  |  | Secondary school (grade 9) [Col] | 2 |  |
|  |  | High school (grade 11) [Col] | 3 |  |
|  |  | Technician or technologist (e.g. studying at SENA) [Col] | 4 |  |
|  |  | University degree [Col] | 5 |  |
|  |  | Specialization or master's degree [Col] | 6 |  |
|  |  | 10+2 [Ne] | 7 |  |
|  |  | Bachelors [Ne] | 8 |  |
|  |  | Masters or above [Ne] | 9 |  |
|  |  | Primary (till grade 7 or equivalent) [SA] | 10 |  |
|  |  | Secondary (e.g. high school/technical or vocational school) [SA] | 11 |  |
|  |  | University/tertiary (e.g. University/Technikon, College) [SA] | 12 |  |
|  |  | Other [Answer D30] | 77 |  |
|  |  | Don't know | 88 |  |
|  |  | Prefer not to respond | 99 |  |
| D30 | *[Not Colombia]* Specify what other schooling you expect to complete | 🖎 |  | schooling_expecto |
| D31 | *[18-month-post baseline only]*  In the last year, have you spoken to a career advisor outside of school to find out about future studies or types of work? | No | 0 | attention_advisor |
|  |  | Yes, once | 1 |  |
|  |  | Yes, two or more times | 2 |  |
|  |  | Prefer not to respond | 99 |  |
| D32 | *[18-month-post baseline only]*  In the last year, did you search the internet for information about secondary/tertiary education? | No | 0 | attention_internet |
|  |  | Yes, once | 1 |  |
|  |  | Yes, two or more times | 2 |  |
|  |  | Prefer not to respond | 99 |  |
| D33 | *[18-month-post baseline only]*  In the last year, did you search information on student financing (e.g. student loans or grants)? | No | 0 | attention_financing |
|  |  | Yes, once | 1 |  |
|  |  | Yes, two or more times | 2 |  |
|  |  | Prefer not to respond | 99 |  |
| *[18-month-post baseline only]* The following questions pertain to school readiness. To what extent do you agree or disagree with the following statements? | | | | |
| D34 | I worry that I am not prepared for life after <final year of compulsory education> | Strongly disagree | 1 | readiness_prepared |
|  |  | Disagree | 2 |  |
|  |  | Agree | 3 |  |
|  |  | Strongly agree | 4 |  |
|  |  | Prefer not to respond | 99 |  |
| D35 | I feel well-informed about possible paths for me after <final year of compulsory education> | Strongly disagree | 1 | readiness_paths |
|  |  | Disagree | 2 |  |
|  |  | Agree | 3 |  |
|  |  | Strongly agree | 4 |  |
|  |  | Prefer not to respond | 99 |  |
| D36 | I feel pressure from my family to follow a specific path (e.g. go to college, work in the family business, learn a trade) after <final year of compulsory education> | Strongly disagree | 1 | readiness_pressure |
|  |  | Disagree | 2 |  |
|  |  | Agree | 3 |  |
|  |  | Strongly agree | 4 |  |
|  |  | Prefer not to respond | 99 |  |
| D37 | I worry that I won't have enough money to do what I'd like to do after <final year of compulsory education> | Strongly disagree | 1 | readiness_money |
|  |  | Disagree | 2 |  |
|  |  | Agree | 3 |  |
|  |  | Strongly agree | 4 |  |
|  |  | Prefer not to respond | 99 |  |
| D38 | School has done little to prepare me for adult life when I leave school | Strongly disagree | 1 | readiness_adultlife |
|  |  | Disagree | 2 |  |
|  |  | Agree | 3 |  |
|  |  | Strongly agree | 4 |  |
|  |  | Prefer not to respond | 99 |  |
| D39 | School has been a waste of time | Strongly disagree | 1 | readiness_waste |
|  |  | Disagree | 2 |  |
|  |  | Agree | 3 |  |
|  |  | Strongly agree | 4 |  |
|  |  | Prefer not to respond | 99 |  |
| D40 | School has helped give me confidence to make decisions | Strongly disagree | 1 | readiness_confidence |
|  |  | Disagree | 2 |  |
|  |  | Agree | 3 |  |
|  |  | Strongly agree | 4 |  |
|  |  | Prefer not to respond | 99 |  |
| D41 | School has taught me things which could be useful in a job | Strongly disagree | 1 | readiness_job |
|  |  | Disagree | 2 |  |
|  |  | Agree | 3 |  |
|  |  | Strongly agree | 4 |  |
|  |  | Prefer not to respond | 99 |  |
| D42 | I feel well-prepared for my future path after < final year of compulsory education> | Strongly disagree | 1 | readiness_future |
|  |  | Disagree | 2 |  |
|  |  | Agree | 3 |  |
|  |  | Strongly agree | 4 |  |
|  |  | Prefer not to respond | 99 |  |

| D.2 Life satisfaction | | | | |
| --- | --- | --- | --- | --- |
| Instructions:  *"The following question asks how satisfied you feel about your life, on a scale from 0 to 10. Zero means you feel 'not at all satisfied' and 10 means 'completely satisfied.”* | | | | |
| D.2.1 | [All follow-ups]  Overall, how satisfied are you with your life as a whole? | 🖎 |  | life_satisfaction |

| **E. Measurement of Mental Health among Adolescents at the Population level (MMAPP)**  (Carvajal-Velez et al., 2023) | | | | | | |
| --- | --- | --- | --- | --- | --- | --- |
| Instructions:  “*Every question is important, please try your best to respond each of them. If you do not want to answer a question, please let us know. Remember that all your answers are confidential.*  *During the past two weeks, how often..*” | | | | | | |
| E1 | Have you been feeling very sad or depressed? | Never | 0 | mmapp_1 | |  |
|  |  | Sometimes | 1 |  |  |  |
|  |  | Often | 2 |  |  |  |
|  |  | Always | 3 |  |  |  |
|  |  | Prefer not to respond | 99 |  |  |  |
| E2 | Have you felt easily annoyed or irritable/irritated at small things? | Never | 0 | mmapp_2 | |  |
|  |  | Sometimes | 1 |  |  |  |
|  |  | Often | 2 |  |  |  |
|  |  | Always | 3 |  |  |  |
|  |  | Prefer not to respond | 99 |  |  |  |
| E3 | Have you not enjoyed doing things you used to enjoy (like playing sports, singing, and dancing, spending time with friends, watching videos)? | Never | 0 | mmapp_3 | |  |
|  |  | Sometimes | 1 |  |  |  |
|  |  | Often | 2 |  |  |  |
|  |  | Always | 3 |  |  |  |
|  |  | Prefer not to respond | 99 |  |  |  |
| E4 | Have you felt hopeless about the future? | Never | 0 | mmapp_4 | |  |
|  |  | Sometimes | 1 |  |  |  |
|  |  | Often | 2 |  |  |  |
|  |  | Always | 3 |  |  |  |
|  |  | Prefer not to respond | 99 |  |  |  |
| E5 | Have you felt nervous or anxious? | Never | 0 | mmapp_5 | |  |
|  |  | Sometimes | 1 |  |  |  |
|  |  | Often | 2 |  |  |  |
|  |  | Always | 3 |  |  |  |
|  |  | Prefer not to respond | 99 |  |  |  |
| E6 | Have you worried you can’t do anything right or are doing things poorly? | Never | 0 | mmapp_6 | |  |
|  |  | Sometimes | 1 |  |  |  |
|  |  | Often | 2 |  |  |  |
|  |  | Always | 3 |  |  |  |
|  |  | Prefer not to respond | 99 |  |  |  |
| E7 | Have you worried about what others think about you? | Never | 0 | mmapp_7 | |  |
|  |  | Sometimes | 1 |  |  |  |
|  |  | Often | 2 |  |  |  |
|  |  | Always | 3 |  |  |  |
|  |  | Prefer not to respond | 99 |  |  |  |
| E8 | Have you worried something bad will happen to you or your family? | Never | 0 | mmapp_8 | |  |
|  |  | Sometimes | 1 |  |  |  |
|  |  | Often | 2 |  |  |  |
|  |  | Always | 3 |  |  |  |
|  |  | Prefer not to respond | 99 |  |  |  |
| E9 | Have you worried too much about many different things at the same time (worried about school work, exams, household issues, your community and your future)? | Never | 0 | mmapp_9 | |  |
|  |  | Sometimes | 1 |  |  |  |
|  |  | Often | 2 |  |  |  |
|  |  | Always | 3 |  |  |  |
|  |  | Prefer not to respond | 99 |  |  |  |
| E10 | Have you felt unable to stop or control your worries? | Never | 0 | mmapp_10 | |  |
|  |  | Sometimes | 1 |  |  |  |
|  |  | Often | 2 |  |  |  |
|  |  | Always | 3 |  |  |  |
|  |  | Prefer not to respond | 99 |  |  |  |
| E11 | Have you not wanted to eat even when food is available or eaten too much? | Never | 0 | mmapp_11 | |  |
|  |  | Sometimes | 1 |  |  |  |
|  |  | Often | 2 |  |  |  |
|  |  | Always | 3 |  |  |  |
|  |  | Prefer not to respond | 99 |  |  |  |
| E12 | Have you had difficulty falling asleep? | Never | 0 | mmapp_12 | |  |
|  |  | Sometimes | 1 |  |  |  |
|  |  | Often | 2 |  |  |  |
|  |  | Always | 3 |  |  |  |
|  |  | Prefer not to respond | 99 |  |  |  |
| E13 | Have you been waking up during the night? | Never | 0 | mmapp_13 | |  |
|  |  | Sometimes | 1 |  |  |  |
|  |  | Often | 2 |  |  |  |
|  |  | Always | 3 |  |  |  |
|  |  | Prefer not to respond | 99 |  |  |  |
| E14 | Have you been sleeping too much? | Never | 0 | mmapp_14 | |  |
|  |  | Sometimes | 1 |  |  |  |
|  |  | Often | 2 |  |  |  |
|  |  | Always | 3 |  |  |  |
|  |  | Prefer not to respond | 99 |  |  |  |
| E15 | Have you felt you got tired easily or did not have energy to do daily activities? | Never | 0 | mmapp_15 | |  |
|  |  | Sometimes | 1 |  |  |  |
|  |  | Often | 2 |  |  |  |
|  |  | Always | 3 |  |  |  |
|  |  | Prefer not to respond | 99 |  |  |  |
| E16 | Have you had trouble concentrating on things, such as doing homework, household chores, or other activities (such as watching videos or using social media: WhatsApp/Instagram)? | Never | 0 | mmapp_16 | |  |
|  |  | Sometimes | 1 |  |  |  |
|  |  | Often | 2 |  |  |  |
|  |  | Always | 3 |  |  |  |
|  |  | Prefer not to respond | 99 |  |  |  |
| E17 | Have you felt lonely? | Never | 0 | mmapp_17 | |  |
|  |  | Sometimes | 1 |  |  |  |
|  |  | Often | 2 |  |  |  |
|  |  | Always | 3 |  |  |  |
|  |  | Prefer not to respond | 99 |  |  |  |
| E18 | Have you felt like a failure/disappointment or like you have let yourself or your family down? | Never | 0 | mmapp_18 | |  |
|  |  | Sometimes | 1 |  |  |  |
|  |  | Often | 2 |  |  |  |
|  |  | Always | 3 |  |  |  |
|  |  | Prefer not to respond | 99 |  |  |  |
| E19 | Have you had thoughts that you would rather be dead or thoughts of hurting yourself? | Never [Skip E2 section] | 0 | mmapp_19 | |  |
|  |  | Sometimes | 1 |  |  |  |
|  |  | Often | 2 |  |  |  |
|  |  | Always | 3 |  |  |  |
|  |  | Prefer not to respond | 99 |  |  |  |
| E20 | Have other people said that you have been moving your body more slowly than usual (for example, walking slower than before)? | Never | 0 | mmapp_20 | |  |
|  |  | Sometimes | 1 |  |  |  |
|  |  | Often | 2 |  |  |  |
|  |  | Always | 3 |  |  |  |
|  |  | Prefer not to respond | 99 |  |  |  |
| E21 | Have others said that you are restless or that you can’t sit still (e.g., fidgeting with objects like pens, pacing up and down)? | Never | 0 | mmapp_21 | |  |
|  |  | Sometimes | 1 |  |  |  |
|  |  | Often | 2 |  |  |  |
|  |  | Always | 3 |  |  |  |
|  |  | Prefer not to respond | 99 |  |  |  |
| E22 | Have you felt that it was difficult to breathe? | Never | 0 | mmapp_22 | |  |
|  |  | Sometimes | 1 |  |  |  |
|  |  | Often | 2 |  |  |  |
|  |  | Always | 3 |  |  |  |
|  |  | Prefer not to respond | 99 |  |  |  |
| E23 | Have you felt dizziness or faint? | Never | 0 | mmapp_23 | |  |
|  |  | Sometimes | 1 |  |  |  |
|  |  | Often | 2 |  |  |  |
|  |  | Always | 3 |  |  |  |
|  |  | Prefer not to respond | 99 |  |  |  |
| E24 | Have you gotten suddenly scared for no reason or without knowing what made you scared? | Never | 0 | mmapp_24 | |  |
|  |  | Sometimes | 1 |  |  |  |
|  |  | Often | 2 |  |  |  |
|  |  | Always | 3 |  |  |  |
|  |  | Prefer not to respond | 99 |  |  |  |
| E25 | Have you had difficulty relaxing or difficulty feeling calm? | Never | 0 | mmapp_25 | |  |
|  |  | Sometimes | 1 |  |  |  |
|  |  | Often | 2 |  |  |  |
|  |  | Always | 3 |  |  |  |
|  |  | Prefer not to respond | 99 |  |  |  |
| E26 | Have you felt like your heart was pounding or beating too fast? (Even when not exercising) | Never | 0 | mmapp_26 | |  |
|  |  | Sometimes | 1 |  |  |  |
|  |  | Often | 2 |  |  |  |
|  |  | Always | 3 |  |  |  |
|  |  | Prefer not to respond | 99 |  |  |  |
| E27 | Have you had headaches or muscle tension/pain? | Never | 0 | mmapp_27 | |  |
|  |  | Sometimes | 1 |  |  |  |
|  |  | Often | 2 |  |  |  |
|  |  | Always | 3 |  |  |  |
|  |  | Prefer not to respond | 99 |  |  |  |
| E28 | Have you had bad dreams or nightmares? | Never | 0 | mmapp_28 | |  |
|  |  | Sometimes | 1 |  |  |  |
|  |  | Often | 2 |  |  |  |
|  |  | Always | 3 |  |  |  |
|  |  | Prefer not to respond | 99 |  |  |  |
| E29 | Total depression score on MMAPP | 🖎 | | | mmapp_totdep | |
| E30 | Total anxiety score on MMAPP | 🖎 | | | mmapp_totanx | |
| B31 | Total depression score on PHQ-A | 🖎 | | | mmapp_totalphqa | |
| B32 | Total anxiety score on GAD-7 | 🖎 | | | mmapp_totalgad | |
| B33 | Score above cut-off for depression on PHQ-A | No | 0 | depressed | |  |
|  |  | Yes | 1 |  |  |  |
| B34 | Score above cut-off for anxiety on GAD-7 | No | 0 | anxious | |  |
|  |  | Yes | 1 |  |  |  |

| **E2. Suicide risk [Colombia only]** | | | | |
| --- | --- | --- | --- | --- |
| E2.1 | Have you ever thought of killing yourself in the past few weeks? | No | 0 | mmapp_19a |
|  |  | Yes [Answer E2.3] | 1 |  |
|  |  | Prefer not to respond | 99 |  |
| E2.3 | Have you ever attempted to kill yourself before? | No | 0 | mmapp_19c |
|  |  | Yes | 1 |  |
|  |  | Prefer not to respond | 99 |  |
| E2.3 | Have you thought about how you would do this? | No | 0 | mmapp_19d |
|  |  | Yes | 1 |  |
|  |  | Prefer not to respond | 99 |  |

| **E2. Suicide risk [Nepal only]** | | | | |
| --- | --- | --- | --- | --- |
| E2.1 | Have you felt that life is not worth living? | No | 0 | mmapp_19e |
|  |  | Yes | 1 |  |
|  |  | Prefer not to respond | 99 |  |
| E2.2 | Have you ever thought of harming yourself? | No [Skip to section end] | 0 | mmapp_19a |
|  |  | Yes [Answer E2.3] | 1 |  |
|  |  | Prefer not to respond | 99 |  |
| E2.3 | Have you ever hurt/harmed yourself before? | No [Go to E2.6] | 0 | mmapp_19c |
|  |  | Yes [Answer E2.4] | 1 |  |
|  |  | Prefer not to respond | 99 |  |
| E2.4 | Have you attempted this in the past three months? | No | 0 | mmapp_19c_3m |
|  |  | Yes [Answer E2.5] | 1 |  |
|  |  | Prefer not to respond | 99 |  |
| E2.5 | Have you attempted this in the past month? | No | 0 | mmapp_19c_1m |
|  |  | Yes [Skip to section end] | 1 |  |
|  |  | Prefer not to respond | 99 |  |
| E2.6 | Do you feel that you want to harm yourself right now? | No | 0 | mmapp_19b |
|  |  | Yes | 1 |  |
|  |  | Prefer not to respond | 99 |  |

| **E2. Suicide risk [South Africa only]** | | | | |
| --- | --- | --- | --- | --- |
| E2.1 | Have you ever thought of killing yourself in the past few weeks? | No | 0 | mmapp_19a |
|  |  | Yes [Answer E2.2] | 1 |  |
|  |  | Prefer not to respond | 99 |  |
| E2.2 | Do you feel that you want to kill yourself right now? | No | 0 | mmapp_19b |
|  |  | Yes | 1 |  |
|  |  | Prefer not to respond | 99 |  |
| E2.3 | Have you ever attempted to kill yourself before? | No | 0 | mmapp_19c |
|  |  | Yes | 1 |  |
|  |  | Prefer not to respond | 99 |  |
| E2.4 | [If respond yes to B2.1 or B2.2]  You have shared that you are thinking of killing yourself. Have you thought about how you would do this? | No | 0 | mmapp_19d |
|  |  | Yes | 1 |  |
|  |  | Prefer not to respond | 99 |  |

| F. Disruptive Behavior International Scale - Nepal version (DBIS-N)  (Burkey et al., 2018) | | | | |
| --- | --- | --- | --- | --- |
| Instructions:  “*Every question is important, please try your best to respond each of them. If you do not want to answer a question, please let us know. Remember that all your answers are confidential.*  *During the past two weeks, how often have you..*” | | | | |
| F1 | Been naughty, stubborn, or acted disrespectful? For example (Col: disobedient; Nepali: stubborn or naughty/misbehaving) | Never | 1 | dbis_1 |
|  |  | Sometimes | 2 |  |
|  |  | Often | 3 |  |
|  |  | Always | 4 |  |
|  |  | Prefer not to respond | 99 |  |
| F2 | Felt angry? | Never | 1 | dbis_2 |
|  |  | Sometimes | 2 |  |
|  |  | Often | 3 |  |
|  |  | Always | 4 |  |
|  |  | Prefer not to respond | 99 |  |
| F3 | Used bad words/ swear words/curse words? | Never | 1 | dbis_3 |
|  |  | Sometimes | 2 |  |
|  |  | Often | 3 |  |
|  |  | Always | 4 |  |
|  |  | Prefer not to respond | 99 |  |
| F4 | Lied to others? | Never | 1 | dbis_4 |
|  |  | Sometimes | 2 |  |
|  |  | Often | 3 |  |
|  |  | Always | 4 |  |
|  |  | Prefer not to respond | 99 |  |
| F5 | Had physical fights with other teenagers/youth? | Never | 1 | dbis_5 |
|  |  | Sometimes | 2 |  |
|  |  | Often | 3 |  |
|  |  | Always | 4 |  |
|  |  | Prefer not to respond | 99 |  |
| F6 | Spent time with teenagers who are misbehaving or have bad influences (for example, negative peer pressure, teenagers who smoke or drink, young people in gangs, youth who use bad language)? | Never | 1 | dbis_6 |
|  |  | Sometimes | 2 |  |
|  |  | Often | 3 |  |
|  |  | Always | 4 |  |
|  |  | Prefer not to respond | 99 |  |
| F7 | Bothered or annoyed others deliberately/on purpose (Colombia: making someone desperate)? | Never | 1 | dbis_7 |
|  |  | Sometimes | 2 |  |
|  |  | Often | 3 |  |
|  |  | Always | 4 |  |
|  |  | Prefer not to respond | 99 |  |
| F8 | Talked back or argued with adults? | Never | 1 | dbis_8 |
|  |  | Sometimes | 2 |  |
|  |  | Often | 3 |  |
|  |  | Always | 4 |  |
|  |  | Prefer not to respond | 99 |  |

| G. The Identifying Depression Early in Adolescence Risk Score (IDEA-RS)  (Rocha et al., 2021) – Baseline only | | | | |
| --- | --- | --- | --- | --- |
| Instructions:  “*Every question is important, please try your best to respond each of them. If you do not want to answer a question, please let us know. Remember that all your answers are confidential.*  *I am now going to ask a few questions about the support you receive from friends and from your biological parents?”* [explain what is meant by biological parents] | | | | |
| G1 | Throughout your life, have you had close friendships with people who support you when you are worried, sad or distressed? | No | 0 | idea_1 |
|  |  | Yes | 1 |  |
|  |  | Prefer not to respond | 99 |  |
| G2 | Throughout your life, have you mostly had a close and warm relationship with your biological mother (birth mother) (e.g., sharing about your feelings and experiences without judgement) | No (don’t know biological mother, don’t stay with biological mother, or don’t feel close to biological mother) | 0 | idea_2 |
|  |  | Yes | 1 |  |
|  |  | Prefer not to respond | 99 |  |
| G3 | Throughout your life, have you mostly had a close and warm relationship with your biological father (e.g., sharing about your feelings and experiences without judgement) | No (don’t know biological father, don’t stay with biological father, or don’t feel close to biological father) | 0 | idea_3 |
|  |  | Yes | 1 |  |
|  |  | Prefer not to respond | 99 |  |
| G4 | Throughout your life, have you mostly had a close and warm relationship with someone whom you consider is a parent or **caregiver** who is not your biological father or mother?  (sharing about your feelings and experiences without judgement) | No | 0 | idea_3_guardian |
|  |  | Yes | 1 |  |
|  |  | Prefer not to respond | 99 |  |
| G5 | Throughout your life, have your biological mother and biological father mostly had a close and warm relationship with each other? (sharing their feelings and experiences with each other) | No | 0 | idea_4 |
|  |  | Yes | 1 |  |
|  |  | Prefer not to respond | 99 |  |
| G6 | *[South Africa only]*  Do you have more than one caregiver who is not your biological father or mother? | No | 0 | idea_4_morethanone |
|  |  | Yes [Answer G7] | 1 |  |
| G7 | Throughout your life, have your guardian/caregivers mostly had a close and warm relationship with each other? (e.g. safe relationship, sharing their feelings and experiences with each other) | No | 0 | idea_4_guardian |
|  |  | Yes | 1 |  |
|  |  | Prefer not to respond | 99 |  |
| G8 | Throughout your life, have your guardian/caregivers mostly had a close and warm relationship with each other? (e.g. safe relationship, sharing their feelings and experiences with each other) | No | 0 | idea_4b |
|  |  | Yes | 1 |  |
|  |  | Prefer not to respond | 99 |  |
| Instructions:  “*I am going to ask some questions about smoking, drinking alcohol, or other drug use. The answers to these questions are confidential and will not be shared with anyone.”* | | | | |
| G9 | Have you ever smoked tobacco cigarettes, vaped or chewed tobacco? | No | 0 | idea_5 |
|  |  | Yes | 1 |  |
|  |  | Prefer not to respond | 99 |  |
| G10 | Have you ever drank beer, wine, or other alcoholic beverages? | No | 0 | idea_6 |
|  |  | Yes | 1 |  |
|  |  | Prefer not to respond | 99 |  |
| G11 | Have you ever used marijuana? | No | 0 | idea_7 |
|  |  | Yes | 1 |  |
|  |  | Prefer not to respond | 99 |  |
| G12 | Have you ever used medicines/pharmaceuticals to get high or for fun (medicines that weren’t prescribed to you) | No | 0 | idea_8 |
|  |  | Yes | 1 |  |
|  |  | Prefer not to respond | 99 |  |
| G13 | Have you ever used any other hard drugs? | No | 0 | idea_9 |
|  |  | Yes | 1 |  |
|  |  | Prefer not to respond | 99 |  |
| Instructions:  *“I am now going to ask some questions about difficult or traumatic experiences you may have had. You do not need to answer any questions that make you feel uncomfortable. I may ask some follow-up questions to help you get some support if you would like for difficult or traumatic experiences you may have experienced”* | | | | |
| G14 | Have you ever gotten into serious physical fights with someone else (kicking, punching and someone got hurt or injured)? | No | 0 | idea_10 |
|  |  | Yes | 1 |  |
|  |  | Prefer not to respond | 99 |  |
| G15 | Have you ever been emotionally abused by a peer, adult, partner (boyfriend/girlfriend) or other person (for example repeated humiliation, discrimination, shouting at you, teasing you etc.? | No | 0 | idea_11 |
|  |  | Yes | 1 |  |
|  |  | Prefer not to respond | 99 |  |
| G16 | Have you ever been physically abused by a peer, adult, partner (boyfriend/girlfriend) or other person (e.g. beaten you, strangled you, forced you to do physical work more than your capacity? [add country examples] | No | 0 | idea_12 |
|  |  | Yes | 1 |  |
|  |  | Prefer not to respond | 99 |  |
| G17 | Have you ever been sexually abused by a peer, adult, partner (boyfriend/girlfriend or other person (for example, sexual harassment, touching your private parts, forcing you to watch pornography etc.? [Add country examples] | No | 0 | idea_13 |
|  |  | Yes | 1 |  |
|  |  | Prefer not to respond | 99 |  |

| G.2 The Identifying Depression Early in Adolescence Risk Score (IDEA-RS)  (Rocha et al., 2021) – All follow-up assessments | | | | |
| --- | --- | --- | --- | --- |
| Instructions:  “*Every question is important, please try your best to respond each of them. If you do not want to answer a question, please let us know. Remember that all your answers are confidential.*  *I am now going to ask a few questions about the support you receive from friends and from your biological parents?”* [explain what is meant by biological parents] | | | | |
| G1 | In the past 6 months, have you had close friendships with people who support you when you are worried, sad or distressed? | No | 0 | idea_6m_1 |
|  |  | Yes | 1 |  |
|  |  | Prefer not to respond | 99 |  |
| G2 | In the past 6 months, have you mostly had a close and warm relationship with your biological mother (birth mother) (e.g., sharing about your feelings and experiences without judgement) | No (don’t know biological mother, don’t stay with biological mother, or don’t feel close to biological mother) | 0 | idea_6m_2 |
|  |  | Yes | 1 |  |
|  |  | Prefer not to respond | 99 |  |
| G3 | In the past 6 months, have you mostly had a close and warm relationship with your biological father (e.g., sharing about your feelings and experiences without judgement) | No (don’t know biological father, don’t stay with biological father, or don’t feel close to biological father) | 0 | idea_6m_3 |
|  |  | Yes | 1 |  |
|  |  | Prefer not to respond | 99 |  |
| G4 | In the past 6 months, have you mostly had a close and warm relationship with someone whom you consider is a parent or **caregiver** who is not your biological father or mother?  (sharing about your feelings and experiences without judgement) | No | 0 | idea_6m_3_guardian |
|  |  | Yes | 1 |  |
|  |  | Prefer not to respond | 99 |  |
| G5 | In the past 6 months, have your biological mother and biological father mostly had a close and warm relationship with each other? (sharing their feelings and experiences with each other) | No | 0 | idea_6m_4 |
|  |  | Yes | 1 |  |
|  |  | Prefer not to respond | 99 |  |
| G6 | *[South Africa only]*  Do you have more than one caregiver who is not your biological father or mother? | No | 0 | idea_6m_4_morethanone |
|  |  | Yes [Answer G7] | 1 |  |
| G7 | In the past 6 months, have your guardian/caregivers mostly had a close and warm relationship with each other? (e.g. safe relationship, sharing their feelings and experiences with each other) | No | 0 | idea_6m_4_guardian |
|  |  | Yes | 1 |  |
|  |  | Prefer not to respond | 99 |  |
| G8 | In the past 6 months, have your guardian/caregivers mostly had a close and warm relationship with each other? (e.g. safe relationship, sharing their feelings and experiences with each other) | No | 0 | idea_6m_4b |
|  |  | Yes | 1 |  |
|  |  | Prefer not to respond | 99 |  |
| Instructions:  “*I am going to ask some questions about smoking, drinking alcohol, or other drug use. The answers to these questions are confidential and will not be shared with anyone.”* | | | | |
| G9 | In the past 6 months, have you smoked tobacco cigarettes, vaped or chewed tobacco? | No | 0 | idea_6m_5 |
|  |  | Yes | 1 |  |
|  |  | Prefer not to respond | 99 |  |
| G10 | In the past 6 months, have you drank beer, wine, or other alcoholic beverages? | No | 0 | idea_6m_6 |
|  |  | Yes | 1 |  |
|  |  | Prefer not to respond | 99 |  |
| G11 | In the past 6 months, have you used marijuana? | No | 0 | idea_6m_7 |
|  |  | Yes | 1 |  |
|  |  | Prefer not to respond | 99 |  |
| G12 | In the past 6 months, have you used medicines/pharmaceuticals to get high or for fun (medicines that weren’t prescribed to you) | No | 0 | idea_6m_8 |
|  |  | Yes | 1 |  |
|  |  | Prefer not to respond | 99 |  |
| G13 | In the past 6 months, have you used any other hard drugs? | No | 0 | idea_6m_9 |
|  |  | Yes | 1 |  |
|  |  | Prefer not to respond | 99 |  |
| Instructions:  *“I am now going to ask some questions about difficult or traumatic experiences you may have had. You do not need to answer any questions that make you feel uncomfortable. I may ask some follow-up questions to help you get some support if you would like for difficult or traumatic experiences you may have experienced”* | | | | |
| G14 | In the past 6 months, have you gotten into serious physical fights with someone else (kicking, punching and someone got hurt or injured)? | No | 0 | idea_6m_10 |
|  |  | Yes | 1 |  |
|  |  | Prefer not to respond | 99 |  |
| G15 | In the past 6 months, have you been emotionally abused by a peer, adult, partner (boyfriend/girlfriend) or other person (for example repeated humiliation, discrimination, shouting at you, teasing you etc.? | No | 0 | idea_6m_11 |
|  |  | Yes | 1 |  |
|  |  | Prefer not to respond | 99 |  |
| G16 | In the past 6 months, have you been physically abused by a peer, adult, partner (boyfriend/girlfriend) or other person (e.g. beaten you, strangled you, forced you to do physical work more than your capacity? [add country examples] | No | 0 | idea_6m_12 |
|  |  | Yes | 1 |  |
|  |  | Prefer not to respond | 99 |  |
| G17 | In the past 6 months, have you been sexually abused by a peer, adult, partner (boyfriend/girlfriend or other person (for example, sexual harassment, touching your private parts, forcing you to watch pornography etc.? [Add country examples] | No | 0 | idea_6m_13 |
|  |  | Yes | 1 |  |
|  |  | Prefer not to respond | 99 |  |

| H. Difficulties in Emotion Regulation Scale (DERS-SF)  (Kaufman et al., 2016) | | | | |
| --- | --- | --- | --- | --- |
| *Instructions: “I am going to ask questions about how you think about your feelings and emotions. It can be very difficult to talk about our emotions. Sometimes it is hard to understand what we are feeling, and we can feel lots of emotions at once and not how to respond. I know these can be difficult questions, so please respond the best you can. For the past few months,…* | | | | |
| H1 | How often do you pay attention to how you feel? | Never | 0 | ders_1 |
|  |  | Rarely/almost never | 1 |  |
|  |  | Sometimes | 2 |  |
|  |  | Often | 3 |  |
|  |  | Always | 4 |  |
|  |  | Prefer not to respond | 99 |  |
| H2 | How often do you have no idea how you are feeling/how you are feeling emotionally? | Never | 0 | ders_2 |
|  |  | Rarely/almost never | 1 |  |
|  |  | Sometimes | 2 |  |
|  |  | Often | 3 |  |
|  |  | Always | 4 |  |
|  |  | Prefer not to respond | 99 |  |
| H3 | How often do you have difficulty making sense out of your feelings? | Never | 0 | ders_3 |
|  |  | Rarely/almost never | 1 |  |
|  |  | Sometimes | 2 |  |
|  |  | Often | 3 |  |
|  |  | Always | 4 |  |
|  |  | Prefer not to respond | 99 |  |
| H4 | How often do you care about what you are feeling? | Never | 0 | ders_4 |
|  |  | Rarely/almost never | 1 |  |
|  |  | Sometimes | 2 |  |
|  |  | Often | 3 |  |
|  |  | Always | 4 |  |
|  |  | Prefer not to respond | 99 |  |
| H5 | How often do you feel confused about how you feel? | Never | 0 | ders_5 |
|  |  | Rarely/almost never | 1 |  |
|  |  | Sometimes | 2 |  |
|  |  | Often | 3 |  |
|  |  | Always | 4 |  |
|  |  | Prefer not to respond | 99 |  |
| *Instructions: I am going to ask some questions about times when you have felt upset over the past few months. Youth/teenagers can have many different thoughts and behaviours when they are feeling upset. I am interested to learn how you think and behave when you are feeling upset.  The response options are …. [add explanation] . For the past few months, when you are upset…* | | | | |
| H6 | Do you acknowledge/are you aware your emotions? How often? | Never | 0 | ders_6 |
|  |  | Rarely/almost never | 1 |  |
|  |  | Sometimes | 2 |  |
|  |  | Often | 3 |  |
|  |  | Always | 4 |  |
|  |  | Prefer not to respond | 99 |  |
| H7 | Do you become embarrassed for feeling that way? How often?  Nepal only: feeling embarrassed about getting upset, not embarrassed about what made you feel upset | Never | 0 | ders_7 |
|  |  | Rarely/almost never | 1 |  |
|  |  | Sometimes | 2 |  |
|  |  | Often | 3 |  |
|  |  | Always | 4 |  |
|  |  | Prefer not to respond | 99 |  |
| H8 | Do you have difficulty getting work done? How often? | Never | 0 | ders_8 |
|  |  | Rarely/almost never | 1 |  |
|  |  | Sometimes | 2 |  |
|  |  | Often | 3 |  |
|  |  | Always | 4 |  |
|  |  | Prefer not to respond | 99 |  |
| H9 | Do you become out of control? How often? | Never | 0 | ders_9 |
|  |  | Rarely/almost never | 1 |  |
|  |  | Sometimes | 2 |  |
|  |  | Often | 3 |  |
|  |  | Always | 4 |  |
|  |  | Prefer not to respond | 99 |  |
| H10 | Do you believe that you will end up feeling very depressed (feeling very sad or feeling down)? How often? | Never | 0 | ders_10 |
|  |  | Rarely/almost never | 1 |  |
|  |  | Sometimes | 2 |  |
|  |  | Often | 3 |  |
|  |  | Always | 4 |  |
|  |  | Prefer not to respond | 99 |  |
| H11 | Do you have difficulty focusing/concentrating on other things? How often? | Never | 0 | ders_11 |
|  |  | Rarely/almost never | 1 |  |
|  |  | Sometimes | 2 |  |
|  |  | Often | 3 |  |
|  |  | Always | 4 |  |
|  |  | Prefer not to respond | 99 |  |
| H12 | Do you feel guilty for feeling that way? (Nepal: feeling embarrassed about getting upset, not embarrassed about what made you feel upset) How often? | Never | 0 | ders_12 |
|  |  | Rarely/almost never | 1 |  |
|  |  | Sometimes | 2 |  |
|  |  | Often | 3 |  |
|  |  | Always | 4 |  |
|  |  | Prefer not to respond | 99 |  |
| H13 | Do you believe there is nothing you can do to make yourself feel better? How often? | Never | 0 | ders_13 |
|  |  | Rarely/almost never | 1 |  |
|  |  | Sometimes | 2 |  |
|  |  | Often | 3 |  |
|  |  | Always | 4 |  |
|  |  | Prefer not to respond | 99 |  |
| H14 | Do you become irritated with yourself for feeling that way? How often? | Never | 0 | ders_14 |
|  |  | Rarely/almost never | 1 |  |
|  |  | Sometimes | 2 |  |
|  |  | Often | 3 |  |
|  |  | Always | 4 |  |
|  |  | Prefer not to respond | 99 |  |
| H15 | Does it take you a long time to feel better? How often? | Never | 0 | ders_15 |
|  |  | Rarely/almost never | 1 |  |
|  |  | Sometimes | 2 |  |
|  |  | Often | 3 |  |
|  |  | Always | 4 |  |
|  |  | Prefer not to respond | 99 |  |

| I. Children’s Hope Scale (CHS)  (Snyder et al., 1997) | | | | |
| --- | --- | --- | --- | --- |
| Instructions:  “*Every question is important, please try your best to respond each of them. If you do not want to answer a question, please let us know. Remember that all your answers are confidential.*  *For the past few months…*” | | | | |
| I1 | How often do you think you are doing really well? | Never | 0 | hope_1 |
|  |  | Rarely/almost never | 1 |  |
|  |  | Sometimes | 2 |  |
|  |  | Often | 3 |  |
|  |  | Always | 4 |  |
|  |  | Prefer not to respond | 99 |  |
| I2 | How often do you think of many ways to get the things that are most important to you? | Never | 0 | hope_2 |
|  |  | Rarely/almost never | 1 |  |
|  |  | Sometimes | 2 |  |
|  |  | Often | 3 |  |
|  |  | Always | 4 |  |
|  |  | Prefer not to respond | 99 |  |
| I3 | How often do you feel you are doing just as well in life as other kids your age? | Never | 0 | hope_3 |
|  |  | Rarely/almost never | 1 |  |
|  |  | Sometimes | 2 |  |
|  |  | Often | 3 |  |
|  |  | Always | 4 |  |
|  |  | Prefer not to respond | 99 |  |
| I4 | When you have a problem, how often can you come up with lots of ways to solve it? (for example, problems with friends, family or school work) | Never | 0 | hope_4 |
|  |  | Rarely/almost never | 1 |  |
|  |  | Sometimes | 2 |  |
|  |  | Often | 3 |  |
|  |  | Always | 4 |  |
|  |  | Prefer not to respond | 99 |  |
| I5 | How often do you believe the things you have done in the past will help you in the future? | Never | 0 | hope_5 |
|  |  | Rarely/almost never | 1 |  |
|  |  | Sometimes | 2 |  |
|  |  | Often | 3 |  |
|  |  | Always | 4 |  |
|  |  | Prefer not to respond | 99 |  |
| I6 | Even when others want to quit, how often do you know that you can find ways to solve the problem? (for example, problems with friends, family or schoolwork) | Never | 0 | hope_6 |
|  |  | Rarely/almost never | 1 |  |
|  |  | Sometimes | 2 |  |
|  |  | Often | 3 |  |
|  |  | Always | 4 |  |
|  |  | Prefer not to respond | 99 |  |

| J. Negotiation skills  *[12-month post baseline assessment only]* | | | | |
| --- | --- | --- | --- | --- |
| Instructions:  “*Every question is important, please try your best to respond each of them. If you do not want to answer a question, please let us know. Remember that all your answers are confidential.*  *To what extent do you agree or disagree with the following statements?*” | | | | |
| J1 | How often do you think you are doing really well? | Strongly disagree | 1 | neg_consider |
|  |  | Disagree | 2 |  |
|  |  | Neither agree or disagree | 3 |  |
|  |  | Agree | 4 |  |
|  |  | Strongly agree | 5 |  |
|  |  | Prefer not to respond | 99 |  |
| J2 | How often do you think of many ways to get the things that are most important to you? | Strongly disagree | 1 | neg_understand |
|  |  | Disagree | 2 |  |
|  |  | Neither agree or disagree | 3 |  |
|  |  | Agree | 4 |  |
|  |  | Strongly agree | 5 |  |
|  |  | Prefer not to respond | 99 |  |
| J3 | How often do you feel you are doing just as well in life as other kids your age? | Strongly disagree | 1 | neg_view |
|  |  | Disagree | 2 |  |
|  |  | Neither agree or disagree | 3 |  |
|  |  | Agree | 4 |  |
|  |  | Strongly agree | 5 |  |
|  |  | Prefer not to respond | 99 |  |
| J4 | When you have a problem, how often can you come up with lots of ways to solve it? (for example, problems with friends, family or school work) | Strongly disagree | 1 | neg_feel |
|  |  | Disagree | 2 |  |
|  |  | Neither agree or disagree | 3 |  |
|  |  | Agree | 4 |  |
|  |  | Strongly agree | 5 |  |
|  |  | Prefer not to respond | 99 |  |
| J5 | How often do you believe the things you have done in the past will help you in the future? | Strongly disagree | 1 | neg_position |
|  |  | Disagree | 2 |  |
|  |  | Neither agree or disagree | 3 |  |
|  |  | Agree | 4 |  |
|  |  | Strongly agree | 5 |  |
|  |  | Prefer not to respond | 99 |  |
| J6 | Even when others want to quit, how often do you know that you can find ways to solve the problem? (for example, problems with friends, family or schoolwork) | Strongly disagree | 1 | neg_behave |
|  |  | Disagree | 2 |  |
|  |  | Neither agree or disagree | 3 |  |
|  |  | Agree | 4 |  |
|  |  | Strongly agree | 5 |  |
|  |  | Prefer not to respond | 99 |  |
| J7 | Even when others want to quit, how often do you know that you can find ways to solve the problem? (for example, problems with friends, family or schoolwork) | Strongly disagree | 1 | neg_antidicipate |
|  |  | Disagree | 2 |  |
|  |  | Neither agree or disagree | 3 |  |
|  |  | Agree | 4 |  |
|  |  | Strongly agree | 5 |  |
|  |  | Prefer not to respond | 99 |  |
| J8 | Even when others want to quit, how often do you know that you can find ways to solve the problem? (for example, problems with friends, family or schoolwork) | Strongly disagree | 1 | neg_envision |
|  |  | Disagree | 2 |  |
|  |  | Neither agree or disagree | 3 |  |
|  |  | Agree | 4 |  |
|  |  | Strongly agree | 5 |  |
|  |  | Prefer not to respond | 99 |  |

| K. Social and Economic Measures (Adolescents) | | | | |
| --- | --- | --- | --- | --- |
| Instructions:  *“Every question is important, please try your best to respond each of them. If you do not want to answer a question, please let us know. Remember that all your answers are confidential.”* | | | | |
| K1 | In the past week of school, how many hours per day did you spend undertaking household chores (such as house cleaning, cooking, washing, laundry, ironing, shopping for groceries etc.)? | 🖎 |  | eco_chorestime |
|  |  | Don’t know | 88 |  |
|  |  | Prefer not to respond | 99 |  |
| K2 | *[Baseline and 18-month follow-up]*  When you finish your education, what type of job or occupation would you like to have? | 🖎 [Answer K3] |  | eco_jobwant |
|  |  | Don’t know | 88 |  |
|  |  | Prefer not to respond | 99 |  |
| K3 | *[18-month follow-up only]*  On a scale of 0 to 10 where 0 is "not at all sure/certain" and 10 is "totally sure/certain", how sure are you of that plan? | 🖎 |  | eco_jobwant_certainty |
| K4 | In the future, would you like to move or relocate somewhere temporarily (e.g. move for a period of time for study/education, work, or other reasons)? | Do not want to move or relocate temporarily | 0 | eco_migrate_temp |
|  |  | Move or relocate to elsewhere within the same district/province | 1 |  |
|  |  | Move or relocate to outside the district/province | 2 |  |
|  |  | Move or relocate to outside the country | 3 |  |
|  |  | Prefer not to respond | 99 |  |
| K5 | In the future, would you like to move or relocate somewhere permanently– go live somewhere else not move back here? | Do not want to move or relocate permanently | 0 | eco_migrate_perm |
|  |  | Move or relocate to elsewhere within the same district/province | 1 |  |
|  |  | Move or relocate to outside the district/province | 2 |  |
|  |  | Migrate to outside the country | 3 |  |
|  |  | Prefer not to respond | 99 |  |
| K6 | In the future, would you like to get married before you reach the age of 25? | Definitely yes | 1 | eco_marry25 |
|  |  | Probably yes | 2 |  |
|  |  | Probably not | 3 |  |
|  |  | Definitely not | 4 |  |
|  |  | Don’t know | 88 |  |
|  |  | Prefer not to respond | 99 |  |
| K7 | [Only ask if Answer ‘Definitely yes’ or ‘Probably yes’ to K16 in the case of Nepal] Looking into the future, would you like to have children before the age of 25? | Definitely yes | 1 | eco_children25 |
|  |  | Probably yes | 2 |  |
|  |  | Probably not | 3 |  |
|  |  | Definitely yes | 1 |  |
|  |  | Don’t know | 88 |  |
|  |  | Prefer not to respond | 99 |  |
| K8 | Which of the following do you think is true for adolescents who complete secondary schooling: | They are more likely to end up working in a paid job than those who do not complete it | 1 | eco_beliefs_1 |
|  |  | They are equally likely to end up working in a paid job than those who do not complete it | 2 |  |
|  |  | They are less likely to end up working in a paid job than those who do not complete it | 3 |  |
|  |  | Prefer not to respond | 99 |  |
| K9 | Which of the following do you think is true for adolescents who complete secondary schooling: | They are likely to earn more in their lifetime than those who do not complete it | 1 | eco_beliefs_2 |
|  |  | They are likely to earn similarly in their lifetime than those who do not complete it | 2 |  |
|  |  | They are likely to earn less in their lifetime than those who do not complete it | 3 |  |
|  |  | Prefer not to answer | 99 |  |
| K10 | Over the last month, did you buy any of the following items with your own money? [Select all that apply] | Entertainment or recreational activities (e.g., cinema, playing games, picnics | 1 | eco_consumption |
|  |  | Parties/celebrations | 2 |  |
|  |  | Food/groceries (including snacks) | 3 |  |
|  |  | Clothing | 4 |  |
|  |  | Shoes | 5 |  |
|  |  | Dining out | 6 |  |
|  |  | Books | 7 |  |
|  |  | School supplies | 8 |  |
|  |  | Beauty salon / hair salon | 9 |  |
|  |  | Jewelry / piercing | 10 |  |
|  |  | Digital devices (cell phone, tablet) | 11 |  |
|  |  | Other [Answer K11] | 77 |  |
|  |  | Don't know | 88 |  |
|  |  | Prefer not to respond | 99 |  |
| K11 | Please specify what other items you bought | 🖎 |  | eco_consumptiono |
| K12 | Over the past two months how often have you had to borrow money from someone else because you don’t have enough money? [Use glass scale] | Never [Go to K13] | 0 | eco_borrowed |
|  |  | Once or twice | 1 |  |
|  |  | A few times | 2 |  |
|  |  | Many times | 3 |  |
|  |  | Can’t remember | 88 |  |
|  |  | Prefer not to respond | 99 |  |
| K13 | If you didn’t borrow money over the past two months, how difficult has it been to stop yourself from borrowing money? (Nepal: create a situation to not borrow money) | Not difficult at all | 0 | eco_borrowedavoid |
|  |  | Somewhat difficult | 1 |  |
|  |  | Very difficult | 2 |  |
|  |  | Extremely difficult (you can’t do it all) | 3 |  |
|  |  | Don’t know | 88 |  |
|  |  | Prefer not to respond | 99 |  |
| K14 | Are you able to avoid the desire to spend money even when you go shopping? (e.g., is it easy to stop yourself from spending money when you go shopping, or when you go to the market and really want to spend money, can you resist buying something) | No | 0 | eco_desire |
|  |  | Yes | 1 |  |
|  |  | Don’t know | 88 |  |
|  |  | Prefer not to respond | 99 |  |
| K15 | Do you have control over whether or not you can spend or save money? (or, are you able to make the decision about whether or not you save money? Can you take decisions by yourself on whether to save or spend?) | No | 0 | eco_control |
|  |  | Yes | 1 |  |
|  |  | Don’t know | 88 |  |
|  |  | Prefer not answer | 99 |  |
| K16 | Have you saved money each month for the past two months? | No | 0 | eco_saved |
|  |  | Yes [Answer K25] | 1 |  |
|  |  | Don’t know | 88 |  |
|  |  | Prefer not to respond | 99 |  |
| K17 | If you saved money over the past two months, how difficult was it to save some money? | Not difficult at all | 0 | eco_savediff |
|  |  | Somewhat difficult | 1 |  |
|  |  | Very difficult | 2 |  |
|  |  | Extremely difficult (you can’t do it all) | 3 |  |
|  |  | Don’t know | 88 |  |
|  |  | Prefer not to respond | 99 |  |
| K18 | If you saved money every month, would that allow you to buy things you need? | No | 0 | eco_buythings |
|  |  | Yes | 1 |  |
|  |  | Don’t know | 88 |  |
|  |  | Prefer not to respond | 99 |  |
| K19 | If you saved every month, would you still need to borrow money? | No | 0 | eco_saveborrow |
|  |  | Yes | 1 |  |
|  |  | Don’t know | 88 |  |
|  |  | Prefer not to respond | 99 |  |
| K20 | Do you have a budget or a spending plan that you use to guide your monthly spending? (for example, keeping tabs on your expenses or a plan on how to use your money) | No | 0 | eco_budget |
|  |  | Yes | 1 |  |
|  |  | Don’t know what a budget or spending plan is | 88 |  |
|  |  | Prefer not to respond | 99 |  |
| K21 | Over the past two months, how difficult has it been to track (keep track or keep tab) of how much (amount) you spend on a weekly basis? | Not difficult at all | 0 | eco_track |
|  |  | Somewhat difficult | 1 |  |
|  |  | Very difficult | 2 |  |
|  |  | Extremely difficult (you can’t do it all) | 3 |  |
|  |  | Don’t know | 88 |  |
|  |  | Prefer not to respond | 99 |  |
|  |  | Not applicable | 66 |  |
| K22 | Over the past two months, how difficult has it been to spend on only what you absolutely need and not waste money on things you don’t really need? | Not difficult at all | 0 | eco_manage |
|  |  | Somewhat difficult | 1 |  |
|  |  | Very difficult | 2 |  |
|  |  | Extremely difficult (you can’t do it all) | 3 |  |
|  |  | Don’t know | 88 |  |
|  |  | Prefer not to respond | 99 |  |
|  |  | Not applicable | 66 |  |
| K23 | How good would you like to be at making a budget? (probes: a budget for planning how you spend your money, or a spending plan for pocket money, income, and spending? [Use glass scale] | Not at all good | 0 | eco_goodatbudget |
|  |  | Somewhat good | 1 |  |
|  |  | Very good | 2 |  |
|  |  | Extremely good | 3 |  |
|  |  | Don’t know what a budget or spending plan is | 88 |  |
|  |  | Prefer not to respond | 99 |  |
| K24 | Suppose you receive 20,000 pesos/ NRs 400 / R 200. You can invest any portion of this money in a risky option, which has a 50-50 (%) chance (by coin toss) that the investment will fail or succeed. The rest of the money will be yours to keep. If the investment fails, you lose the amount you invested. If the investment succeeds, you receive 3 times the amount invested. How much would you like to invest? [Enumerator note: Start with an example: If you choose to invest all of the 20,000 pesos/ NRs 400 / R 200, then if the coin comes up heads, you win 60,000 pesos/ NRs 1200 / R 600, and if the coin comes up tails, you win nothing and end up with 0] | 🖎 (in local currency) | | eco_finance |
|  |  | Don’t know | 88 |  |
|  |  | Prefer not to respond | 99 |  |
| K25 | Suppose someone gave you 100,000 pesos, NRs 2000, R 1000 cash, would you share the money with others (e.g., share the money with family, friends, relatives, etc.)? Pick the option that best describes what you would do: | Definitely yes | 1 | eco_share2 |
|  |  | Probably yes | 2 |  |
|  |  | Probably not | 3 |  |
|  |  | Definitely not | 3 |  |
|  |  | Don’t know | 88 |  |
|  |  | Prefer not to respond | 99 |  |

| L. Client Service Receipt Inventory (Adapted) (Beecham & Knapp, 2001)  *[12-month post-baseline assessment only]* | | | | |
| --- | --- | --- | --- | --- |
| Instructions:  *“These next questions are about the services that you might have used over the past few months.*  *Every question is important, please try your best to respond each of them. If you do not want to answer a question, please let us know. Remember that all your answers are confidential”* | | | | |
| L1 | Have you attended the emergency department of a hospital in the past 6 months? | No | 0 | emergency |
|  |  | Yes [Answer L2-L4] | 1 |  |
|  |  | Prefer not to respond | 99 |  |
| L2 | How many times did you attend the emergency department of a hospital in the past 6 months? | 🖎 |  | emergency_times |
| L3 | What was the emergency? | 🖎 |  | emergency_why |
| L4 | Did you, your parents or someone else pay for this? | No | 0 | emergency_pay |
|  |  | Yes [Answer L5] | 1 |  |
|  |  | Prefer not to respond | 99 |  |
| L5 | How much did you, your parents or someone else pay? | 🖎 |  | emergency_cost |
| L6 | Have you been admitted to hospital (i.e. staying at least one night) in the past 6 months? | No | 0 | admitted |
|  |  | Yes [Answer L6-L8] | 1 |  |
|  |  | Prefer not to respond | 99 |  |
| L7 | How many days were you in hospital altogether? | 🖎 |  | admitted_days |
| L8 | What were the reasons for your hospital stay? | Infectious disease (e.g. HIV, malaria, TB) | 1 | admitted_why |
|  |  | Maternal / perinatal condition | 2 |  |
|  |  | Acute condition (e.g. flu, cough) | 3 |  |
|  |  | Injury | 4 |  |
|  |  | Sleep problems | 5 |  |
|  |  | Depression or anxiety | 6 |  |
|  |  | Alcohol problems | 7 |  |
|  |  | Other mental health problems | 8 |  |
|  |  | Other chronic disease (e.g. heart, diabetes) | 9 |  |
|  |  | Other reason [Answer L9] | 77 |  |
|  |  | Don’t know | 88 |  |
|  |  | Prefer not to respond | 99 |  |
| L9 | What other reason were you admitted to hospital? | 🖎 |  | admitted_whyo |
| L10 | Did you, your parents or someone else pay for this? | No | 0 | admitted_pay |
|  |  | Yes [Answer L11] | 1 |  |
|  |  | Prefer not to respond | 99 |  |
| L11 | How much did you, your parents or someone else pay? | 🖎 |  | admitted_cost |
| The following section pertains to service use for physical health | | | | |
| L12 | Have you seen someone in the hospital or in the community for your physical health in the past 3 months? | No | 0 | outpatient_physical |
|  |  | Yes [Answer L13] | 1 |  |
|  |  | Prefer not to respond | 99 |  |
| L13 | Total number of visits in the past 3 months | 🖎 |  | phys_times |
| Visit for physical health: Section repeated by number reported under [phys_times] | | | | |
| L14 | Type of practitioner seen | Traditional healer/spiritualist/herbalist | 1 | phys_type |
|  |  | Specialist doctor | 2 |  |
|  |  | Health professional at school | 3 |  |
|  |  | Community health worker | 4 |  |
|  |  | Nurse/midwife | 5 |  |
|  |  | Pharmacist | 6 |  |
|  |  | General medical doctor | 7 |  |
|  |  | Social worker (Female Community Health Volunteer) [Ne] | 8 |  |
|  |  | Occupational therapist | 9 |  |
|  |  | Physiotherapist | 10 |  |
|  |  | Other health worker | 77 |  |
|  |  | Don't know | 88 |  |
|  |  | Prefer not to respond | 99 |  |
| L15 | Other type of practitioner seen | 🖎 |  | phys_typeo |
| L16 | How long did the visit last (including waiting time)? (in minutes) | 🖎 |  | phys_long |
| L17 | How much time did the practitioner spend with you? (in minutes) | 🖎 |  | phys_timespent |
| L18 | How much did you (or someone else) pay for this, including travel expenditure? | 🖎 |  | phys_cost |
| L19 | How long did it take to travel there and back? (in minutes) | 🖎 |  | phys_travel |
| The following section pertains to service use for mental health | | | | |
| L20 | Have you seen someone in the hospital or in the community for your mental health or wellbeing in the past 3 months? | No | 0 | outpatient_mh |
|  |  | Yes [Answer L21] | 1 |  |
|  |  | Prefer not to respond | 99 |  |
| L21 | Total number of visits in the past 3 months | 🖎 |  | mh_times |
| Visit for mental health: Section repeated by number reported under [mh_times] | | | | |
| L22 | Type of practitioner seen | General medical doctor | 1 | mh_type |
|  |  | Psychiatrist | 2 |  |
|  |  | Psychologist | 3 |  |
|  |  | School/student counsellor | 4 |  |
|  |  | Psychosocial counsellor | 5 |  |
|  |  | Psychiatric nurse | 6 |  |
|  |  | Psychiatric clinical officer | 7 |  |
|  |  | Social worker | 8 |  |
|  |  | Religious/spiritual/traditional healer | 9 |  |
|  |  | Specialist doctor (other than psychiatrist) | 10 |  |
|  |  | Occupational therapist | 11 |  |
|  |  | Other practitioner | 77 |  |
|  |  | Don't know | 88 |  |
|  |  | Prefer not to respond | 99 |  |
| L23 | Other type of practitioner seen | 🖎 |  | mh_typeo |
| L24 | How long did the visit last (including waiting time)? (in minutes) | 🖎 |  | mh_long |
| L25 | How much time did the practitioner spend with you? (in minutes) | 🖎 |  | mh_timespent |
| L26 | How much did you (or someone else) pay for this, including travel expenditure? | 🖎 |  | mh_cost |
| L27 | How long did it take to travel there and back? (in minutes) | 🖎 |  | mh_travel |
| The following section pertains to other services not mentioned above | | | | |
| L28 | Have you used, or had contact with, any other people or services in the past 3 months (not including what you reported above)? | No | 0 | outpatient_other |
|  |  | Yes [Answer L29] | 1 |  |
|  |  | Prefer not to respond | 99 |  |
| L29 | Total number of visits in the past 3 months | 🖎 |  | other_times |
| Other visit: Section repeated by number reported under [other_times] | | | | |
| L30 | Type of person seen/service sought | Child or youth club | 1 | other_type |
|  |  | Fortune teller | 2 |  |
|  |  | Motivational speaker | 3 |  |
|  |  | Debt advisor | 4 |  |
|  |  | School advsor/parents teacher association (PTA) | 5 |  |
|  |  | Addiction support | 6 |  |
|  |  | Police personnel | 7 |  |
|  |  | Prison or arrest unit | 8 |  |
|  |  | Life counsellor, coach or mentor | 9 |  |
|  |  | Other health worker | 77 |  |
|  |  | Don't know | 88 |  |
|  |  | Prefer not to respond | 99 |  |
| L31 | Other type of practitioner seen | 🖎 |  | other _typeo |
| L32 | How long did the visit last (including waiting time)? (in minutes) | 🖎 |  | other _long |
| L33 | How much did you (or someone else) pay for this, including travel expenditure? | 🖎 |  | other_cost |
| L34 | How long did it take to travel there and back? (in minutes) | 🖎 |  | other_travel |
| End of repeat section | | | | |
| L35 | Have you missed school or work in the last month, for example, because you did not feel like going or something else came up? | No | 0 | schoolmissed |
|  |  | Yes [Answer L35-L37] | 1 |  |
|  |  | Prefer not to respond | 99 |  |
| L35 | How many days did you miss? | 🖎 |  | schoolmissed_days |
| L36 | What type of activity did you miss? | Education | 1 | schoolmissed_activity |
|  |  | Paid work | 2 |  |
|  |  | Unpaid work | 3 |  |
|  |  | Other | 77 |  |
|  |  | Don't know | 88 |  |
|  |  | Prefer not to respond | 99 |  |
| L37 | How much income (money) did you lose by missing this activity? | 🖎 |  | schoolmissed_cost |

| L. The Child and Youth Resilience Measure (CYRM) – 17  (Jefferies et al., 2019)  (Nepal only) | | | | |
| --- | --- | --- | --- | --- |
| Instructions:  *“For anyone to stay healthy they will need different resources (For example, education, guardians, friends, neighbours, school, community, tradition, etc). We will be asking 17 questions based on these which will be regarding the people around you, about the importance of education, about the behaviour that you see in different places, and regarding your guardians, family, friends, neighbours, school, and community. Every question will have 5 responses. Among these responses, select one option that matches your experience. Every question is important, please try your best to respond each of them. If you do not want to answer a question, please let us know. Remember that all your answers are confidential.”* | | | | |
| L1 | How much can you stay together in harmony with people around you (For example, friends, relatives, neighbors)?  (Staying together in harmony would mean helping each other, eating, playing, or working together, etc.) | Not at all | 1 | cyrm_1 |
|  |  | Just a little | 2 |  |
|  |  | To some extent | 3 |  |
|  |  | Quite a lot | 4 |  |
|  |  | A lot | 5 |  |
|  |  | Prefer not to respond | 99 |  |
| L2 | How important is education for you? | Not at all | 1 | cyrm_2 |
|  |  | Just a little | 2 |  |
|  |  | To some extent | 3 |  |
|  |  | Quite a lot | 4 |  |
|  |  | A lot | 5 |  |
|  |  | Prefer not to respond | 99 |  |
| L3 | How much do you know how to behave in different places (For example, school, temple, house, etc.)? | Not at all | 1 | cyrm_3 |
|  |  | Just a little | 2 |  |
|  |  | To some extent | 3 |  |
|  |  | Quite a lot | 4 |  |
|  |  | A lot | 5 |  |
|  |  | Prefer not to respond | 99 |  |
| L4 | How much do you feel like your parents really look after you? | Not at all | 1 | cyrm_4 |
|  |  | Just a little | 2 |  |
|  |  | To some extent | 3 |  |
|  |  | Quite a lot | 4 |  |
|  |  | A lot | 5 |  |
|  |  | Prefer not to respond | 99 |  |
| L5 | How much do you feel like your parents know a lot about you? (For example, who your friends are, what you want to do, etc) | Not at all | 1 | cyrm_5 |
|  |  | Just a little | 2 |  |
|  |  | To some extent | 3 |  |
|  |  | Quite a lot | 4 |  |
|  |  | A lot | 5 |  |
|  |  | Prefer not to respond | 99 |  |
| L6 | How much do you feel like there’s a lot of food at home for you to eat when you are hungry? | Not at all | 1 | cyrm_6 |
|  |  | Just a little | 2 |  |
|  |  | To some extent | 3 |  |
|  |  | Quite a lot | 4 |  |
|  |  | A lot | 5 |  |
|  |  | Prefer not to respond | 99 |  |
| L7 | How much do other people like spending time with you? (Other people like friends, siblings, etc) | Not at all | 1 | cyrm_7 |
|  |  | Just a little | 2 |  |
|  |  | To some extent | 3 |  |
|  |  | Quite a lot | 4 |  |
|  |  | A lot | 5 |  |
|  |  | Prefer not to respond | 99 |  |
| L8 | How much do you talk about your feelings (for example, sadness, happiness, fear, worries, anger, etc) with your family? | Not at all | 1 | cyrm_8 |
|  |  | Just a little | 2 |  |
|  |  | To some extent | 3 |  |
|  |  | Quite a lot | 4 |  |
|  |  | A lot | 5 |  |
|  |  | Prefer not to respond | 99 |  |
| L9 | How much do you feel like your friends help you? | Not at all | 1 | cyrm_9 |
|  |  | Just a little | 2 |  |
|  |  | To some extent | 3 |  |
|  |  | Quite a lot | 4 |  |
|  |  | A lot | 5 |  |
|  |  | Prefer not to respond | 99 |  |
| L10 | How much do you feel like the school where you study is like your own? (For example, teachers teaching and guiding you in a friendly manner, getting along together with friends, helping each other, etc) | Not at all | 1 | cyrm_10 |
|  |  | Just a little | 2 |  |
|  |  | To some extent | 3 |  |
|  |  | Quite a lot | 4 |  |
|  |  | A lot | 5 |  |
|  |  | Prefer not to respond | 99 |  |
| L11 | How much do you feel like your family will support you when you are in a difficult situation (For example, when you are sick, when you do something wrong, etc)? | Not at all | 1 | cyrm_11 |
|  |  | Just a little | 2 |  |
|  |  | To some extent | 3 |  |
|  |  | Quite a lot | 4 |  |
|  |  | A lot | 5 |  |
|  |  | Prefer not to respond | 99 |  |
| L12 | How much do you feel like your friends will help you when you are in a difficult situation (For example, when you are sick, when you do something wrong, etc)? | Not at all | 1 | cyrm_12 |
|  |  | Just a little | 2 |  |
|  |  | To some extent | 3 |  |
|  |  | Quite a lot | 4 |  |
|  |  | A lot | 5 |  |
|  |  | Prefer not to respond | 99 |  |
| L13 | How much do you feel like the people in your community show good behavior towards you? (Good behavior as in loving those younger than you, never lying to you, never stealing from you, talking nicely to you, etc) | Not at all | 1 | cyrm_13 |
|  |  | Just a little | 2 |  |
|  |  | To some extent | 3 |  |
|  |  | Quite a lot | 4 |  |
|  |  | A lot | 5 |  |
|  |  | Prefer not to respond | 99 |  |
| L14 | How much have you gotten the opportunity to prove yourself to others by doing tasks according to your age by yourself? (For example, cook food, wash dishes, clean house, make your bed) | Not at all | 1 | cyrm_14 |
|  |  | Just a little | 2 |  |
|  |  | To some extent | 3 |  |
|  |  | Quite a lot | 4 |  |
|  |  | A lot | 5 |  |
|  |  | Prefer not to respond | 99 |  |
| L15 | How safe do you feel when you are with your family? (Feeling safe as in never feeling scared when you are with your family, being able to mingle around with others, others not being able to beat you, etc) | Not at all | 1 | cyrm_15 |
|  |  | Just a little | 2 |  |
|  |  | To some extent | 3 |  |
|  |  | Quite a lot | 4 |  |
|  |  | A lot | 5 |  |
|  |  | Prefer not to respond | 99 |  |
| L16 | How much do you feel like you have had the opportunity to  learn the skills that will be useful in the future? (Skills such as  cooking food, helping others, stitching clothes, working in mobile phone store, etc.) | Not at all | 1 | cyrm_16 |
|  |  | Just a little | 2 |  |
|  |  | To some extent | 3 |  |
|  |  | Quite a lot | 4 |  |
|  |  | A lot | 5 |  |
|  |  | Prefer not to respond | 99 |  |
| L17 | How much do you enjoy the festivals and traditions celebrated by your family? | Not at all | 1 | cyrm_17 |
|  |  | Just a little | 2 |  |
|  |  | To some extent | 3 |  |
|  |  | Quite a lot | 4 |  |
|  |  | A lot | 5 |  |
|  |  | Prefer not to respond | 99 |  |

| M. Internal Resilience (RRM) 10 items  (Jefferies et al., 2022)  (Nepal only) | | | | |
| --- | --- | --- | --- | --- |
| Instructions:  *“There might be different kinds of problems in a person’s life for example, problems at school, family, with friends or problems related to one's own health. To be able to solve those problems, people need to have the willpower, flexibility according to situations and self confidence. Now I will ask you 10 questions related to these things and you have to answer among the 5 options given here based on your experience. Every question is important, please try your best to respond each of them. If you do not want to answer a question, please let us know. Remember that all your answers are confidential.”* | | | | |
| M1 | How much do you believe in yourself? | Not at all | 1 | rrm_1 |
|  |  | Just a little | 2 |  |
|  |  | To some extent | 3 |  |
|  |  | Quite a lot | 4 |  |
|  |  | A lot | 5 |  |
|  |  | Prefer not to respond | 99 |  |
| M2 | How much can you adjust in difficult situations? (For example, being able to mingle around when you go to new place or school, being able to reconcile with friends even after fighting with them, etc) | Not at all | 1 | rrm_2 |
|  |  | Just a little | 2 |  |
|  |  | To some extent | 3 |  |
|  |  | Quite a lot | 4 |  |
|  |  | A lot | 5 |  |
|  |  | Prefer not to respond | 99 |  |
| M3 | How much are you able to look for solutions when you are faced with a problem? | Not at all | 1 | rrm_3 |
|  |  | Just a little | 2 |  |
|  |  | To some extent | 3 |  |
|  |  | Quite a lot | 4 |  |
|  |  | A lot | 5 |  |
|  |  | Prefer not to respond | 99 |  |
| M4 | How much are you able to proceed forward and not give up even when you are experiencing difficulty? | Not at all | 1 | rrm_4 |
|  |  | Just a little | 2 |  |
|  |  | To some extent | 3 |  |
|  |  | Quite a lot | 4 |  |
|  |  | A lot | 5 |  |
|  |  | Prefer not to respond | 99 |  |
| M5 | How well are you able to deal with having more tasks than usual? (Tasks such as tuition classes, playing, homework, cooking food, etc) | Not at all | 1 | rrm_5 |
|  |  | Just a little | 2 |  |
|  |  | To some extent | 3 |  |
|  |  | Quite a lot | 4 |  |
|  |  | A lot | 5 |  |
|  |  | Prefer not to respond | 99 |  |
| M6 | How much are you hopeful about your future even when there are difficulties in your life? | Not at all | 1 | rrm_6 |
|  |  | Just a little | 2 |  |
|  |  | To some extent | 3 |  |
|  |  | Quite a lot | 4 |  |
|  |  | A lot | 5 |  |
|  |  | Prefer not to respond | 99 |  |
| M7 | How much are you able to control your feelings? (For example being able to control yourself when you are feeling happy, sad, upset, jealous, etc ) | Not at all | 1 | rrm_7 |
|  |  | Just a little | 2 |  |
|  |  | To some extent | 3 |  |
|  |  | Quite a lot | 4 |  |
|  |  | A lot | 5 |  |
|  |  | Prefer not to respond | 99 |  |
| M8 | How proud do you feel of yourself when you are able to accomplish the things that you wanted to? (For example, when you are successful in your studies, when you win in sports, when you are able to make new friends, when you cook a delicious meal, etc) | Not at all | 1 | rrm_8 |
|  |  | Just a little | 2 |  |
|  |  | To some extent | 3 |  |
|  |  | Quite a lot | 4 |  |
|  |  | A lot | 5 |  |
|  |  | Prefer not to respond | 99 |  |
| M9 | When you have difficulty, how much do you try to face those situations? | Not at all | 1 | rrm_9 |
|  |  | Just a little | 2 |  |
|  |  | To some extent | 3 |  |
|  |  | Quite a lot | 4 |  |
|  |  | A lot | 5 |  |
|  |  | Prefer not to respond | 99 |  |
| M10 | How much are you able to understand the purpose of your life? | Not at all | 1 | rrm_10 |
|  |  | Just a little | 2 |  |
|  |  | To some extent | 3 |  |
|  |  | Quite a lot | 4 |  |
|  |  | A lot | 5 |  |
|  |  | Prefer not to respond | 99 |  |

| N. Anthropometric measures | | | |
| --- | --- | --- | --- |
| N1 | What is the height of the participant? (in meters) | 🖎 | height |
| N2 | What is the participant’s weight (in kilograms) | 🖎 | weight |
| N4 | Participant BMI [calculated] |  | bmi |

| O. Contamination | | | | |
| --- | --- | --- | --- | --- |
| O1 | Have you heard about this study from other family members, friends or other people? | No | 0 | aware |
|  |  | Yes [Answer O2] | 1 |  |
|  |  | Don’t know | 88 |  |
|  |  | Prefer not to respond | 99 |  |
| O2 | Can you tell me a bit more about what they said? | 🖎 | | aware_describe |

| P. Blinding | | | | |
| --- | --- | --- | --- | --- |
| P1 | [To fieldworker] Please indicate which intervention arm you think the participant is allocated to. | Control | 0 | blinding |
|  |  | Self-regulation intervention | 1 |  |
|  |  | Economic intervention | 2 |  |
|  |  | Combined (self-regulation and economic) intervention | 3 |  |

References

Angulo, R., Díaz, Y., & Pardo, R. (2016). The Colombian multidimensional poverty index: Measuring poverty in a public policy context. *Social Indicators Research*, *127*, 1-38.

Ashraf, N., Bau, N., Low, C., & McGinn, K. (2020). Negotiating a better future: How interpersonal skills facilitate intergenerational investment. *The quarterly journal of economics*, *135*(2), 1095-1151.

Beecham, J., & Knapp, M. (2001). Costing psychiatric interventions. In G. Thornicroft (Ed.), *Measuring mental health needs* (2nd ed., pp. 200-224). Gaskell.

Bernstein, D. P., Stein, J. A., Newcomb, M. D., Walker, E., Pogge, D., Ahluvalia, T., Stokes, J., Handelsman, L., Medrano, M., & Desmond, D. (2003). Development and validation of a brief screening version of the Childhood Trauma Questionnaire. *Child Abuse and Neglect*, *27*(2), 169-190.

Bruhn, M., Garber, G., Koyama, S., & Zia, B. (2022). The Long-Term Impact of High School Financial Education.

Burkey, M. D., Adhikari, R. P., Ghimire, L., Kohrt, B. A., Wissow, L. S., Luitel, N. P., Haroz, E. E., & Jordans, M. J. (2018). Validation of a cross-cultural instrument for child behavior problems: the Disruptive Behavior International Scale–Nepal version. *BMC psychology*, *6*(1), 1-13.

Carvajal-Velez, L., Ahs, J. W., Requejo, J. H., Kieling, C., Lundin, A., Kumar, M., Luitel, N. P., Marlow, M., Skeen, S., & Tomlinson, M. (2023). Measurement of mental health among adolescents at the population level: A multicountry protocol for adaptation and validation of mental health measures. *Journal of Adolescent Health*, *72*(1), S27-S33.

Central Bureau of Statistics Nepal. (2021). *Nepal Multidimensional Poverty Index: Analysis towards action*. N. P. C. Government of Nepal.

Essau, C. A., Sasagawa, S., & Frick, P. J. (2006). Psychometric properties of the Alabama parenting questionnaire. *Journal of Child and Family Studies*, *15*(5), 595-614.

International Labour Organization. (2022). *Fundamentals*. ILO.

Jefferies, P., McGarrigle, L., & Ungar, M. (2019). The CYRM-R: A Rasch-validated revision of the child and youth resilience measure. *Journal of Evidence-Based Social Work*, *16*(1), 70-92.

Jefferies, P., Vanstone, R., & Ungar, M. (2022). The rugged resilience measure: Development and preliminary validation of a brief measure of personal resilience. *Applied Research in Quality of Life*, *17*(2), 985-1000.

Jensen, R. (2010). The (perceived) returns to education and the demand for schooling. *The quarterly journal of economics*, *125*(2), 515-548.

Johnson, J. G., Harris, E. S., Spitzer, R. L., & Williams, J. B. (2002). The patient health questionnaire for adolescents: validation of an instrument for the assessment of mental disorders among adolescent primary care patients. *Journal of Adolescent Health*, *30*(3), 196-204.

Kaufman, E. A., Xia, M., Fosco, G., Yaptangco, M., Skidmore, C. R., & Crowell, S. E. (2016). The Difficulties in Emotion Regulation Scale Short Form (DERS-SF): Validation and replication in adolescent and adult samples. *Journal of Psychopathology and Behavioral Assessment*, *38*(3), 443-455.

Kroenke, K., & Spitzer, R. L. (2002). The PHQ-9: a new depression diagnostic and severity measure. *Psychiatric Annals*, *32*(9), 1-7.

Lejuez, C. W., Read, J. P., Kahler, C. W., Richards, J. B., Ramsey, S. E., Stuart, G. L., Strong, D. R., & Brown, R. A. (2002). Evaluation of a behavioral measure of risk taking: the Balloon Analogue Risk Task (BART). *Journal of Experimental Psychology: Applied*, *8*(2), 75.

Manos, R. C., Kanter, J. W., & Luo, W. (2011). The behavioral activation for depression scale–short form: development and validation. *Behavior Therapy*, *42*(4), 726-739.

Richards, J., Zhang, L., Mitchell, S. and Wit, H. (1999). Delay or probability discounting in a model of impulsive behavior: Effect of alcohol. Journal of the experimental analysis of behavior. 71. 121-43.

Rocha, T. B.-M., Fisher, H. L., Caye, A., Anselmi, L., Arseneault, L., Barros, F. C., Caspi, A., Danese, A., Goncalves, H., & Harrington, H. L. (2021). Identifying adolescents at risk for depression: A prediction score performance in cohorts based in 3 different continents. *Journal of the American Academy of Child and Adolescent Psychiatry*, *60*(2), 262-273.

Schaefer, E., & Edgerton, M. (1985). Parental modernity scale of child rearing and educational beliefs. In.

Schulz, K. P., Fan, J., Magidina, O., Marks, D. J., Hahn, B., & Halperin, J. M. (2007). Does the emotional go/no-go task really measure behavioral inhibition? Convergence with measures on a non-emotional analog. *Arch Clin Neuropsychol*, *22*(2), 151-160. <https://doi.org/10.1016/j.acn.2006.12.001>

Snyder, C. R., Hoza, B., Pelham, W. E., Rapoff, M., Ware, L., Danovsky, M., Highberger, L., Ribinstein, H., & Stahl, K. J. (1997). The development and validation of the Children’s Hope Scale. *Journal of Pediatric Psychology*, *22*(3), 399-421.

Spitzer, R. L., Kroenke, K., Williams, J. B., & Löwe, B. (2006). A brief measure for assessing generalized anxiety disorder: the GAD-7. *Archives of Internal Medicine*, *166*(10), 1092-1097.

Statistics South Africa. (2014). *The South African MPI: Creating a multidimensional poverty index using census data*.

Tottenham, N., Hare, T. A., Millner, A., Gilhooly, T., Zevin, J. D., and Casey, B. J. (2011). Elevated amygdala response to faces following early deprivation. Developmental Science, 14, 190–204.

Vazsonyi, A. T., Jiskrova, G. K., & Ksinan, A. J. (2018). Sleep, low self-control, and deviance: Direct and indirect links across immigrant groups and socioeconomic strata. *Journal of Adolescence*, *68*, 40-49.

Watts, A. L., Smith, G. T., Barch, D. M., & Sher, K. J. (2020). Factor structure, measurement and structural invariance, and external validity of an abbreviated youth version of the UPPS-P Impulsive Behavior Scale. *Psychological Assessment*, *32*(4), 336.
