# Supplementary material for: Combining self-regulation and poverty reduction to prevent depression and anxiety among adolescents experiencing multi-dimensional poverty in Colombia, Nepal and South Africa: study protocol of a pilot 4-arm cluster randomised controlled trial
Source: Pilot Feasibility Stud. 2026 Jun 8;12:113. doi: 10.1186/s40814-026-01858-y (PMC13465900; doi:10.1186/s40814-026-01858-y)
Supplement: Supplementary file 4 [file 40814_2026_1858_MOESM4_ESM.docx]

**Caregiver questionnaire (version 6 – 10 October 2024)**

| A. Introduction | | | | |
| --- | --- | --- | --- | --- |
| Instructions: “*Every question is important, please try your best to respond each of them. If you do not want to answer a question, please let us know. Remember that all your answers are confidential.*” | | | | |
| A1 | Date | 🖎 |  | cgdate |
| A2 | Time | 🖎 |  | cgstarttime |
| A3 | Which timepoint is this? | Baseline |  | timepoint |
|  |  | Post-intervention |  |  |
|  |  | 12-month post baseline |  |  |
|  |  | 18-month post baseline |  |  |
| A4 | Which fieldworker is completing this questionnaire | Johana Gallego [Col] | 11 | cgfw |
|  |  | Patricia Muñoz [Col] | 12 |  |
|  |  | Ysandra Tibisay Romero [Col] | 13 |  |
|  |  | Angélica Rojas [Col] | 14 |  |
|  |  | Shirley González [Col] | 15 |  |
|  |  | Claudia Bello [Col] | 16 |  |
|  |  | Mirna Mendez [Col] | 17 |  |
|  |  | Henis Molina [Col] | 18 |  |
|  |  | Aditi Ghimire [Ne] | 20 |  |
|  |  | Anushka Thapa [Ne] | 21 |  |
|  |  | Dipa Giri [Ne] | 22 |  |
|  |  | Neeru Yadav [Ne] | 23 |  |
|  |  | Bibek Prajuli [Ne] | 24 |  |
|  |  | Jushan Shrestha [Ne] | 25 |  |
|  |  | Dikshya Chand [Ne] | 26 |  |
|  |  | Sabina Karki [Ne] | 27 |  |
|  |  | Sabitra Dahal [Ne] | 28 |  |
|  |  | Sushan Ghimire [Ne] | 29 |  |
|  |  | Subina Shrestha [Ne] | 30 |  |
|  |  | Megan Pool [SA] | 31 |  |
|  |  | Xola Jiyana [SA] | 32 |  |
|  |  | Tarshlyn Herandien [SA] | 35 |  |
|  |  | Zoleka Maqina [SA] | 36 |  |
|  |  | Bulelani Velaphi [SA] | 37 |  |
|  |  | Zimasa Ranyana [SA] | 38 |  |
|  |  | Iona Gutuza [SA] | 39 |  |
|  |  | Nontobeko Mdudu [SA] | 40 |  |
|  |  | Thozama H-Kasozi [SA] | 41 |  |
|  |  | Veliswa Cebisa [SA] | 42 |  |
|  |  | Azola Sibonda [SA] | 43 |  |
|  |  | Noma Moshani [SA] | 44 |  |
|  |  | Other [Answer A5] | 77 |  |
| A5 | What other fieldworker? | 🖎 | | cgfwo |
| A6 | Please enter the participants PID | 🖎 | | cgid |
| A7 | Please enter the participant’s PID again | 🖎 | | cgid2 |
| A8 | Correct 4-digit PID |  | | id_final |
| A9 | Full PID for cross-country analysis |  | | cgpid |
| A10 | Where is the assessment conducted? | At home | 1 | cglocation |
|  |  | At school | 2 |  |
|  |  | Foundation [Col] | 3 |  |
|  |  | Other location [Answer A11] | 77 |  |
| A11 | Please specify other location | 🖎 | | cglocationo |
| A12 | *[Baseline only]*  What is the name of the school? | C. Santa Barbara [Col] | 1 | cgschool |
|  |  | C. La Arabia [Col] | 2 |  |
|  |  | C. La Estancia [Col] | 3 |  |
|  |  | C. Rogelio Salmona [Col] | 4 |  |
|  |  | C. San Jose de Usme [Col] | 5 |  |
|  |  | C. Chuniza Fámaco [Col] | 6 |  |
|  |  | C. Gabriel Garcia Marquez [Col] | 7 |  |
|  |  | C. Fabio Lozano Simonellli [Col] | 8 |  |
|  |  | Budhanilkantha [Ne] | 4 |  |
|  |  | Dhumra Baraha [Ne] | 5 |  |
|  |  | Gram Shikshya [Ne] | 6 |  |
|  |  | Jana Uddhar [Ne] | 7 |  |
|  |  | Mahankal Janajagriti [Ne] | 8 |  |
|  |  | Naulin [Ne] | 9 |  |
|  |  | Pashupati Mitra [Ne] | 10 |  |
|  |  | Shree Ganesh [Ne] | 11 |  |
|  |  | Budhanilkantha [Ne] | 12 |  |
|  |  | Dhumra Baraha [Ne] | 13 |  |
|  |  | Gram Shikshya [Ne] | 14 |  |
|  |  | Jana Uddhar [Ne] | 15 |  |
|  |  | Mahankal Janajagriti [Ne] | 16 |  |
|  |  | Centre of Science and Technology [SA] | 17 |  |
|  |  | Esangweni Secondary School [SA] | 18 |  |
|  |  | Houtbaai Sekonder [SA] | 19 |  |
|  |  | Joe Slovo Secondary School [SA] | 20 |  |
|  |  | Kwamfundo Secondary School [SA] | 21 |  |
|  |  | Paramount High School [SA] | 22 |  |
|  |  | Silikamva High School [SA] | 23 |  |
|  |  | Usasazo Secondary School [SA] | 24 |  |

| B. Demographics | | | | | |
| --- | --- | --- | --- | --- | --- |
| Instructions: “*Every question is important, please try your best to respond each of them. If you do not want to answer a question, please let us know. Remember that all your answers are confidential.*” | | | | | |
| B1 | *[Baseline only]* What is your preferred gender identity? | Male | 0 | cg_gender | |
|  |  | Female | 1 |  |  |
|  |  | Non-binary | 2 |  |  |
|  |  | Other [Answer B2] | 77 |  |  |
|  |  | Prefer not respond | 99 |  |  |
| B2 | Please specify what other gender | 🖎 |  | cg_gendero | |
| B3 | *[Baseline only]* How old are you? | 🖎 | | | cg_age |
| B4 | *[Baseline only]* What is your ethnicity/population group (caste etc.)? | None | 0 | cg_ethnicity | |
|  |  | Gypsy or Roma [Col] | 1 |  |  |
|  |  | Indigenous [Col] | 2 |  |  |
|  |  | Negro, Mulatto, Afro-descendant, Afro-Colombian [Col] | 3 |  |  |
|  |  | Palenquero of San Basilio [Col] | 4 |  |  |
|  |  | Raizal of the Archipelago of San Andres, Providencia and Santa Catalina [Col] | 5 |  |  |
|  |  | Brahmin or Chhetri [Ne] | 6 |  |  |
|  |  | Dalit [Ne] | 7 |  |  |
|  |  | Janajati [Ne] | 8 |  |  |
|  |  | Madhesi [Ne] | 9 |  |  |
|  |  | Muslim [Ne] | 10 |  |  |
|  |  | Black [SA] | 11 |  |  |
|  |  | Coloured [SA] | 12 |  |  |
|  |  | Indian [SA] | 13 |  |  |
|  |  | White [SA] | 14 |  |  |
|  |  | Other | 77 |  |  |
|  |  | Don't know | 88 |  |  |
| B5 | Please specify what other ethnicity | 🖎 |  | cg_ethnicityo | |
| B6 | *[For South Africa only]*  What is your preferred spoken language? | English | 3 | cg_language | |
|  |  | isiXhosa | 4 |  |  |
|  |  | Afrikaans | 5 |  |  |
|  |  | Other [Answer B7] | 77 |  |  |
|  |  | Prefer not to respond | 99 |  |  |
| B7 | Please specify what other language | 🖎 | | | cg_languageo |
| B8 | What is your marital status? | Single | 1 | cg_marital_status | |
|  |  | Married | 2 |  |  |
|  |  | Divorced | 3 |  |  |
|  |  | Widowed | 4 |  |  |
|  |  | Voluntary union [Col] | 5 |  |  |
|  |  | Separated | 6 |  |  |
|  |  | Other [Answer B9] | 77 |  |  |
|  |  | I don’t know | 88 |  |  |
|  |  | Prefer not to answer | 99 |  |  |
| B9 | What is your other marital status? | 🖎 | | | cg_maritalo |

| C. Social and Economic Measures (Caregivers) | | | | | | | |
| --- | --- | --- | --- | --- | --- | --- | --- |
| Instructions: “*Every question is important, please try your best to respond each of them. If you do not want to answer a question, please let us know. Remember that all your answers are confidential.*” | | | | | | | |
| C1 | *[Baseline only]*  For how many years has your family been living in this village/town/city? | 🖎 | | | | migration | |
| C2 | *[Baseline only]*  Did your family move to this village/town/city from elsewhere? | No [Go to C8] | 0 | | | migrate_from | |
|  |  | Yes, from within the district [Go to C4] | 1 | | |  |  |
|  |  | Yes, from outside the district | 2 | | |  |  |
|  |  | Yes, from outside the country | 3 | | |  |  |
|  |  | Don’t know [Go to C8] | 88 | | |  |  |
|  |  | Prefer not to respond [Go to C8] | 99 | | |  |  |
| C3 | Please specify which district/country | 🖎 |  | | | origin_region | |
| C4 | What is the main reason you left your earlier place of residence? | Armed conflict [Answer C5] | 1 | | | reasons_migration | |
|  |  | Crop damaged | 2 | | |  |  |
|  |  | Local crime [Answer C5] | 3 | | |  |  |
|  |  | Natural disaster [Answer C5] | 4 | | |  |  |
|  |  | Economic conditions | 5 | | |  |  |
|  |  | Better job opportunities | 6 | | |  |  |
|  |  | Better educational services | 7 | | |  |  |
|  |  | Better infrastructure or health services | 8 | | |  |  |
|  |  | Health problems | 9 | | |  |  |
|  |  | Marriage | 10 | | |  |  |
|  |  | Family matters | 11 | | |  |  |
|  |  | Others | 77 | | |  |  |
| C5 | *[Baseline only]*  How old were you when you were displaced? | 🖎 | | | | displaced_age | |
|  |  | Don’t know | 88 | | |  |  |
|  |  | Prefer not to respond | 99 | | |  |  |
| C6 | *[Follow-up only]*  Did you and your family move in the past 6 months? | No | 0 | | | cg_moved | |
|  |  | Yes [Answer C7] | 1 | | |  |  |
|  |  | Prefer not to respond | 99 | | |  |  |
| C7 | *[Follow-up only]*  For how many months has your family been living in this village/town/city? | 🖎 | | | | migration_6m | |
| C8 | Have you experienced any serious difficult life events in the past 6 months that have affected you and your family? | No | 0 | | | cg_life_events | |
|  |  | Yes [Answer C9] | 1 | | |  |  |
|  |  | Prefer not to respond | 99 | | |  |  |
| C9 | Which life events? [Select all that apply] | Death | 1 | | | cg_event_ | |
|  |  | Serious illness | 2 | | |  |  |
|  |  | Job loss | 3 | | |  |  |
|  |  | Decrease in cash and goods from family | 4 | | |  |  |
|  |  | Decrease in government grant | 5 | | |  |  |
|  |  | Destruction / theft of household property | 6 | | |  |  |
|  |  | Other [Answer C10] | 77 | | |  |  |
|  |  | Don't know | 88 | | |  |  |
|  |  | Prefer not to respond | 99 | | |  |  |
| C10 | Please specify which other difficult life event | 🖎 |  | | | cg_life_eventso | |
| C11 | Have there been any major stressors in the past 6 months that have affected your neighbourhood or community? | No | 0 | | | cg_stressor | |
|  |  | Yes [Answer C12] | 1 | | |  |  |
|  |  | Prefer not to respond | 99 | | |  |  |
| C12 | Please select all that apply: | Floods | 1 | | | cg_stressor_which | |
|  |  | Landslides/mudslides (e.g. home washing away) | 2 | | |  |  |
|  |  | Other natural disasters (wildfire, tornado etc.) | 3 | | |  |  |
|  |  | Violence in the community (e.g. community assaults, burning of tyres, fights resulting in death, violent fights, witnessing a violent event such as witnessing someone getting killed, stoning cars, looting shops, burning buildings, street violence, and gang violence) | 4 | | |  |  |
|  |  | Political protests | 5 | | |  |  |
|  |  | Major car accident in which multiple people were killed (car, bus, train etc.) | 6 | | |  |  |
|  |  | Major fires with multiple buildings destroyed | 7 | | |  |  |
|  |  | Disease outbreak leading to closure of schools | 8 | | |  |  |
|  |  | Other [Answer C13] | 77 | | |  |  |
|  |  | Don’t know | 88 | | |  |  |
|  |  | Prefer not to respond | 99 | | |  |  |
| C13 | What other major stressors? | 🖎 | | | | cg_stressor_whicho | |
| C14 | How much have these experiences affected you? | Not at all | | | 0 | cg_stressor_effect | |
|  |  | Somewhat/a little | | | 1 |  |  |
|  |  | Very much | | | 2 |  |  |
|  |  | Extremely | | | 3 |  |  |
|  |  | Don’t know | | | 88 |  |  |
|  |  | Prefer not to answer | | | 99 |  |  |
| C15 | During the last 3 months, did this household receive or be a beneficiary of any of the following programmes? | None | | | 0 | programme | |
|  |  | Familias en Acción [Col] | | | 1 |  |  |
|  |  | Programa para adultos mayores [Col] | | | 2 |  |  |
|  |  | Ingreso Solidario [Col] | | | 3 |  |  |
|  |  | Ingreso Mínimo Garantizado [Col] | | | 4 |  |  |
|  |  | Programam de Alimentación Escolar [Col] | | | 18 |  |  |
|  |  | Comedores Comunitarios [Col] | | | 19 |  |  |
|  |  | Subsidio de transmilenio/SITP [Col] | | | 20 |  |  |
|  |  | SENA [Col] | | | 21 |  |  |
|  |  | Red Juntos [Col] | | | 22 |  |  |
|  |  | ICBF [Col] | | | 23 |  |  |
|  |  | Subsidio al desempleo [Col] | | | 24 |  |  |
|  |  | Caja de compensación (subsidios/préstamos) [Col] | | | 25 |  |  |
|  |  | Caja de compensación (salud/recreación) [Col] | | | 26 |  |  |
|  |  | Ayudas para emergencias [Col] | | | 27 |  |  |
|  |  | Ayudas para desplazados [Col] | | | 28 |  |  |
|  |  | Subsidio de vivienda [Col] | | | 29 |  |  |
|  |  | Senior citizen's allowance [Ne] | | | 5 |  |  |
|  |  | Deprived citizen's allowance [Ne] | | | 6 |  |  |
|  |  | Disabled and destitute allowance [Ne] | | | 7 |  |  |
|  |  | Single woman's allowance [Ne] | | | 8 |  |  |
|  |  | Disability allowance [Ne] | | | 9 |  |  |
|  |  | Child nutrition allowance [Ne] | | | 10 |  |  |
|  |  | Endangered indigenous people allowance [Ne] | | | 11 |  |  |
|  |  | Self-care allowance [Ne] | | | 12 |  |  |
|  |  | Child support grant [SA] | | | 13 |  |  |
|  |  | Foster grant [SA] | | | 14 |  |  |
|  |  | Government old age/pension [SA] | | | 15 |  |  |
|  |  | Disability grant [SA] | | | 16 |  |  |
|  |  | Social relief grant [SA] | | | 17 |  |  |
|  |  | Other | | | 77 |  |  |
|  |  | Don’t know | | | 88 |  |  |
|  |  | Prefer not to answer | | | 99 |  |  |
| C16 | [Nepal and South Africa] What is the total household income of the household? (in local currency) | 🖎 | | |  | hhincome | |
|  |  | I don’t know | | | 88 |  |  |
|  |  | Prefer not to answer | | | 99 |  |  |
| C17 | [Colombia only] What is the total household income of the household? (in local currency) | Less than 700,000 | | | 1 | hhincome_col | |
|  |  | 700,001 - 1,100,000 | | | 2 |  |  |
|  |  | 1,100,001 – 1,500,000 | | | 3 |  |  |
|  |  | 1,500,001 – 2,000,000 | | | 4 |  |  |
|  |  | 2,000,001 – 2,500,000 | | | 5 |  |  |
|  |  | More than 2,500,001 | | | 6 |  |  |
|  |  | Don’t know | | | 88 |  |  |
|  |  | Prefer not to answer | | | 99 |  |  |
| C18 | Who among the household roster is the main breadwinner? | Self | | | 1 | breadwinner | |
|  |  | Spouse/partner | | | 2 |  |  |
|  |  | Other relative or household member | | | 3 |  |  |
|  |  | Other [Answer C19] | | | 77 |  |  |
|  |  | I don’t know | | | 88 |  |  |
|  |  | Prefer not to answer | | | 99 |  |  |
| C19 | Who else is the main breadwinner? | 🖎 | | |  | breadwinnero | |
| C20 | How much are the monthly earnings of the main breadwinner? | 0 - 700,000 [Col] | | | 1 | bw_earnings | |
|  |  | 700,001 - 1,100,000 [Col] | | | 2 |  |  |
|  |  | 1,100,001 - 1,500,000 [Col] | | | 3 |  |  |
|  |  | 1,500,001 - 2,000,000 [Col] | | | 4 |  |  |
|  |  | 2,000,001 - 2,500,000 [Col] | | | 5 |  |  |
|  |  | 2,500,001 or more [Col] | | | 6 |  |  |
|  |  | 0 - 10,000 [Ne] | | | 7 |  |  |
|  |  | 10,001 - 20,000 [Ne] | | | 8 |  |  |
|  |  | 20,001 - 30,000 [Ne] | | | 9 |  |  |
|  |  | 30,001 - 40,000 [Ne] | | | 10 |  |  |
|  |  | 40,001 - 60,000 [Ne] | | | 11 |  |  |
|  |  | 60,001 or more [Ne] | | | 12 |  |  |
|  |  | 0 - 5,000 [SA] | | | 13 |  |  |
|  |  | 5,001 - 10,000 [SA] | | | 14 |  |  |
|  |  | 10,001 - 15,000 [SA] | | | 15 |  |  |
|  |  | 15,001 - 20,000 [SA] | | | 16 |  |  |
|  |  | 20,001 - 25,000 [SA] | | | 17 |  |  |
|  |  | 25,001 or more [SA] | | | 18 |  |  |
|  |  | Prefer not to say | | | 99 |  |  |
| C21 | *[Baseline only]*  In the last five years, has the household borrowed from: [select multiple] | Did not borrow money [Go to C33] | | | 0 | cg_borrowed | |
|  |  | Bank | | | 1 |  |  |
|  |  | Micro finance or cooperatives | | | 2 |  |  |
|  |  | Money lender | | | 3 |  |  |
|  |  | Employer | | | 4 |  |  |
|  |  | Friends | | | 5 |  |  |
|  |  | Relatives | | | 6 |  |  |
|  |  | Commercial houses or pawn shops [Col] | | | 7 |  |  |
|  |  | Compensation organisations, cooperatives or employees [Col] | | | 8 |  |  |
|  |  | Other [Answer C23] | | | 77 |  |  |
|  |  | Don’t know | | | 88 |  |  |
|  |  | Prefer not to respond | | | 99 |  |  |
| C22 | *[Follow-up only]*  In the last 6 months, has the household borrowed from: [select multiple] | Did not borrow money [Go to C33] | | | 0 | cg_borrowed_6m | |
|  |  | Bank | | | 1 |  |  |
|  |  | Micro finance or cooperatives | | | 2 |  |  |
|  |  | Money lender | | | 3 |  |  |
|  |  | Employer | | | 4 |  |  |
|  |  | Friends | | | 5 |  |  |
|  |  | Relatives | | | 6 |  |  |
|  |  | Commercial houses or pawn shops [Col] | | | 7 |  |  |
|  |  | Compensation organisations, cooperatives or employees [Col] | | | 8 |  |  |
|  |  | Other [Answer C23] | | | 77 |  |  |
|  |  | Don’t know | | | 88 |  |  |
|  |  | Prefer not to respond | | | 99 |  |  |
| C23 | Please specify who else you borrowed money from | 🖎 | | |  | cg_borrowedo | |
| C24 | *[Baseline only] [Nepal and South Africa]*  Considering all debts you currently have, how much in total does your household currently owe to others? | 🖎 | | |  | total_debts | |
|  |  | I don’t know | | | 88 |  |  |
|  |  | Prefer not to answer | | | 99 |  |  |
| C25 | *[Baseline only] [Colombia only]* Considering all debts you currently have, how much in total does your household currently owe to others? | Less than 300,000 | | | 1 | total_debts_col | |
|  |  | 300,000 - 700,000 | | | 2 |  |  |
|  |  | 700,001 - 1,100,000 | | | 3 |  |  |
|  |  | 1,100,001 - 1,500,000 | | | 4 |  |  |
|  |  | 1,500,001 - 2,000,000 | | | 5 |  |  |
|  |  | 2,500,001 - 3,000,000 | | | 6 |  |  |
|  |  | 3,000,001 - 4,000,000 | | | 7 |  |  |
|  |  | More than 4,000,000 | | | 8 |  |  |
|  |  | Don’t know | | | 88 |  |  |
|  |  | Prefer not to respond | | | 99 |  |  |
| C26 | *[Baseline only]* Please think about the largest loan taken over the last 5 years, how much did you borrow in this case? | 🖎 | | |  | largest_loan | |
|  |  | I don’t know | | | 88 |  |  |
|  |  | Prefer not to answer | | | 99 |  |  |
| C27 | *[Baseline only]* What was the interest rate on this loan? (i.e. percentage % of interest paid on the loan) | 🖎 [Answer C28] | | |  | interest_loan | |
|  |  | I don’t know | | | 88 |  |  |
|  |  | Prefer not to answer | | | 99 |  |  |
| C28 | *[Baseline only]* Is this a monthly or yearly interest rate? | Monthly | | | 1 | interest_loan_time | |
|  |  | Yearly | | | 2 |  |  |
|  |  | Other [Answer C29] | | | 77 |  |  |
|  |  | Don’t know | | | 88 |  |  |
| C29 | *[Baseline only]* Please specify what other type of interest rate | 🖎 | | |  | interest_loan_timeo | |
| C30 | *[Baseline only]*  Has the loan been paid off? | No [Answer C32] | | | 0 | paid_off | |
|  |  | Yes | | | 1 |  |  |
|  |  | Prefer not to respond | | | 99 |  |  |
| C31 | *[Follow-up only]*  When you were last assessed on [date], you indicated that the largest loan taken over the previous 5 years was of [largest_loan], and that you had not yet paid off the loan. Has the loan been paid off now? | No [Answer C32] | | | 0 | paid_off_6m | |
|  |  | Yes | | | 1 |  |  |
|  |  | Prefer not to respond | | | 99 |  |  |
| C32 | What proportion of the loan has been paid off? | Nothing (0%) | | | 1 | prop_paid | |
|  |  | A little (1-25%) | | | 2 |  |  |
|  |  | Less than half (25-49%) | | | 3 |  |  |
|  |  | Half (50%) | | | 4 |  |  |
|  |  | More than half (51-74%) | | | 5 |  |  |
|  |  | Almost all (75-99%) | | | 6 |  |  |
|  |  | Prefer not to respond | | | 99 |  |  |
| C33 | Over the last month, did you buy any of the following items for your household? | Entertainment or recreational activities (e.g., cinema, picnics | | | 1 | cg_eco_consumption | |
|  |  | Parties/celebrations | | | 2 |  |  |
|  |  | Food/groceries | | | 3 |  |  |
|  |  | Clothing for adults | | | 4 |  |  |
|  |  | Clothing for children | | | 5 |  |  |
|  |  | Shoes for adults | | | 6 |  |  |
|  |  | Shoes for children | | | 7 |  |  |
|  |  | Dining out | | | 8 |  |  |
|  |  | Books | | | 9 |  |  |
|  |  | School supplies | | | 10 |  |  |
|  |  | Beauty salon / hair salon | | | 11 |  |  |
|  |  | Jewelry / piercing | | | 12 |  |  |
|  |  | Digital devices (cell phone, tablet) | | | 13 |  |  |
|  |  | Other [Answer C34] | | | 77 |  |  |
|  |  | Don't know | | | 88 |  |  |
|  |  | Prefer not to respond | | | 99 |  |  |
| C34 | Please specify what other items you bought | 🖎 | | | | cg_eco_consumptiono | |
| C35 | *[Nepal and South Africa]* What is your monthly total household expenditure (in local currency)? | 🖎 | | |  | total_expenditure | |
|  |  | I don’t know | | | 88 |  |  |
|  |  | Prefer not to answer | | | 99 |  |  |
| C36 | *[Colombia]* What is your monthly total household expenditure (in local currency)? | Less than 700,000 | | | 1 | total_expenditure_col | |
|  |  | 700,001 - 1,100,000 | | | 2 |  |  |
|  |  | 1,100,001 – 1,500,000 | | | 3 |  |  |
|  |  | 1,500,001 – 2,000,000 | | | 4 |  |  |
|  |  | 2,000,001 – 2,500,000 | | | 5 |  |  |
|  |  | More than 2,500,001 | | | 6 |  |  |
|  |  | Don’t know | | | 88 |  |  |
|  |  | Prefer not to respond | | | 99 |  |  |
| C37 | How much does your household spend on food a month (local currency)? | 🖎 | | |  | food_expenditure | |
|  |  | I don’t know | | | 88 |  |  |
|  |  | Prefer not to answer | | | 99 |  |  |
| C38 | Do you have enough money to buy the food you need for your household each month | None | | | 0 | foodmoney | |
|  |  | A little bit | | | 1 |  |  |
|  |  | Some | | | 2 |  |  |
|  |  | A lot | | | 3 |  |  |
|  |  | Prefer not to respond | | | 99 |  |  |
| C39 | How much was spent in your household for health-related expenses in the past month? (e.g. medical bills, medicines etc.) (in local currency) | 🖎 | | |  | medical_expenditure | |
|  |  | Don’t know | | | 88 |  |  |
|  |  | Prefer not to respond | | | 99 |  |  |
| C40 | How much is the monthly school expenses for all children in the house (fees, transport, lunches etc.)? | 🖎 | | |  | school_fees | |
|  |  | Don’t know | | | 88 |  |  |
|  |  | Prefer not to respond | | | 99 |  |  |
| C41 | Suppose you are given a cash transfer of 4000NPR/R2000/200,000COP, would you share it with others? (For instance, family members, relatives, friends, etc.) | Definitely yes | | | 1 | cg_eco_share2 | |
|  |  | Probably yes | | | 2 |  |  |
|  |  | Probably not | | | 3 |  |  |
|  |  | Definitely not | | | 4 |  |  |
|  |  | Don’t know | | | 88 |  |  |
|  |  | Prefer not to respond | | | 99 |  |  |
| C42 | If someone gives you 2 options – either receive 200NPR/R100/10000COP guaranteed today or 240NPR/R120/12000COP in one month’s time – which option, would you choose? | I would take the 200NPR/R100/10000COP guaranteed today | | | 1 | risk_preference_1 | |
|  |  | I would take the 240NPR/R120/12000COP guaranteed in one month’s time | | | 2 |  |  |
|  |  | Don’t know | | | 88 |  |  |
|  |  | Prefer not to respond | | | 99 |  |  |
| C43 | If someone gives you 2 options – either receive 200NPR/R100/10000COP guaranteed in six months or 140NPR/R120/12000COP in seven month’s time – which option, would you choose? | I would take the 200NPR/R100/10000COP guaranteed today | | | 1 | risk_preference_2 | |
|  |  | I would take the 240NPR/R120/12000COP guaranteed in seven month’s time | | | 2 |  |  |
|  |  | Don’t know | | | 88 |  |  |
|  |  | Prefer not to respond | | | 99 |  |  |
| C44 | Suppose you receive 20,000 pesos/NRs 400/ R200. You can invest any portion of this money in a risky option , which has a 50-50 (%) chance that the investment will fail or succeed. The rest of the money will be yours to keep. If the investment fails, you lose the amount you invested. If the investment succeeds, you receive 3 times the amount invested. How much would you like to invest?  Start with an example: If you choose to invest all of the NRs 400, then if the coin comes up heads, you win NRs 1200, and if the coin comes up tails, you win nothing and end up with 0 | 🖎 | | |  | cg_eco_finance | |
|  |  | Don’t know | | | 88 |  |  |
|  |  | Prefer not to respond | | | 99 |  |  |
| C45 | Have you applied for any scholarships or financial help for school in the last 6 months for your index child’s current schooling? (e.g. providing school uniforms, or transportation to school) | No | | | 0 | cg_scholarships | |
|  |  | Yes | | | 1 |  |  |
|  |  | Don’t know | | | 88 |  |  |
|  |  | Prefer not to respond | | | 99 |  |  |
| C46 | Do you expect that the index child will register in school next year? | No | | | 0 | cg_enrol | |
|  |  | Yes | | | 1 |  |  |
|  |  | Prefer not to respond | | | 99 |  |  |
| C47 | What level of schooling would you like your index child to achieve? | None | | | 0 | cg_schooling_hope | |
|  |  | Elementary school (grade 5) [Col] | | | 1 |  |  |
|  |  | Secondary school (grade 9) [Col] | | | 2 |  |  |
|  |  | High school (grade 11) [Col] | | | 3 |  |  |
|  |  | Technician or technologist (e.g. studying at SENA) [Col] | | | 4 |  |  |
|  |  | University degree [Col] | | | 5 |  |  |
|  |  | Specialization or master's degree [Col] | | | 6 |  |  |
|  |  | 10+2 [Ne] | | | 7 |  |  |
|  |  | Bachelors [Ne] | | | 8 |  |  |
|  |  | Masters or above [Ne] | | | 9 |  |  |
|  |  | Primary (till grade 7 or equivalent) [SA] | | | 10 |  |  |
|  |  | Secondary (e.g. high school/technical or vocational school) [SA] | | | 11 |  |  |
|  |  | University/tertiary (e.g. University/Technikon, College) [SA] | | | 12 |  |  |
|  |  | Other [Answer C48] | | | 77 |  |  |
|  |  | Don't know | | | 88 |  |  |
|  |  | Prefer not to respond | | | 99 |  |  |
| C48 | What other level? | 🖎 | | |  | cg_schooling_hopeo | |
| C49 | What level of schooling do you think the index child will actually achieve? | None | | | 0 | cg_schooling_actual | |
|  |  | Elementary school (grade 5) [Col] | | | 1 |  |  |
|  |  | Secondary school (grade 9) [Col] | | | 2 |  |  |
|  |  | High school (grade 11) [Col] | | | 3 |  |  |
|  |  | Technician or technologist (e.g. studying at SENA) [Col] | | | 4 |  |  |
|  |  | University degree [Col] | | | 5 |  |  |
|  |  | Specialization or master's degree [Col] | | | 6 |  |  |
|  |  | 10+2 [Ne] | | | 7 |  |  |
|  |  | Bachelors [Ne] | | | 8 |  |  |
|  |  | Masters or above [Ne] | | | 9 |  |  |
|  |  | Primary (till grade 7 or equivalent) [SA] | | | 10 |  |  |
|  |  | Secondary (e.g. high school/technical or vocational school) [SA] | | | 11 |  |  |
|  |  | University/tertiary (e.g. University/Technikon, College) [SA] | | | 12 |  |  |
|  |  | Other [Answer C50] | | | 77 |  |  |
|  |  | Don't know | | | 88 |  |  |
|  |  | Prefer not to respond | | | 99 |  |  |
| C50 | What other level? | 🖎 | | |  | cg_schooling_actualo | |
| C51 | What type of job or occupation would you like the index child to have when the index child finishes their maximum level of education? | 🖎 | | |  | cg_eco_jobwant | |
| C52 | What other job? | 🖎 | | |  | cg_eco_jobwanto | |
| C53 | What level of monthly income (local currency) do you think the index child would earn when they finish their maximum level of education? | 0 - 700,000 [Col] | | | 1 | cg_eco_earn_maxedu | |
|  |  | 700,001 - 1,100,000 [Col] | | | 2 |  |  |
|  |  | 1,100,001 - 1,500,000 [Col] | | | 3 |  |  |
|  |  | 1,500,001 - 2,000,000 [Col] | | | 4 |  |  |
|  |  | 2,000,001 - 2,500,000 [Col] | | | 5 |  |  |
|  |  | 2,500,000 or more [Col] | | | 6 |  |  |
|  |  | 0 - 10,000 [Ne] | | | 1 |  |  |
|  |  | 10,001 - 20,000 [Ne] | | | 2 |  |  |
|  |  | 20,001 - 30,000 [Ne] | | | 3 |  |  |
|  |  | 30,001 - 40,000 [Ne] | | | 4 |  |  |
|  |  | 40,001 - 60,000 [Ne] | | | 5 |  |  |
|  |  | 60,001 or more [Ne] | | | 6 |  |  |
|  |  | 0 - 5,000 [SA] | | | 1 |  |  |
|  |  | 5,001 - 10,000 [SA] | | | 2 |  |  |
|  |  | 10,001 - 15,000 [SA] | | | 3 |  |  |
|  |  | 15,001 - 20,000 [SA] | | | 4 |  |  |
|  |  | 20,001 - 25,000 [SA] | | | 5 |  |  |
|  |  | 25,001 or more [SA] | | | 6 |  |  |
|  |  | Don't know | | | 88 |  |  |
|  |  | Prefer not to respond | | | 99 |  |  |
| C54 | What level of monthly income (local currency) do you think the index child would earn at age 25 if the index child left the school after secondary school and did not pursue any other form of additional schooling or vocational/professional training? | 0 - 700,000 [Col] | | | 1 | cg_eco_earn_secondary | |
|  |  | 700,001 - 1,100,000 [Col] | | | 2 |  |  |
|  |  | 1,100,001 - 1,500,000 [Col] | | | 3 |  |  |
|  |  | 1,500,001 - 2,000,000 [Col] | | | 4 |  |  |
|  |  | 2,000,001 - 2,500,000 [Col] | | | 5 |  |  |
|  |  | 2,500,000 or more [Col] | | | 6 |  |  |
|  |  | 0 - 10,000 [Ne] | | | 1 |  |  |
|  |  | 10,001 - 20,000 [Ne] | | | 2 |  |  |
|  |  | 20,001 - 30,000 [Ne] | | | 3 |  |  |
|  |  | 30,001 - 40,000 [Ne] | | | 4 |  |  |
|  |  | 40,001 - 60,000 [Ne] | | | 5 |  |  |
|  |  | 60,001 or more [Ne] | | | 6 |  |  |
|  |  | 0 - 5,000 [SA] | | | 1 |  |  |
|  |  | 5,001 - 10,000 [SA] | | | 2 |  |  |
|  |  | 10,001 - 15,000 [SA] | | | 3 |  |  |
|  |  | 15,001 - 20,000 [SA] | | | 4 |  |  |
|  |  | 20,001 - 25,000 [SA] | | | 5 |  |  |
|  |  | 25,001 or more [SA] | | | 6 |  |  |
|  |  | Don't know | | | 88 |  |  |
|  |  | Prefer not to respond | | | 99 |  |  |
| C55 | In the future, would you like the index child to migrate/relocate somewhere? | No | | | 0 | eco_migrate | |
|  |  | Yes, within the district | | | 1 |  |  |
|  |  | Yes, outside the district | | | 2 |  |  |
|  |  | Yes, outside the country | | | 3 |  |  |
|  |  | Don’t know | | | 88 |  |  |
|  |  | Prefer not to answer | | | 99 |  |  |
| C56 | In the future, would you like the index child to get married by the age of 25? | No | | | 0 | cg_eco_marry25 | |
|  |  | Yes | | | 1 |  |  |
|  |  | Don’t know | | | 88 |  |  |
|  |  | Prefer not to respond | | | 99 |  |  |
| C57 | In the future, would you like the index child to have children by the age of 25? [skip if Nepal and do not answer ‘Yes’ to C56] | No | | | 0 | cg_eco_children25 | |
|  |  | Yes | | | 1 |  |  |
|  |  | Don’t know | | | 88 |  |  |
|  |  | Prefer not to respond | | | 99 |  |  |
| C58 | Which of the following do you think is true for adolescents who complete secondary schooling: | They are more likely to end up working in a paid job than those who do not complete it | | 1 | | | cg_eco_beliefs_1 |
|  |  | They are equally likely to end up working in a paid job than those who do not complete it | | 2 | | |  |
|  |  | They are less likely to end up working in a paid job than those who do not complete it | | 3 | | |  |
|  |  | Prefer not to respond | | 99 | | |  |
| C59 | Which of the following do you think is true for adolescents who complete secondary schooling: | They are likely to earn more in their lifetime than those who do not complete it | | 1 | | | cg_eco_beliefs_2 |
|  |  | They are likely to earn similarly in their lifetime than those who do not complete it | | 2 | | |  |
|  |  | They are likely to earn less in their lifetime than those who do not complete it | | 3 | | |  |
|  |  | Prefer not to answer | | 99 | | |  |
| C60 | What do you think is the average monthly salary of someone who completed secondary education in your area? | 0 - 700,000 [Col] | | 1 | | | cg_ eco_salary_secondary |
|  |  | 700,001 - 1,100,000 [Col] | | 2 | | |  |
|  |  | 1,100,001 - 1,500,000 [Col] | | 3 | | |  |
|  |  | 1,500,001 - 2,000,000 [Col] | | 4 | | |  |
|  |  | 2,000,001 - 2,500,000 [Col] | | 5 | | |  |
|  |  | 2,500,000 or more [Col] | | 6 | | |  |
|  |  | 0 - 10,000 [Ne] | | 1 | | |  |
|  |  | 10,001 - 20,000 [Ne] | | 2 | | |  |
|  |  | 20,001 - 30,000 [Ne] | | 3 | | |  |
|  |  | 30,001 - 40,000 [Ne] | | 4 | | |  |
|  |  | 40,001 - 60,000 [Ne] | | 5 | | |  |
|  |  | 60,001 or more [Ne] | | 6 | | |  |
|  |  | 0 - 5,000 [SA] | | 1 | | |  |
|  |  | 5,001 - 10,000 [SA] | | 2 | | |  |
|  |  | 10,001 - 15,000 [SA] | | 3 | | |  |
|  |  | 15,001 - 20,000 [SA] | | 4 | | |  |
|  |  | 20,001 - 25,000 [SA] | | 5 | | |  |
|  |  | 25,001 or more [SA] | | 6 | | |  |
|  |  | Don't know | | 88 | | |  |
|  |  | Prefer not to respond | | 99 | | |  |
| C61 | What do you think is the average monthly salary of someone who completed university in your area? | 0 - 700,000 [Col] | | 1 | | | cg_ eco_salary_tertiary |
|  |  | 700,001 - 1,100,000 [Col] | | 2 | | |  |
|  |  | 1,100,001 - 1,500,000 [Col] | | 3 | | |  |
|  |  | 1,500,001 - 2,000,000 [Col] | | 4 | | |  |
|  |  | 2,000,001 - 2,500,000 [Col] | | 5 | | |  |
|  |  | 2,500,000 or more [Col] | | 6 | | |  |
|  |  | 0 - 10,000 [Ne] | | 1 | | |  |
|  |  | 10,001 - 20,000 [Ne] | | 2 | | |  |
|  |  | 20,001 - 30,000 [Ne] | | 3 | | |  |
|  |  | 30,001 - 40,000 [Ne] | | 4 | | |  |
|  |  | 40,001 - 60,000 [Ne] | | 5 | | |  |
|  |  | 60,001 or more [Ne] | | 6 | | |  |
|  |  | 0 - 5,000 [SA] | | 1 | | |  |
|  |  | 5,001 - 10,000 [SA] | | 2 | | |  |
|  |  | 10,001 - 15,000 [SA] | | 3 | | |  |
|  |  | 15,001 - 20,000 [SA] | | 4 | | |  |
|  |  | 20,001 - 25,000 [SA] | | 5 | | |  |
|  |  | 25,001 or more [SA] | | 6 | | |  |
|  |  | Don't know | | 88 | | |  |
|  |  | Prefer not to respond | | 99 | | |  |
| C62 | What do you think is the average monthly salary of a someone without secondary education in your area? | 0 - 700,000 [Col] | | 1 | | | cg_ eco_salary_noschool |
|  |  | 700,001 - 1,100,000 [Col] | | 2 | | |  |
|  |  | 1,100,001 - 1,500,000 [Col] | | 3 | | |  |
|  |  | 1,500,001 - 2,000,000 [Col] | | 4 | | |  |
|  |  | 2,000,001 - 2,500,000 [Col] | | 5 | | |  |
|  |  | 2,500,000 or more [Col] | | 6 | | |  |
|  |  | 0 - 10,000 [Ne] | | 1 | | |  |
|  |  | 10,001 - 20,000 [Ne] | | 2 | | |  |
|  |  | 20,001 - 30,000 [Ne] | | 3 | | |  |
|  |  | 30,001 - 40,000 [Ne] | | 4 | | |  |
|  |  | 40,001 - 60,000 [Ne] | | 5 | | |  |
|  |  | 60,001 or more [Ne] | | 6 | | |  |
|  |  | 0 - 5,000 [SA] | | 1 | | |  |
|  |  | 5,001 - 10,000 [SA] | | 2 | | |  |
|  |  | 10,001 - 15,000 [SA] | | 3 | | |  |
|  |  | 15,001 - 20,000 [SA] | | 4 | | |  |
|  |  | 20,001 - 25,000 [SA] | | 5 | | |  |
|  |  | 25,001 or more [SA] | | 6 | | |  |
|  |  | Don't know | | 88 | | |  |
|  |  | Prefer not to respond | | 99 | | |  |
| C63 | What is the level of monthly income that you would like to achieve in your life? | 0 - 700,000 [Col] | | 1 | | | aspiration_income |
|  |  | 700,001 - 1,100,000 [Col] | | 2 | | |  |
|  |  | 1,100,001 - 1,500,000 [Col] | | 3 | | |  |
|  |  | 1,500,001 - 2,000,000 [Col] | | 4 | | |  |
|  |  | 2,000,001 - 2,500,000 [Col] | | 5 | | |  |
|  |  | 2,500,000 or more [Col] | | 6 | | |  |
|  |  | 0 - 10,000 [Ne] | | 1 | | |  |
|  |  | 10,001 - 20,000 [Ne] | | 2 | | |  |
|  |  | 20,001 - 30,000 [Ne] | | 3 | | |  |
|  |  | 30,001 - 40,000 [Ne] | | 4 | | |  |
|  |  | 40,001 - 60,000 [Ne] | | 5 | | |  |
|  |  | 60,001 or more [Ne] | | 6 | | |  |
|  |  | 0 - 5,000 [SA] | | 1 | | |  |
|  |  | 5,001 - 10,000 [SA] | | 2 | | |  |
|  |  | 10,001 - 15,000 [SA] | | 3 | | |  |
|  |  | 15,001 - 20,000 [SA] | | 4 | | |  |
|  |  | 20,001 - 25,000 [SA] | | 5 | | |  |
|  |  | 25,001 or more [SA] | | 6 | | |  |
|  |  | I am happy with what I have now | | 66 | | |  |
|  |  | Don't know | | 88 | | |  |
|  |  | Prefer not to respond | | 99 | | |  |
| C64 | What is the level of capital/assets you would like to achieve in your life? (in local currency) | 🖎 (in local currency) | |  | | | aspiration_asset |
| C65 | Which health insurance are you affiliated with? | No health insurance | | 0 | | | health_insurance |
|  |  | Pay full amount (private) | | 1 | | |  |
|  |  | Subsidised (pay a portion of it) | | 2 | | |  |
|  |  | Pay full amount by government or others as grant [Ne] | | 3 | | |  |
|  |  | Special regime (e.g. veteran plan) [SA] | | 4 | | |  |
|  |  | Other [Answer C66] | | 77 | | |  |
|  |  | Don't know | | 88 | | |  |
|  |  | Prefer not to say | | 99 | | |  |
| C66 | Specify which other health insurance | 🖎 | |  | | | health_insuranco |
| The following items pertain to indicators on the Multidimensional Poverty Index (Colombia only) *[Baseline and 18-month post baseline only]* | | | | | | | |
| C67 | In the past 30 days, has anyone in the household suffered an illness, an accident, dental problem or any other health issue that didn’t require hospitalisation (30 days)? | No | | 0 | | | mpi_col_illness |
|  |  | Yes [Answer C68] | | 1 | | |  |
|  |  | Prefer not to respond | | 99 | | |  |
| C68 | Was the member attended to by a doctor, specialist, dentist, therapist or health institution? *[enter no if at least one didn’t receive services]* | No | | 0 | | | mpi_col_service |
|  |  | Yes | | 1 | | |  |
|  |  | Prefer not to respond | | 99 | | |  |
| C69 | What is the household’s main source of drinking water? | Piped into dwelling | | 1 | | | mpi_col_water |
|  |  | Public tap | | 3 | | |  |
|  |  | Protected dug well | | 5 | | |  |
|  |  | Unprotected dug well | | 6 | | |  |
|  |  | Rain water collection | | 9 | | |  |
|  |  | Bottled water | | 10 | | |  |
|  |  | Small scale vendor | | 11 | | |  |
|  |  | Tanker-truck | | 12 | | |  |
|  |  | Surface water | | 13 | | |  |
|  |  | Other [Answer C70] | | 77 | | |  |
|  |  | Don’t know | | 88 | | |  |
|  |  | Prefer not to respond | | 99 | | |  |
| C70 | What other main source of drinking water? | 🖎 | |  | | | mpi_col_watero |
| C71 | What type of toilet facility is available for this household? | Toilet connected to sewer | | 1 | | | mpi_col_toilet |
|  |  | Toilet connected to septic tank | | 2 | | |  |
|  |  | Offline toilet | | 3 | | |  |
|  |  | Latrine | | 4 | | |  |
|  |  | Toilet with direct discharge to water sources (low tide) | | 5 | | |  |
|  |  | No facilities/bush/field | | 0 | | |  |
|  |  | Other [Answer C72] | | 77 | | |  |
|  |  | Don’t know | | 88 | | |  |
|  |  | Prefer not to respond | | 99 | | |  |
| C72 | What other type of toilet facility? | 🖎 | |  | | | mpi_col_toileto |
| C73 | What is the main material of the floor of the household? | Wall-to-wall rug or mat | | 1 | | | mpi_col_floor |
|  |  | Polished and lacquered wood, parquet | | 2 | | |  |
|  |  | Marble | | 3 | | |  |
|  |  | Tile, vinyl, tablet, brick, laminate | | 4 | | |  |
|  |  | Coarse wood, board, plank, other plant | | 5 | | |  |
|  |  | Cement, gravel | | 6 | | |  |
|  |  | Sand earth | | 7 | | |  |
|  |  | Other [Answer C74] | | 77 | | |  |
|  |  | Don’t know | | 88 | | |  |
|  |  | Prefer not to respond | | 99 | | |  |
| C74 | What other floor material? | 🖎 | |  | | | mpi_col_flooro |
| C75 | What is the main material of the walls of the household? | Block, brick, stone, polished wood | | 1 | | | mpi_col_walls |
|  |  | Stepped wall, adobe | | 2 | | |  |
|  |  | Bahareque revoked | | 3 | | |  |
|  |  | Bahareque without revoking | | 4 | | |  |
|  |  | rough wood, board, plank | | 5 | | |  |
|  |  | prefabricated material | | 6 | | |  |
|  |  | Guadua, cane, mat, other vegetable | | 7 | | |  |
|  |  | Zinc, cloth, carbon, cans, waste, plastic | | 8 | | |  |
|  |  | Without walls | | 0 | | |  |
|  |  | Other [Answer C76] | | 77 | | |  |
|  |  | Don’t know | | 88 | | |  |
|  |  | Prefer not to respond | | 99 | | |  |
| C76 | What other wall material? | 🖎 | |  | | | mpi_col_wallso |
| C77 | How many rooms are there in the household (other than kitchen, bathroom, toilet and garage)? |  | |  | | | mpi_col_rooms |
| The following items pertain to indicators on the Multidimensional Poverty Index (South Africa only) *[Baseline and 18-month post baseline only]* | | | | | | | |
| C78 | What is the main source of energy/fuel for this household for lighting? | None | | 0 | | | mpi_sa_lighting |
|  |  | Electricity | | 1 | | |  |
|  |  | Petrol, gas, kerosene, alcohol | | 10 | | |  |
|  |  | Natural gas connected to a public grid | | 11 | | |  |
|  |  | Propane gas in cyinder or pipette | | 12 | | |  |
|  |  | Firewood, wood or charcoal | | 13 | | |  |
|  |  | Mineral carbon | | 14 | | |  |
|  |  | Waste material | | 15 | | |  |
|  |  | Other [Answer C79] | | 77 | | |  |
|  |  | Don’t know | | 88 | | |  |
|  |  | Prefer not to respond | | 99 | | |  |
| C79 | What other source of energy for lighting? | 🖎 | |  | | | mpi_sa_lightingo |
| C80 | What is the main source of energy/fuel for this household for heating? | None | | 0 | | | mpi_sa_heating |
|  |  | Electricity from mains | | 1 | | |  |
|  |  | Electricity from generator | | 2 | | |  |
|  |  | Gas | | 3 | | |  |
|  |  | Paraffin | | 4 | | |  |
|  |  | Wood | | 5 | | |  |
|  |  | Coal | | 6 | | |  |
|  |  | Candles, | | 7 | | |  |
|  |  | Animal dung | | 8 | | |  |
|  |  | Solar energy | | 9 | | |  |
|  |  | Other [Answer C81] | | 77 | | |  |
|  |  | Don’t know | | 88 | | |  |
|  |  | Prefer not to respond | | 99 | | |  |
| C81 | What other source of energy for heating? | 🖎 | |  | | | mpi_sa_heatingo |
| C82 | What is the main source of energy/fuel for this household for cooking? | None | | 0 | | | mpi_sa_cooking |
|  |  | Electricity from mains, | | 1 | | |  |
|  |  | Electricity from generator | | 2 | | |  |
|  |  | Gas | | 3 | | |  |
|  |  | Paraffin, | | 4 | | |  |
|  |  | Wood | | 5 | | |  |
|  |  | Coal | | 6 | | |  |
|  |  | Candles, | | 7 | | |  |
|  |  | Animal dung | | 8 | | |  |
|  |  | Solar energy | | 9 | | |  |
|  |  | Other [Answer C83] | | 77 | | |  |
|  |  | Don’t know | | 88 | | |  |
|  |  | Prefer not to respond | | 99 | | |  |
| C83 | What other source of energy for cooking? | 🖎 | |  | | | mpi_sa_cookingo |
| C84 | What is the household’s main source of water? | Piped into dwelling | | 1 | | | mpi_sa_water |
|  |  | Piped into yard/plot | | 2 | | |  |
|  |  | Public tap | | 3 | | |  |
|  |  | Borehole (e.g. deep hole made in ground to get water) | | 4 | | |  |
|  |  | Protected dug well | | 5 | | |  |
|  |  | Unprotected dug well | | 6 | | |  |
|  |  | Protected spring | | 7 | | |  |
|  |  | Unprotected Spring | | 8 | | |  |
|  |  | Rain water collection | | 9 | | |  |
|  |  | Bottled water (e.g. bought from shop, spaza shop) | | 10 | | |  |
|  |  | Small scale vendor (e.g. deliver water in bottles, water shop) | | 11 | | |  |
|  |  | Tanker-truck (e.g. big trucks that bring water to village/community) | | 12 | | |  |
|  |  | Surface water (e.g. rivers, lakes, dams) | | 13 | | |  |
|  |  | Other [Answer C85] | | 77 | | |  |
|  |  | Don’t know | | 88 | | |  |
|  |  | Prefer not to respond | | 99 | | |  |
| C85 | What other source of water? | 🖎 | |  | | | mpi_sa_watero |
| C86 | What type of toilet facility is available for this household? | Flush to piped sewer system | | 1 | | | mpi_sa_toilet |
|  |  | Flush to septic tank | | 2 | | |  |
|  |  | Flush to pit latrine | | 3 | | |  |
|  |  | Flush to other location | | 4 | | |  |
|  |  | Flush to unknown place | | 5 | | |  |
|  |  | Ventilated improved pit latrine | | 6 | | |  |
|  |  | Pit latrine with slab | | 7 | | |  |
|  |  | Pit latrine without slab/open pit (e.g. hole in ground without covering slab) | | 8 | | |  |
|  |  | Composting toilet | | 9 | | |  |
|  |  | Bucket latrine | | 10 | | |  |
|  |  | Hanging toilet/latrine | | 11 | | |  |
|  |  | Community toilets (e.g. portable/mobile toilets, 'porta-potty', chemical toilet) | | 12 | | |  |
|  |  | No facilities/bush/field | | 0 | | |  |
|  |  | Other [Answer C87] | | 77 | | |  |
|  |  | Don’t know | | 88 | | |  |
|  |  | Prefer not to respond | | 99 | | |  |
| C87 | What other type of toilet facility? | 🖎 | |  | | | mpi_sa_toileto |
| C88 | What type of household do you live in? | Dwelling/house or brick structure on separate stand, | | 1 | | | mpi_sa_hh |
|  |  | Traditional dwelling/hut/structure made of traditional materials, | | 2 | | |  |
|  |  | Flat/apartment, | | 3 | | |  |
|  |  | Town/cluster/semi-detached house, | | 4 | | |  |
|  |  | Dwelling/house/flat/room in backyard, | | 5 | | |  |
|  |  | Informal dwelling/shack in backyard, | | 6 | | |  |
|  |  | Informal dwelling/shack not in backyard, | | 7 | | |  |
|  |  | Room/flatlet, | | 8 | | |  |
|  |  | Caravan/tent, | | 9 | | |  |
|  |  | Other [Answer C89] | | 77 | | |  |
|  |  | Don’t know | | 88 | | |  |
|  |  | Prefer not to respond | | 99 | | |  |
| C89 | Where other type of household? | 🖎 | |  | | | mpi_sa_hho |
| C90 | Do you own any of the following? [Select all that apply] | | | | | | mpi_sa |
|  | radio | No | | 0 | | | _radio |
|  |  | Yes | | 1 | | |  |
|  | television | No | | 0 | | | _tv |
|  |  | Yes | | 1 | | |  |
|  | telephone | No | | 0 | | | _phone |
|  |  | Yes | | 1 | | |  |
|  | refrigerator | No | | 0 | | | _fridge |
|  |  | Yes | | 1 | | |  |
|  | car | No | | 0 | | | _car |
|  |  | Yes | | 1 | | |  |
| The following items pertain to indicators on the Multidimensional Poverty Index (Nepal only) *[Baseline and 18-month post baseline only]* | | | | | | | |
| C91 | What is the main source of energy/fuel for this household for cooking? | None | | 0 | | | mpi_ne_cooking |
|  |  | Electricity | | 1 | | |  |
|  |  | LP gas | | 3 | | |  |
|  |  | Biogas | | 10 | | |  |
|  |  | Kerosene | | 4 | | |  |
|  |  | Wood | | 5 | | |  |
|  |  | Animal dung | | 8 | | |  |
|  |  | Other [Answer C92] | | 77 | | |  |
|  |  | Don’t know | | 88 | | |  |
|  |  | Prefer not to respond | | 99 | | |  |
| C92 | What other source of energy for cooking? | 🖎 | |  | | | mpi_ne_cookingo |
| C93 | What type of toilet facility is available for this household? | Flush to piped sewer system | | 1 | | | mpi_ne_toilet |
|  |  | Flush to septic tank | | 2 | | |  |
|  |  | Flush to pit latrine | | 3 | | |  |
|  |  | Public toilet | | 12 | | |  |
|  |  | No facilities/bush/field | | 0 | | |  |
|  |  | Other [Answer C94] | | 77 | | |  |
|  |  | Don’t know | | 88 | | |  |
|  |  | Prefer not to respond | | 99 | | |  |
| C94 | What other type of toilet facility? | 🖎 | |  | | | mpi_ne_toileto |
| C95 | What is the household’s main source of water? | Piped into dwelling | | 1 | | | mpi_ne_water |
|  |  | Piped into yard/plot | | 2 | | |  |
|  |  | Borehole | | 4 | | |  |
|  |  | Protected dug well | | 5 | | |  |
|  |  | Unprotected dug well | | 6 | | |  |
|  |  | Spring water | | 8 | | |  |
|  |  | Bottled water | | 10 | | |  |
|  |  | Tanker-truck | | 12 | | |  |
|  |  | River water | | 13 | | |  |
|  |  | Other [Answer C96] | | 77 | | |  |
|  |  | Don’t know | | 88 | | |  |
|  |  | Prefer not to respond | | 99 | | |  |
| C96 | What other source of water? | 🖎 | |  | | | mpi_ne_watero |
| C97 | Does the household have access to electricity? | No | | 0 | | | mpi_ne_elec |
|  |  | Yes | | 1 | | |  |
|  |  | Don’t know | | 88 | | |  |
|  |  | Prefer not to respond | | 99 | | |  |
| C98 | What is the main material of the floor of the household? | Hard floor (tile, cement, brick, wood); | | 0 | | | mpi_ne_floor |
|  |  | Earth floor | | 1 | | |  |
|  |  | Other [Answer C98] | | 77 | | |  |
|  |  | Don’t know | | 88 | | |  |
|  |  | Prefer not to respond | | 99 | | |  |
| C99 | What other floor material? | 🖎 | |  | | | mpi_ne_flooro |
| C100 | What is the main material of the walls of the household? | Brick/stone and cement | | 1 | | | mpi_ne_walls |
|  |  | Brick/stone and mud | | 2 | | |  |
|  |  | Wood | | 3 | | |  |
|  |  | Corrugated iron/zinc | | 4 | | |  |
|  |  | Fabric boards | | 5 | | |  |
|  |  | Bamboo | | 10 | | |  |
|  |  | Uncooked bricks | | 12 | | |  |
|  |  | Other [Answer C101] | | 77 | | |  |
|  |  | Don’t know | | 88 | | |  |
|  |  | Prefer not to respond | | 99 | | |  |
| C101 | What other wall material? | 🖎 | |  | | | mpi_ne_wallso |
| C102 | What is the main material of the roof of the household? | Tiles | | 1 | | | mpi_ne_roof |
|  |  | Corrugated iron/zinc | | 4 | | |  |
|  |  | Wooden planks | | 6 | | |  |
|  |  | Mud | | 9 | | |  |
|  |  | Straw or grass (Khar) | | 10 | | |  |
|  |  | Reinforced cement concrete (RCC) | | 12 | | |  |
|  |  | Stone or slate | | 13 | | |  |
|  |  | Other [Answer C103] | | 77 | | |  |
|  |  | Don’t know | | 88 | | |  |
|  |  | Prefer not to respond | | 99 | | |  |
| C103 | What other roof material? | 🖎 | |  | | | mpi_ne_roofo |
| C104 | Do you own any of the following? | | | | | | mpi_ne |
|  | radio | No | | 0 | | | _radio |
|  |  | Yes | | 1 | | |  |
|  | television | No | | 0 | | | _tv |
|  |  | Yes | | 1 | | |  |
|  | telephone | No | | 0 | | | _phone |
|  |  | Yes | | 1 | | |  |
|  | Computer | No | | 0 | | | _computer |
|  |  | Yes | | 1 | | |  |
|  | Animal cart | No | | 0 | | | _cart |
|  |  | Yes | | 1 | | |  |
|  | bicycle | No | | 0 | | | _bicycle |
|  |  | Yes | | 1 | | |  |
|  | motorbike | No | | 0 | | | _motorbike |
|  |  | Yes | | 1 | | |  |
|  | refrigerator | No | | 0 | | | _fridge |
|  |  | Yes | | 1 | | |  |
|  | car | No | | 0 | | | _car |
|  |  | Yes | | 1 | | |  |
|  | truck | No | | 0 | | | _truck |
|  |  | Yes | | 1 | | |  |

| D. Client Service Receipt Inventory (Adapted) – Part 1: Caregiver (Beecham & Knapp, 2001)  *[All follow-up assessments]* | | | | |
| --- | --- | --- | --- | --- |
| Instructions:  *“These next questions are about the services that you might have used over the past few months.*  *Every question is important, please try your best to respond each of them. If you do not want to answer a question, please let us know. Remember that all your answers are confidential”* | | | | |
| D1 | Have you attended the emergency department of a hospital in the past 6 months? | No | 0 | cg_emergency |
|  |  | Yes [Answer D2-D4] | 1 |  |
|  |  | Prefer not to respond | 99 |  |
| D2 | How many times did you attend the emergency department of a hospital in the past 6 months? | 🖎 |  | cg_emergency_times |
| D3 | What was the emergency? | 🖎 |  | cg_emergency_why |
| D4 | Did you, your parents or someone else pay for this? | No | 0 | cg_emergency_pay |
|  |  | Yes [Answer D5] | 1 |  |
|  |  | Prefer not to respond | 99 |  |
| D5 | How much did you, your parents or someone else pay? | 🖎 |  | cg_emergency_cost |
| D6 | Have you been admitted to hospital (i.e. staying at least one night) in the past 6 months? | No | 0 | cg_admitted |
|  |  | Yes [Answer D6-D8] | 1 |  |
|  |  | Prefer not to respond | 99 |  |
| D7 | How many days were you in hospital altogether? | 🖎 |  | cg_admitted_days |
| D8 | What were the reasons for your hospital stay? | Infectious disease (e.g. HIV, malaria, TB) | 1 | cg_admitted_why |
|  |  | Maternal / perinatal condition | 2 |  |
|  |  | Acute condition (e.g. flu, cough) | 3 |  |
|  |  | Injury | 4 |  |
|  |  | Sleep problems | 5 |  |
|  |  | Depression or anxiety | 6 |  |
|  |  | Alcohol problems | 7 |  |
|  |  | Other mental health problems | 8 |  |
|  |  | Other chronic disease (e.g. heart, diabetes) | 9 |  |
|  |  | Other reason [Answer D9] | 77 |  |
|  |  | Don’t know | 88 |  |
|  |  | Prefer not to respond | 99 |  |
| D9 | What other reason were you admitted to hospital? | 🖎 |  | cg_admitted_whyo |
| D10 | Did you, your parents or someone else pay for this? | No | 0 | cg_admitted_pay |
|  |  | Yes [Answer D11] | 1 |  |
|  |  | Prefer not to respond | 99 |  |
| D11 | How much did you, your parents or someone else pay? | 🖎 |  | cg_admitted_cost |
| The following section pertains to service use for physical health | | | | |
| D12 | Have you seen someone in the hospital or in the community for your physical health in the past 3 months? | No | 0 | cg_outpatient_physical |
|  |  | Yes [Answer D13] | 1 |  |
|  |  | Prefer not to respond | 99 |  |
| D13 | Total number of visits in the past 3 months | 🖎 |  | cg_phys_times |
| Visit for physical health: Section repeated by number reported under [cg_phys_times] | | | | |
| D14 | Type of practitioner seen | Traditional healer/spiritualist/herbalist | 1 | cg_phys_type |
|  |  | Specialist doctor | 2 |  |
|  |  | Health professional at school | 3 |  |
|  |  | Community health worker | 4 |  |
|  |  | Nurse/midwife | 5 |  |
|  |  | Pharmacist | 6 |  |
|  |  | General medical doctor | 7 |  |
|  |  | Social worker (Female Community Health Volunteer) [Ne] | 8 |  |
|  |  | Occupational therapist | 9 |  |
|  |  | Physiotherapist | 10 |  |
|  |  | Other health worker | 77 |  |
|  |  | Don't know | 88 |  |
|  |  | Prefer not to respond | 99 |  |
| D15 | Other type of practitioner seen | 🖎 |  | cg_phys_typeo |
| D16 | How long did the visit last (including waiting time)? (in minutes) | 🖎 |  | cg_phys_long |
| D17 | How much time did the practitioner spend with you? (in minutes) | 🖎 |  | cg_phys_timespent |
| D18 | How much did you (or someone else) pay for this, including travel expenditure? | 🖎 |  | cg_phys_cost |
| D19 | How long did it take to travel there and back? (in minutes) | 🖎 |  | cg_phys_travel |
| The following section pertains to service use for mental health | | | | |
| D20 | Have you seen someone in the hospital or in the community for your mental health or wellbeing in the past 3 months? | No | 0 | cg_outpatient_mh |
|  |  | Yes [Answer D21] | 1 |  |
|  |  | Prefer not to respond | 99 |  |
| D21 | Total number of visits in the past 3 months | 🖎 |  | cg_mh_times |
| Visit for mental health: Section repeated by number reported under [cg_mh_times] | | | | |
| D22 | Type of practitioner seen | General medical doctor | 1 | cg_mh_type |
|  |  | Psychiatrist | 2 |  |
|  |  | Psychologist | 3 |  |
|  |  | School/student counsellor | 4 |  |
|  |  | Psychosocial counsellor | 5 |  |
|  |  | Psychiatric nurse | 6 |  |
|  |  | Psychiatric clinical officer | 7 |  |
|  |  | Social worker | 8 |  |
|  |  | Religious/spiritual/traditional healer | 9 |  |
|  |  | Specialist doctor (other than psychiatrist) | 10 |  |
|  |  | Occupational therapist | 11 |  |
|  |  | Other practitioner | 77 |  |
|  |  | Don't know | 88 |  |
|  |  | Prefer not to respond | 99 |  |
| D23 | Other type of practitioner seen | 🖎 |  | cg_mh_typeo |
| D24 | How long did the visit last (including waiting time)? (in minutes) | 🖎 |  | cg_mh_long |
| D25 | How much time did the practitioner spend with you? (in minutes) | 🖎 |  | cg_mh_timespent |
| D26 | How much did you (or someone else) pay for this, including travel expenditure? | 🖎 |  | cg_mh_cost |
| D27 | How long did it take to travel there and back? (in minutes) | 🖎 |  | cg_mh_travel |
| The following section pertains to other services not mentioned above | | | | |
| D28 | Have you used, or had contact with, any other people or services in the past 3 months (not including what you reported above)? | No | 0 | cg_outpatient_other |
|  |  | Yes [Answer D29] | 1 |  |
|  |  | Prefer not to respond | 99 |  |
| D29 | Total number of visits in the past 3 months | 🖎 |  | cg_other_times |
| Other visit: Section repeated by number reported under [cg_other_times] | | | | |
| D30 | Type of person seen/service sought | Parenting support | 1 | cg_other_type |
|  |  | Fortune teller | 2 |  |
|  |  | Motivational speaker | 3 |  |
|  |  | Debt advisor | 4 |  |
|  |  | Immigration counselling | 5 |  |
|  |  | Addiction support | 6 |  |
|  |  | Career advisor | 7 |  |
|  |  | Domestic violence support | 8 |  |
|  |  | Person who helps with household tasks | 9 |  |
|  |  | Benefit advisor | 10 |  |
|  |  | Life coach | 11 |  |
|  |  | Other health worker | 77 |  |
|  |  | Don't know | 88 |  |
|  |  | Prefer not to respond | 99 |  |
| D31 | Other type of practitioner seen | 🖎 |  | cg_other _typeo |
| D32 | How long did the visit last (including waiting time)? (in minutes) | 🖎 |  | cg_other _long |
| D33 | How much did you (or someone else) pay for this, including travel expenditure? | 🖎 |  | cg_other_cost |
| D34 | How long did it take to travel there and back? (in minutes) | 🖎 |  | cg_other_travel |

| E. Client Service Receipt Inventory (Adapted) – Part 1: Adolescent (Beecham & Knapp, 2001)  *[All follow-up assessments]* | | | | |
| --- | --- | --- | --- | --- |
| Instructions:  *“These next questions are about the services that your index child might have used over the past few months. Every question is important, please try your best to respond each of them. If you do not want to answer a question, please let us know. Remember that all your answers are confidential”* | | | | |
| E1 | Has your index child attended the emergency department of a hospital in the past 6 months? | No | 0 | ado_emergency |
|  |  | Yes [Answer E2- E4] | 1 |  |
|  |  | Prefer not to respond | 99 |  |
| E2 | How many times did your index child attend the emergency department of a hospital in the past 6 months? | 🖎 |  | ado_emergency_times |
| E3 | What was the emergency? | 🖎 |  | ado_emergency_why |
| E4 | Did you or someone else pay for this? | No | 0 | ado_emergency_pay |
|  |  | Yes [Answer E5] | 1 |  |
|  |  | Prefer not to respond | 99 |  |
| E5 | How much did you or someone else pay? | 🖎 |  | ado_emergency_cost |
| E6 | Has your index child been admitted to hospital (i.e. staying at least one night) in the past 6 months? | No | 0 | ado_admitted |
|  |  | Yes [Answer E6-E8] | 1 |  |
|  |  | Prefer not to respond | 99 |  |
| E7 | How many days was your index child in hospital altogether? | 🖎 |  | ado_admitted_days |
| E8 | What were the reasons for your index child’s hospital stay? | Infectious disease (e.g. HIV, malaria, TB) | 1 | ado_admitted_why |
|  |  | Maternal / perinatal condition | 2 |  |
|  |  | Acute condition (e.g. flu, cough) | 3 |  |
|  |  | Injury | 4 |  |
|  |  | Sleep problems | 5 |  |
|  |  | Depression or anxiety | 6 |  |
|  |  | Alcohol problems | 7 |  |
|  |  | Other mental health problems | 8 |  |
|  |  | Other chronic disease (e.g. heart, diabetes) | 9 |  |
|  |  | Other reason [Answer E9] | 77 |  |
|  |  | Don’t know | 88 |  |
|  |  | Prefer not to respond | 99 |  |
| E9 | What other reason were they admitted to hospital? | 🖎 |  | ado_admitted_whyo |
| E10 | Did you or someone else pay for this? | No | 0 | ado_admitted_pay |
|  |  | Yes [Answer E11] | 1 |  |
|  |  | Prefer not to respond | 99 |  |
| E11 | How much did you or someone else pay? | 🖎 |  | ado_admitted_cost |
| The following section pertains to service use for physical health | | | | |
| E12 | Has your index child seen someone in the hospital or in the community for their physical health in the past 3 months? | No | 0 | ado_outpatient_physical |
|  |  | Yes [Answer E13] | 1 |  |
|  |  | Prefer not to respond | 99 |  |
| E13 | Total number of visits in the past 3 months | 🖎 |  | ado_phys_times |
| Visit for physical health: Section repeated by number reported under [ado_phys_times] | | | | |
| E14 | Type of practitioner seen | Traditional healer/spiritualist/herbalist | 1 | ado_phys_type |
|  |  | Specialist doctor | 2 |  |
|  |  | Health professional at school | 3 |  |
|  |  | Community health worker | 4 |  |
|  |  | Nurse/midwife | 5 |  |
|  |  | Pharmacist | 6 |  |
|  |  | General medical doctor | 7 |  |
|  |  | Social worker (Female Community Health Volunteer) [Ne] | 8 |  |
|  |  | Occupational therapist | 9 |  |
|  |  | Physiotherapist | 10 |  |
|  |  | Other health worker | 77 |  |
|  |  | Don't know | 88 |  |
|  |  | Prefer not to respond | 99 |  |
| E15 | Other type of practitioner seen | 🖎 |  | ado_phys_typeo |
| E16 | How long did the visit last (including waiting time)? (in minutes) | 🖎 |  | ado_phys_long |
| E17 | How much time did the practitioner spend with your index child? (in minutes) | 🖎 |  | ado_phys_timespent |
| E18 | How much did you (or someone else) pay for this, including travel expenditure? | 🖎 |  | ado_phys_cost |
| E19 | How long did it take to travel there and back? (in minutes) | 🖎 |  | ado_phys_travel |
| The following section pertains to service use for mental health | | | | |
| E20 | Has your index child seen someone in the hospital or in the community for their mental health or wellbeing in the past 3 months? | No | 0 | ado_outpatient_mh |
|  |  | Yes [Answer E21] | 1 |  |
|  |  | Prefer not to respond | 99 |  |
| E21 | Total number of visits in the past 3 months | 🖎 |  | ado_mh_times |
| Visit for mental health: Section repeated by number reported under [ado_mh_times] | | | | |
| E22 | Type of practitioner seen | General medical doctor | 1 | ado_mh_type |
|  |  | Psychiatrist | 2 |  |
|  |  | Psychologist | 3 |  |
|  |  | School/student counsellor | 4 |  |
|  |  | Psychosocial counsellor | 5 |  |
|  |  | Psychiatric nurse | 6 |  |
|  |  | Psychiatric clinical officer | 7 |  |
|  |  | Social worker | 8 |  |
|  |  | Religious/spiritual/traditional healer | 9 |  |
|  |  | Specialist doctor (other than psychiatrist) | 10 |  |
|  |  | Occupational therapist | 11 |  |
|  |  | Other practitioner | 77 |  |
|  |  | Don't know | 88 |  |
|  |  | Prefer not to respond | 99 |  |
| E23 | Other type of practitioner seen | 🖎 |  | ado_mh_typeo |
| E24 | How long did the visit last (including waiting time)? (in minutes) | 🖎 |  | ado_mh_long |
| E25 | How much time did the practitioner spend with your index child? (in minutes) | 🖎 |  | ado_mh_timespent |
| E26 | How much did you (or someone else) pay for this, including travel expenditure? | 🖎 |  | ado_mh_cost |
| E27 | How long did it take to travel there and back? (in minutes) | 🖎 |  | ado_mh_travel |
| The following section pertains to other services not mentioned above | | | | |
| E28 | Has your index child used, or had contact with, any other people or services in the past 3 months (not including what you reported above)? | No | 0 | ado_outpatient_other |
|  |  | Yes [Answer E29] | 1 |  |
|  |  | Prefer not to respond | 99 |  |
| E29 | Total number of visits in the past 3 months | 🖎 |  | ado_other_times |
| Other visit: Section repeated by number reported under [ado_other_times] | | | | |
| E30 | Type of person seen/service sought | Child or youth club | 1 | ado_other_type |
|  |  | Fortune teller | 2 |  |
|  |  | Motivational speaker | 3 |  |
|  |  | Debt advisor | 4 |  |
|  |  | School advisor/parents teacher association (PTA) | 5 |  |
|  |  | Addiction support | 6 |  |
|  |  | Police personnel | 7 |  |
|  |  | Prison or arrest unit | 8 |  |
|  |  | Life counsellor, coach or mentor | 9 |  |
|  |  | Other health worker | 77 |  |
|  |  | Don't know | 88 |  |
|  |  | Prefer not to respond | 99 |  |
| E31 | Other type of practitioner seen | 🖎 |  | ado_other _typeo |
| E32 | How long did the visit last (including waiting time)? (in minutes) | 🖎 |  | ado_other _long |
| E33 | How much did you (or someone else) pay for this, including travel expenditure? | 🖎 |  | ado_other_cost |
| E34 | How long did it take to travel there and back? (in minutes) | 🖎 |  | ado_other_travel |
| End of repeat section | | | | |
| E35 | Has your index child missed school or work in the last month, for example, because you did not feel like going or something else came up? | No | 0 | ado_schoolmissed |
|  |  | Yes [Answer E35-E37] | 1 |  |
|  |  | Prefer not to respond | 99 |  |
| E35 | How many days did they miss? | 🖎 |  | ado_schoolmissed_days |
| E36 | What type of activity did they miss? | Education | 1 | ado_schoolmissed_activity |
|  |  | Paid work | 2 |  |
|  |  | Unpaid work | 3 |  |
|  |  | Other | 77 |  |
|  |  | Don't know | 88 |  |
|  |  | Prefer not to respond | 99 |  |
| E37 | How much income (money) did they lose by missing this activity? | 🖎 |  | ado_schoolmissed_cost |

| F. Roster  *[Unless otherwise stated, baseline and 18-month post-baseline assessments only]* | | | | | | | |
| --- | --- | --- | --- | --- | --- | --- | --- |
| Instructions: “*Every question is important, please try your best to respond each of them. If you do not want to answer a question, please let us know. Remember that all your answers are confidential.*” | | | | | | | |
| F1 | How many children under the age of 15 are living in your household? | 🖎 | | | | children | |
| F2 | How many individuals aged 15 or more are living in your household? | 🖎 | | | | adults | |
| F3 | *[Baseline only]*  Has any child under 5 passed away in the household in the past year? | No | | 0 | | child_death | |
|  |  | Yes | | 1 | |  |  |
|  |  | Prefer not to respond | | 99 | |  |  |
| F3a | *[Nepal only]*  Has any child (under 18 years old) passed away in the household in the past five years? | No | | 0 | | child_death18 | |
|  |  | Yes | | 1 | |  |  |
|  |  | Prefer not to respond | | 99 | |  |  |
| F3b | *[All follow-up assessments]*  Has any child under 5 passed away in the household in the past 6 months? | No | | 0 | | child_death_6m | |
|  |  | Yes | | 1 | |  |  |
|  |  | Prefer not to respond | | 99 | |  |  |
| **Child Roster: Section repeated by number reported under [children] _#**  *“I'm going to ask you questions about all children under 15 living in this household. We are going to go through each child one by one, starting with the youngest.”* | | | | | | | |
| F4 | What is the name of the child? | 🖎 | | | | child_name | |
| F5 | How old is [child_name]? | 🖎 | | | | child_age | |
| F6 | What is [child_name]'s preferred gender identity? | Male | | 0 | | child_gender | |
|  |  | Female | | 1 | |  |  |
|  |  | Non-binary | | 2 | |  |  |
|  |  | Other [Answer F7] | | 77 | |  |  |
|  |  | Prefer not respond | | 99 | |  |  |
| F7 | What other gender? | 🖎 | |  | | child_gendero | |
| F8 | What is [child_name]'s relationship to the household head? | Daughter/son | | 1 | | child_rel_head | |
|  |  | Grandchild | | 2 | |  |  |
|  |  | Brother/sister | | 3 | |  |  |
|  |  | Niece/Nephew | | 4 | |  |  |
|  |  | Cousin | | 5 | |  |  |
|  |  | Other in-law family member | | 11 | |  |  |
|  |  | Non-family member | | 12 | |  |  |
|  |  | Other [Answer F9] | | 77 | |  |  |
|  |  | Don’t know | | 88 | |  |  |
|  |  | Prefer not to respond | | 99 | |  |  |
| F9 | What other relationship? | 🖎 | |  | | child_rel_heado | |
| F10 | What is [child_name]'s relationship to the index child? | The child is the index child | | 0 | | child_rel_index | |
|  |  | Daughter/son | | 1 | |  |  |
|  |  | Brother/sister | | 3 | |  |  |
|  |  | Niece/Nephew | | 4 | |  |  |
|  |  | Cousin | | 5 | |  |  |
|  |  | Other in-law family member | | 11 | |  |  |
|  |  | Non-family member | | 12 | |  |  |
|  |  | Other [Answer F11] | | 77 | |  |  |
|  |  | Don’t know | | 88 | |  |  |
|  |  | Prefer not to respond | | 99 | |  |  |
| F11 | What other relationship? | 🖎 | |  | | child_rel_indexo | |
| F12 | *[Colombia only]* Does [child_name] know how to read and write? | No | | 0 | | child_literacy | |
|  |  | Yes | | 1 | |  |  |
|  |  | Prefer not to respond | | 99 | |  |  |
| F13 | *[If child_age > 5]* Is [child_name] currently attending school? | No | | 0 | | child_currentedu | |
|  |  | Yes | | 1 | |  |  |
|  |  | Don’t know | | 88 | |  |  |
|  |  | Prefer not to respond | | 99 | |  |  |
| F14 | *[Colombia only]* What grade is [child_name] currently enrolled in? | Grade 3 | | 1 | | child_grade | |
|  |  | Grade 4 | | 2 | |  |  |
|  |  | Grade 5 | | 3 | |  |  |
|  |  | Grade 6 | | 4 | |  |  |
|  |  | Grade 7 | | 5 | |  |  |
|  |  | Grade 8 | | 6 | |  |  |
|  |  | Grade 9 | | 7 | |  |  |
|  |  | Grade 10 | | 8 | |  |  |
|  |  | Grade 11 | | 9 | |  |  |
|  |  | First-second semester of higher or technical education | | 10 | |  |  |
|  |  | Third-fourth semester of higher or technical education | | 11 | |  |  |
|  |  | Fifth-sixth semester of higher or technical education | | 12 | |  |  |
|  |  | Seventh-eighth semester of higher or technical education | | 13 | |  |  |
|  |  | Ninth-tenth semester of higher or technical education | | 14 | |  |  |
|  |  | Other [Answer F15] | | 77 | |  |  |
|  |  | Prefer not to answer | | 99 | |  |  |
| F15 | Please specify what other grade | 🖎 | |  | | child_grade_o | |
| F16 | *[Colombia and Nepal]* How many years of education has [child_name] completed? | 🖎 | |  | | child_eduyrs | |
|  |  | Don’t know | | 88 | |  |  |
|  |  | Prefer not to respond | | 99 | |  |  |
| F17 | *[Colombia only]* Last week, did [child_name] work for pay for one or more hours? (This includes occasional work for pay or in kind, in exchange for food or accommodation)  [Note: This refers to being employed or working for pay]. | No | | 0 | | child_employment | |
|  |  | Yes | | 1 | |  |  |
|  |  | Prefer not to respond | | 99 | |  |  |
| F18 | *[Colombia only]* In the past week, did [child_name] engage in any other type of economic activity such as making, repairing or selling things, doing activities in exchange for a tip, or any other income-generating activity?  [Note: This refers to independent economic activities or that the person does not officially classify as "work" and does not include housework]". | No | | 0 | | child_labour_ind | |
|  |  | Yes | | 1 | |  |  |
|  |  | Prefer not to respond | | 99 | |  |  |
| F19 | *[Colombia only]* In the past week, did [child_name] do any unpaid help or companionship activity for a family member or other person? (For example, did they help or accompany a family member or other person who works as an employee or labourer in shoe, mechanic or carpentry workshops, in domestic service, in transport services, in the production of agricultural products, in cleaning or guarding your family business, or in any other activity that generates income for the family?) | No | | 0 | | child_laber_comp | |
|  |  | Yes | | 1 | |  |  |
|  |  | Prefer not to respond | | 99 | |  |  |
| F20 | *[If child_age 0-5]* Where does [child_name] spend most of the week? | Community home | | 1 | | child_timespent | |
|  |  | Nursery | | 2 | |  |  |
|  |  | Pre-school | | 3 | |  |  |
|  |  | Under the care of responsible adult | | 4 | |  |  |
|  |  | Taken to work by caregiver | | 5 | |  |  |
|  |  | Stays at home alone | | 6 | |  |  |
|  |  | Other [Answer F21] | | 77 | |  |  |
|  |  | Don’t know | | 88 | |  |  |
|  |  | Prefer not to respond | | 99 | |  |  |
| F21 | Where else does [child_name] spend most of the week? | 🖎 | |  | | child_timespento | |
| F22 | *[If child_age 0-5]* Does [child_name] have at least two meals a day? | No | | 0 | | child_meals | |
|  |  | Yes | | 1 | |  |  |
|  |  | Don’t know | | 88 | |  |  |
|  |  | Prefer not to respond | | 99 | |  |  |
| F23 | Is [child_name] affiliated to any health insurance or medical aid plan? | No | | 0 | | child_insurance | |
|  |  | Yes | | 1 | |  |  |
|  |  | Don’t know | | 88 | |  |  |
|  |  | Prefer not to respond | | 99 | |  |  |
| **Adult Roster: Section repeated by number reported under [adult] _#**  *“I'm going to ask you questions about all adults and children aged 15 and above living in this household. We are going to go through each person one by one, starting with the youngest.”* | | | | | | | |
| F24 | What is the name of the individual? | | 🖎 | |  | | adult_name |
| F25 | Is [adult_name] the caregiver completing this questionnaire? | | No | | 0 | | adult_cg |
|  |  |  | Yes | | 1 | |  |
| F26 | How old is [adult_name]? | | 🖎 | |  | | adult_age |
| F27 | What is [adult_name]'s preferred gender identity? | | Male | | 0 | | adult_gender |
|  |  |  | Female | | 1 | |  |
|  |  |  | Non-binary | | 2 | |  |
|  |  |  | Other [Answer F28] | | 77 | |  |
|  |  |  | Prefer not to respond | | 99 | |  |
| F28 | What other gender? | | 🖎 | |  | | adult_gendero |
| F29 | What is [adult_name]'s relationship to the household head? | | He/she is the household head | | 0 | | adult_rel_head |
|  |  |  | Daughter/son | | 1 | |  |
|  |  |  | Grandchild | | 2 | |  |
|  |  |  | Brother/sister | | 3 | |  |
|  |  |  | Niece/Nephew | | 4 | |  |
|  |  |  | Cousin | | 5 | |  |
|  |  |  | Mother/father | | 6 | |  |
|  |  |  | Spouse | | 7 | |  |
|  |  |  | Daugher-in-law/Son-in-law | | 8 | |  |
|  |  |  | Uncle/aunt | | 9 | |  |
|  |  |  | Grandparent | | 10 | |  |
|  |  |  | Other in-law family member | | 11 | |  |
|  |  |  | Non-family member | | 12 | |  |
|  |  |  | Other | | 77 | |  |
|  |  |  | Don’t know | | 88 | |  |
|  |  |  | Prefer not to respond | | 99 | |  |
| F30 | What other relationship? | | 🖎 | |  | | adult_rel_heado |
| F31 | What is [adult_name]'s relationship to the index child? | | He/she is the index child | | 0 | | adult_rel_index |
|  |  |  | Brother/sister | | 3 | |  |
|  |  |  | Cousin | | 5 | |  |
|  |  |  | Mother/father | | 6 | |  |
|  |  |  | Spouse | | 7 | |  |
|  |  |  | Uncle/aunt | | 9 | |  |
|  |  |  | Grandparent | | 10 | |  |
|  |  |  | Other in-law family member | | 11 | |  |
|  |  |  | Non-family member | | 12 | |  |
|  |  |  | Other | | 77 | |  |
|  |  |  | Don’t know | | 88 | |  |
|  |  |  | Prefer not to respond | | 99 | |  |
| F32 | What other relationship? | | 🖎 | |  | | adult_rel_indexo |
| F33 | *[Colombia only]* Does [adult_name] know how to read and write? | | No | | 0 | | adult_literacy |
|  |  |  | Yes | | 1 | |  |
|  |  |  | Prefer not to respond | | 99 | |  |
| F34 | Is [adult_name] currently attending school, technical study or university? | | No | | 0 | | adult_school |
|  |  |  | Yes [Answer F35] | | 1 | |  |
|  |  |  | Don’t know | | 88 | |  |
|  |  |  | Prefer not to respond | | 99 | |  |
| F35 | *[Colombia only]* What grade is [adult_name] currently enrolled in? | | Grade 3 | | 1 | | adult_grade |
|  |  |  | Grade 4 | | 2 | |  |
|  |  |  | Grade 5 | | 3 | |  |
|  |  |  | Grade 6 | | 4 | |  |
|  |  |  | Grade 7 | | 5 | |  |
|  |  |  | Grade 8 | | 6 | |  |
|  |  |  | Grade 9 | | 7 | |  |
|  |  |  | Grade 10 | | 8 | |  |
|  |  |  | Grade 11 | | 9 | |  |
|  |  |  | First-second semester of higher or technical education | | 10 | |  |
|  |  |  | Third-fourth semester of higher or technical education | | 11 | |  |
|  |  |  | Fifth-sixth semester of higher or technical education | | 12 | |  |
|  |  |  | Seventh-eigth semester of higher or technical education | | 13 | |  |
|  |  |  | Ninth-tenth semester of higher or technical education | | 14 | |  |
|  |  |  | Other [Answer F36] | | 77 | |  |
|  |  |  | Prefer not to answer | | 99 | |  |
| F36 | Please specify what other grade | | 🖎 | |  | | adult_grade_o |
| F37 | How many years of education has [adult_name] completed? | | 🖎 | |  | | adult_eduyrs |
|  |  |  | Don’t know | | 88 | |  |
|  |  |  | Prefer not to respond | | 99 | |  |
| F38 | *[Colombia only, if adult_age<=18]* Last week, did [adult_name] work for pay for one or more hours? (This includes occasional work for pay or in kind, in exchange for food or accommodation)  [Note: This refers to being employed or working for pay]. | | No | | 0 | | adult_employment |
|  |  |  | Yes | | 1 | |  |
|  |  |  | Prefer not to respond | | 99 | |  |
| F39 | *[Colombia only, if adult_age<=18]* In the past week, did [adult_name] engage in any other type of economic activity such as making, repairing or selling things, doing activities in exchange for a tip, or any other income-generating activity?  [Note: This refers to independent economic activities or that the person does not officially classify as "work" and does not include housework]". | | No | | 0 | | adult_labour_ind |
|  |  |  | Yes | | 1 | |  |
|  |  |  | Prefer not to respond | | 99 | |  |
| F40 | *[Colombia only, if adult_age<=18]* In the past week, did [adult_name] do any unpaid help or companionship activity for a family member or other person? (For example, did they help or accompany a family member or other person who works as an employee or labourer in shoe, mechanic or carpentry workshops, in domestic service, in transport services, in the production of agricultural products, in cleaning or guarding your family business, or in any other activity that generates income for the family?) | | No | | 0 | | adult_labour_comp_ |
|  |  |  | Yes | | 1 | |  |
|  |  |  | Prefer not to respond | | 99 | |  |
| F41 | What is [adult_name]’s employment status? | | Unemployed [Answer F43] | | 0 | | adult_emp |
|  |  |  | Salaried job | | 1 | |  |
|  |  |  | Self-employed (e.g., own business, small trader, etc.) | | 2 | |  |
|  |  |  | Independent services | | 3 | |  |
|  |  |  | Daily wager | | 4 | |  |
|  |  |  | Homemaker | | 5 | |  |
|  |  |  | Student | | 6 | |  |
|  |  |  | Other [Answer F42] | | 77 | |  |
|  |  |  | Don't know | | 88 | |  |
|  |  |  | Prefer not to respond | | 99 | |  |
| F42 | What other employment status? | | 🖎 | |  | | adult_empo |
| F43 | How long has [adult_name] been unemployed for? | | Unemployed for 12 months or less | | 1 | | adult_unemp |
|  |  |  | Unemployed for more than 12 months | | 2 | |  |
|  |  |  | Don't know | | 88 | |  |
|  |  |  | Prefer not to respond | | 99 | |  |
| F44 | *[If answer 1-4 to F41]*  Does [adult_name] contribute to a pension fund? | | No | | 0 | | adult_pension |
|  |  |  | Yes | | 1 | |  |
|  |  |  | Don’t know | | 88 | |  |
|  |  |  | Prefer not to respond | | 99 | |  |
| F45 | Is [adult_name] affiliated to any health insurance/medical aid plan? | | No | | 0 | | adult_insurance |
|  |  |  | Yes | | 1 | |  |
|  |  |  | Don’t know | | 88 | |  |
|  |  |  | Prefer not to respond | | 99 | |  |

| G - Patient Health Questionnaire – Adult version | | | | |
| --- | --- | --- | --- | --- |
| Instructions:  “*Please ask the individual how often have they been bothered by the following over the past 2 weeks.*  *Every question is important, please try your best to respond each of them. If you do not want to answer a question, please let us know. Remember that all your answers are confidential.*” | | | | |
| G1 | How often have you had little interest or pleasure in doing things? | Never | 0 | phq9_1 |
|  |  | Almost never | 1 |  |
|  |  | Sometimes | 2 |  |
|  |  | Often | 3 |  |
|  |  | Prefer not to respond | 99 |  |
| G2 | How often have you been feeling down, depressed, or hopeless? | Never | 0 | phq9_2 |
|  |  | Almost never | 1 |  |
|  |  | Sometimes | 2 |  |
|  |  | Often | 3 |  |
|  |  | Prefer not to respond | 99 |  |
| G3 | How often have you had trouble sleeping or staying asleep, or sleeping too much? | Never | 0 | phq9_3 |
|  |  | Almost never | 1 |  |
|  |  | Sometimes | 2 |  |
|  |  | Often | 3 |  |
|  |  | Prefer not to respond | 99 |  |
| G4 | How often have you been feeling tired or having little energy? | Never | 0 | phq9_4 |
|  |  | Almost never | 1 |  |
|  |  | Sometimes | 2 |  |
|  |  | Often | 3 |  |
|  |  | Prefer not to respond | 99 |  |
| G5 | How often have you had poor appetite or been overeating? (i.e., eating more or less than what you usually eat) | Never | 0 | phq9_5 |
|  |  | Almost never | 1 |  |
|  |  | Sometimes | 2 |  |
|  |  | Often | 3 |  |
|  |  | Prefer not to respond | 99 |  |
| G6 | How often have you been feeling bad about yourself — or that you are a failure or have let yourself or your family down? | Never | 0 | phq9_6 |
|  |  | Almost never | 1 |  |
|  |  | Sometimes | 2 |  |
|  |  | Often | 3 |  |
|  |  | Prefer not to respond | 99 |  |
| G7 | How often have you had trouble concentrating on things, such as reading the newspaper or watching television? | Never | 0 | phq9_7 |
|  |  | Almost never | 1 |  |
|  |  | Sometimes | 2 |  |
|  |  | Often | 3 |  |
|  |  | Prefer not to respond | 99 |  |
| G8 | How often have you been moving or speaking so slowly that other people could have noticed? | Never | 0 | phq9_8 |
|  |  | Almost never | 1 |  |
|  |  | Sometimes | 2 |  |
|  |  | Often | 3 |  |
|  |  | Prefer not to respond | 99 |  |
| G9 | How often have you been so fidgety or restless that you have been moving around a lot more than usual? | Never | 0 | phq9_8_bis |
|  |  | Almost never | 1 |  |
|  |  | Sometimes | 2 |  |
|  |  | Often | 3 |  |
|  |  | Prefer not to respond | 99 |  |
| G10 | How often have you had thoughts that you would be better off dead or of hurting yourself in some way? | Never | 0 | phq9_9 |
|  |  | Almost never | 1 |  |
|  |  | Sometimes | 2 |  |
|  |  | Often | 3 |  |
|  |  | Prefer not to respond | 99 |  |
| G11 | Total PHQ-9 score |  |  | totalphq |
| G12 | Participant screens positive on the PHQ-9 |  |  | cgdepressed |

| H. Alabama Parenting Questionnaire | | | | |
| --- | --- | --- | --- | --- |
| Instructions:  “*The following are a numbers of statements about your family. Please rate each item as to how often it typically occurs in your home. The possible answers are Never (1), Almost never (2), Sometimes (3), Often (4), Always (5).*  *Every question is important, please try your best to respond each of them. If you do not want to answer a question, please let us know. Remember that all your answers are confidential.*” | | | | |
| H1 | How much do you talk to your children like being their friends? | Never | 1 | apq_1 |
|  |  | Almost never | 2 |  |
|  |  | Sometimes | 3 |  |
|  |  | Often | 4 |  |
|  |  | Always | 5 |  |
|  |  | Prefer not to respond | 99 |  |
| H2 | How much do you help your children to be engaged in the activities that they are involved in ? | Never | 1 | apq_2 |
|  |  | Almost never | 2 |  |
|  |  | Sometimes | 3 |  |
|  |  | Often | 4 |  |
|  |  | Always | 5 |  |
|  |  | Prefer not to respond | 99 |  |
| H3 | How much do you play games with your children or have fun time with them? (For example, badminton, table tennis, football, etc) | Never | 1 | apq_3 |
|  |  | Almost never | 2 |  |
|  |  | Sometimes | 3 |  |
|  |  | Often | 4 |  |
|  |  | Always | 5 |  |
|  |  | Prefer not to respond | 99 |  |
| H4 | How much do you ask your children about their daily activities in school? | Never | 1 | apq_4 |
|  |  | Almost never | 2 |  |
|  |  | Sometimes | 3 |  |
|  |  | Often | 4 |  |
|  |  | Always | 5 |  |
|  |  | Prefer not to respond | 99 |  |
| H5 | How much do you help your child with their homework or other things related to studies? | Never | 1 | apq_5 |
|  |  | Almost never | 2 |  |
|  |  | Sometimes | 3 |  |
|  |  | Often | 4 |  |
|  |  | Always | 5 |  |
|  |  | Prefer not to respond | 99 |  |
| H6 | How much do you ask your children about what activities they have planned for the next 2-3 days? (For example, what activities are going to be there in school, what will they do after school ends, sports, roaming around, etc) | Never | 1 | apq_6 |
|  |  | Almost never | 2 |  |
|  |  | Sometimes | 3 |  |
|  |  | Often | 4 |  |
|  |  | Always | 5 |  |
|  |  | Prefer not to respond | 99 |  |
| H7 | How much do you take your children out to do the activities that they like doing? | Never | 1 | apq_7 |
|  |  | Almost never | 2 |  |
|  |  | Sometimes | 3 |  |
|  |  | Often | 4 |  |
|  |  | Always | 5 |  |
|  |  | Prefer not to respond | 99 |  |
| H8 | How much do you talk to your children about their friends? | Never | 1 | apq_8 |
|  |  | Almost never | 2 |  |
|  |  | Sometimes | 3 |  |
|  |  | Often | 4 |  |
|  |  | Always | 5 |  |
|  |  | Prefer not to respond | 99 |  |
| H9 | How much does your children help you to plan family gatherings and programs? | Never | 1 | apq_9 |
|  |  | Almost never | 2 |  |
|  |  | Sometimes | 3 |  |
|  |  | Often | 4 |  |
|  |  | Always | 5 |  |
|  |  | Prefer not to respond | 99 |  |
| H10 | How much do you participate in the school meetings between parents and teachers? | Never | 1 | apq_10 |
|  |  | Almost never | 2 |  |
|  |  | Sometimes | 3 |  |
|  |  | Often | 4 |  |
|  |  | Always | 5 |  |
|  |  | Prefer not to respond | 99 |  |
| H11 | How much do you let your child know about it when your child does something good? | Never | 1 | apq_11 |
|  |  | Almost never | 2 |  |
|  |  | Sometimes | 3 |  |
|  |  | Often | 4 |  |
|  |  | Always | 5 |  |
|  |  | Prefer not to respond | 99 |  |
| H12 | How much do you reward your child when they obey what you said? | Never | 1 | apq_12 |
|  |  | Almost never | 2 |  |
|  |  | Sometimes | 3 |  |
|  |  | Often | 4 |  |
|  |  | Always | 5 |  |
|  |  | Prefer not to respond | 99 |  |
| H13 | How much do you show love or hug your children when they do good work? | Never | 1 | apq_13 |
|  |  | Almost never | 2 |  |
|  |  | Sometimes | 3 |  |
|  |  | Often | 4 |  |
|  |  | Always | 5 |  |
|  |  | Prefer not to respond | 99 |  |
| H14 | When your children help with household work and you feel good about it, how much do you say about it to your children? | Never | 1 | apq_14 |
|  |  | Almost never | 2 |  |
|  |  | Sometimes | 3 |  |
|  |  | Often | 4 |  |
|  |  | Always | 5 |  |
|  |  | Prefer not to respond | 99 |  |
| H15 | How much do you slap your children on their cheek when they make a mistake? | Never | 1 | apq_15 |
|  |  | Almost never | 2 |  |
|  |  | Sometimes | 3 |  |
|  |  | Often | 4 |  |
|  |  | Always | 5 |  |
|  |  | Prefer not to respond | 99 |  |
| H16 | How much do you scold or shout at your children when they make a mistake? | Never | 1 | apq_16 |
|  |  | Almost never | 2 |  |
|  |  | Sometimes | 3 |  |
|  |  | Often | 4 |  |
|  |  | Always | 5 |  |
|  |  | Prefer not to respond | 99 |  |

| I. Contamination | | | | |
| --- | --- | --- | --- | --- |
| I1 | Have you heard about this study from other family members, friends or other people? | No | 0 | cg_aware |
|  |  | Yes [Answer H2 and H3] | 1 |  |
|  |  | Don’t know | 88 |  |
|  |  | Prefer not to respond | 99 |  |
| I2 | Can you tell me a bit more about what they said? | 🖎 | | cg_aware_describe |

| J. Blinding | | | | |
| --- | --- | --- | --- | --- |
| J1 | [To fieldworker] Please indicate which intervention arm you think the participant is allocated to. | Control | 0 | blinding |
|  |  | Self-regulation intervention | 1 |  |
|  |  | Economic intervention | 2 |  |
|  |  | Combined (self-regulation and economic) intervention | 3 |  |
